# Unlocking intrinsically chiral bipyrenyl-based aggregation-induced emission luminogens: circularly polarized luminescence and dynamic chirality amplification

**DOI:** 10.1039/d5sc08358c

**Published:** 2026-01-07

**Authors:** Zhixin Xie, Junpeng Deng, Dan Liu, Jieyu Lin, Tao Jiang, Xiaohui Wang, Wei Liu, Lin Ma, Fengyan Song, Zuping Xiong, Junru Chen, Jianyu Zhang, Carl Redshaw, Zujin Zhao, Xing Feng, Ben Zhong Tang

**Affiliations:** a Guangdong Provincial Key Laboratory of Functional Soft Condensed Matter, School of Materials and Energy, Guangdong University of Technology Guangzhou 510006 P. R. China hyxhn@sina.com; b School of Physics and Optoelectronic Engineering, Guangdong Provincial Key Laboratory of Sensing Physics and System Integration Applications, Institute for Frontier Physics and Advanced Instruments, Guangdong University of Technology Guangzhou 510006 P. R. China malin@gdut.edu.cn; c Guangdong Basic Research Center of Excellence for Aggregate Science, School of Science and Engineering, The Chinese University of Hong Kong Shenzhen Guangdong 518172 China tangbenz@cuhk.edu.cn; d Center of Excellence for Environmental Safety and Biological Effects, Beijing Key Laboratory for Green Catalysis and Separation, Department of Chemistry, College of Chemistry and Life Science, Beijing University of Technology Beijing China fengyansong@bjut.edu.cn; e State Key Laboratory (SKL) of Biobased Transportation Fuel Technology, Department of Polymer Science and Engineering, Zhejiang University Hangzhou 310058 China zhangjianyu@zju.edu.cn; f Department of Chemistry, Graduate School of Science, Tokyo Metropolitan University 1-1 Minami Osawa, Hachioji Tokyo 1920397 Japan; g State Key Laboratory of Luminescent Materials and Devices, Guangdong Provincial Key Laboratory of Luminescence from Molecular Aggregates, South China University of Technology Guangzhou 510640 P. R. China

## Abstract

Understanding chiral dynamic mechanisms (from chirality generation and transfer, and amplification) is crucial for circularly polarized luminescent (CPL) materials. Herein, intrinsically chiral bipyrenyl-based enantiomers, *R-*5 and *S-*5, were first synthesized as model compounds to gain a deeper insight into their chiroptical properties and chirality amplification mechanisms. These enantiomers not only exhibit typical aggregation-induced emission (AIE) with a high solid-state fluorescence efficiency up to 0.66, but also display significant chirality amplification upon aggregation, with amplified |*g*_CD_| from 4.73 × 10^−5^ (10^−7^ M) to 7.34 × 10^−3^ (10^−3^ M), and |*g*_lum_| values up to 4.68 × 10^−4^ in the solid state. Morphological and CP-fs-TA studies reveal that the amplified chiroptical properties stem from helical self-assembly and prolonged excited-state chiral conformational reorganization in aggregates. This work establishes a design strategy for high-performance CPL materials by integrating intrinsic chirality, AIE properties, and dynamic chirality amplification mechanisms.

## Abstract 2

This article presents two novel intrinsically chiral bipyrenyl-based enantiomers, *R*-5 and *S*-5, which were first synthesized as model compounds to gain a deeper insight into their chiroptical properties and chirality amplification mechanisms.

## Graphical abstract

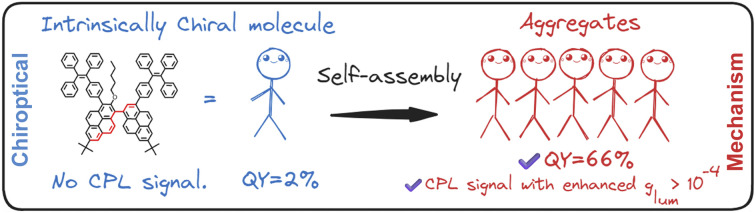

## Introduction

Chirality is ubiquitous in nature,^1^ ranging from microscopic molecules to macroscopic biological entities, and from interstellar space^2^ to our daily lives. To gain a deeper understanding of chiral phenomena, numerous artificial chiral molecules and architectures have been developed through self-assembly,^3,4^ asymmetric synthesis,^5^ and chiral sources to form central, axial, planar, helical, and inherently chiral structures.^6–8^

Circularly polarized luminescence (CPL) arises from chiral luminescent materials or is produced in chiral environments, leading to left and right-circularly polarized light.^9^ High-performance CPL materials have attracted tremendous attention because of their prospective application in 3D optical displays,^10,11^ information encryption,^12–14^ bioimaging,^15,16^ asymmetric photosynthesis,^17,18^*etc.* As a next-generation optoelectronic material platform, the CPL field faces two key challenges: (1) the development of novel solid-state chiral luminescent materials that simultaneously exhibit high emission efficiency and large dissymmetry factors (*g*_lum_) and (2) elucidating the fundamental principles governing the self-assembly dynamics and chirality transfer and chirality amplification mechanisms. Despite significant advancements in CPL emitters that have been achieved by many research groups,^19–22^ molecular-based CPL emitters still retain high *g*_lum_ values which is at the cost of low *Φ*_f_.^23–26^ This is primarily due to the intrinsic aggregation-caused quenching (ACQ) behaviour. According to theory, the luminescence dissymmetry factor |*g*_lum_| of chiral compounds is evaluated according to the following equation:^9^1
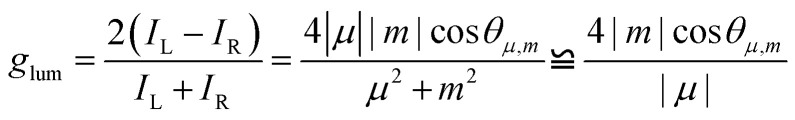
where *I*_L_ and *I*_R_ represent the intensity of right- and left-handed circularly polarized light, and the *µ* and *m* are the transition electric dipole moment and transition magnetic dipole moment vectors, respectively, and *θ*_*µ*,*m*_ is the angle between the *µ* and *m* in the excited state. Thus, as an exceptional CPL molecule, it is crucial to modulate the magnitudes of *µ* and *m*, as well as the angle *θ*_*µ*,*m*_, to achieve a synergistic balance between the *Φ*_f_ and *g*_lum_ values. This optimization framework not only requires rational molecular design of chirality sources but also fundamentally depends on the molecular self-assembly dynamics and chiral dynamic mechanisms across supramolecular architectures.

Currently, the commercial chiral binaphthol (BINOL) is one of the representative chirality sources that has been utilized for the design and synthesis of fascinating axial chiral molecules with potential application in organic optoelectronics, chiral recognition and separation, and data encryption^27,28^ There are over 72 000 related references in the Scifinder database when using “binaphthol” as a keyword when searched over the past two decades. However, as a high-profile intrinsically chiral source (where intrinsically chiral is defined as chirality originating from the inherent asymmetry of a molecular structure or framework, representing a fundamental and innate property of the structure itself), the low fluorescence quantum yield (*Φ*_f_: 0.04) and dissymmetry factor of luminescence (*g*_lum_: 4.7 × 10^−4^) of BINOL have hindered the development of chiral luminescent molecules.^29,30^ Therefore, it is necessary to build new intrinsically chiral skeletons (systems) from the ground up in order to achieve high-efficiency CPL molecules with high *Φ*_f_ and *g*_lum_, and to explore the potential chirality amplification dynamics mechanism.

As a classic member of the polycyclic aromatic hydrocarbon (PAH) family, pyrene is regarded as an ideal building block for constructing CPL molecules.^31,32^ It has inherent advantages in terms of high *Φ*_f_ and long fluorescence lifetime, as well as feasible molecular modification for chiroptical tuning. However, its strong tendency for π–π stacking induces the ACQ effect, leading to significant *Φ*_f_ reduction in the solid state.^31^ For instance, pyrene-based chiral materials, such as pyrene-based [*n*]helicenes,^33^ pyrene-decorated CPL materials^32,34^ and pyrene-based chiral co-assembly architectures^35–37^ still face a critical bottleneck in balancing the emitted *Φ*_f_ and *g*_lum_, as well as requiring complex synthesis. This challenge originates from the competitive mechanism between π–π stacking-induced fluorescence quenching and chiral transfer efficiency. In contrast, Sugiura *et al.* synthesized a novel intrinsically chiral molecule, 1,1′-bipyrene-2,2′-diol, that exhibits an excellent *Φ*_f_ of 0.57 and a |*g*_lum_| of 3.6 × 10^−4^ in solution. Owing to its rigid and expanding π-conjugated bipyrenol molecular architecture, this compound demonstrates superior chiroptical performance compared to commercial BINOL.^29^ This pioneering work opens new avenues for developing intrinsically chiral bipyrenyl-based CPL materials with high *Φ*_f_ and *g*_lum_ values.

Of note, a diverse range of chiral molecules have been designed, leveraging either chiral centers or specific spatial configurations, many of which exhibit considerable *g*_lum_ in the range of 10^−4^ to 10^−2^.^38,39^ Furthermore, when chiral molecules decorated with AIE-groups undergo self-assembly into ordered morphologies in the aggregated state, they achieve a significantly enhanced *g*_lum_ (∼10^−2^–10^−1^).^40,41^ Despite these advances, chiral molecules that simultaneously possess such high emission efficiency remain scarce. Therefore, a deeper comprehension of the regularity governing chirality transfer and amplification mechanisms is crucial for advancing high-performance CPL materials. Traditionally, the investigation of chiral amplification mechanisms has relied on techniques, such as single-crystal X-ray diffraction, scanning electron microscopy (SEM), transmission electron microscopy (TEM), and theoretical calculations. While valuable, these methods often provide indirect or static insights. In fact, the most intuitive approach for unravelling the mechanisms lies in directly probing the electron/energy transfer dynamics between the ground state and the excited state during chirality transfer. This can be achieved using advanced tools like femtosecond time-resolved circularly polarized luminescence (fs-TRCPL) spectroscopy, which offers the potential for real-time observation of these chiral dynamics.^42^

Inspired by the excellent chiroptical properties of 1,1′-bipyrene-2,2′-diol, this article presents a new intrinsically chiral bipyrenyl-based skeleton for constructing two new axial chiral molecules *R-*5 and *S-*5*via* integrating tetraphenylethylene (TPE) units at the 3,3′-positions of the 1,1′-bipyrene-2-ol core. The enantiomers *R-*5 and *S-*5 not only exhibit excellent *Φ*_f_ up to 0.66 in the solid state, but also show concentration-dependent circular dichroism (CD) characteristics and an aggregation-induced CD (AICD) effect, with a |*g*_CD_| from 4.73 × 10^−5^ (10^−7^ M) to 7.34 × 10^−3^ (10^−3^ M), as well as aggregation-induced CPL in the aggregated state with an enhanced *g*_lum_ value from 0 (not detected in THF solution) to 4.68 × 10^−4^ (in the solid state). On the other hand, these enantiomers, *R-*5 and *S-*5, exhibit AIE characteristics with a high quantum yield of up to 0.66 in the solid state. Furthermore, the chirality dynamics of the intrinsically chiral bipyrenyl-based CPL emitters were investigated using circularly polarized femtosecond transient absorption spectroscopy (CP-fs-TA) and scanning electron microscopy, as well as by theoretical calculations. CP-fs-TA reveals that the CPL signal emerging in the solid-state originates from the excitation-induced molecular configurational reorganization, resulting in a hundred-fold order of prolongation of chirality conformation reorganization decay in the thin film (>500 ps) compared to that in solution (<2 ps).

## Results and discussion

### Synthesis and characteristics

**Fig. 1:**
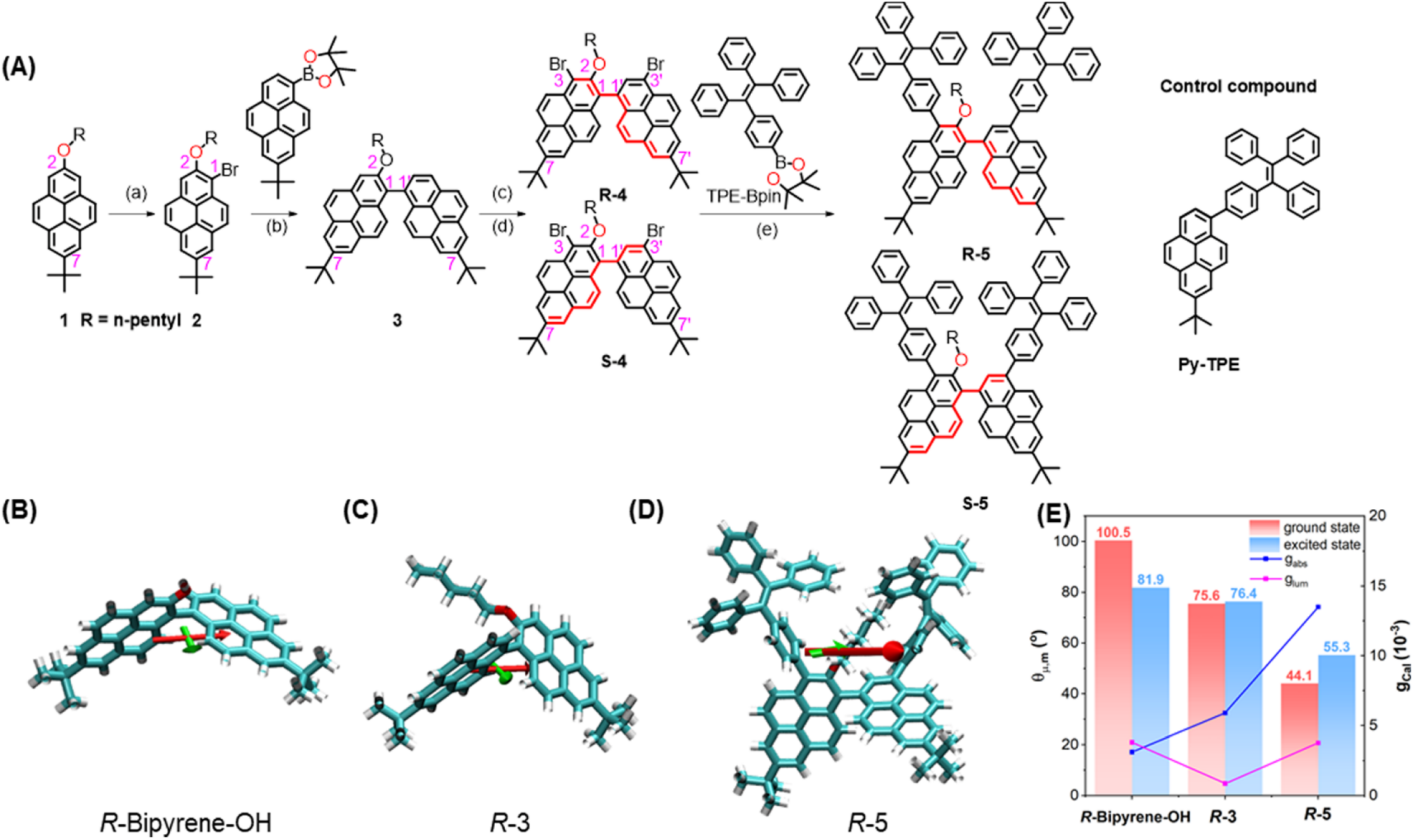
Molecular structure and simulation. (A) Synthetic route for the new intrinsically chiral bipyrenyl-based molecules *R-*5 and *S-*5, and the molecular structure of the control compound Py-TPE. (a) NBS, CH_2_Cl_2_, (b) Pd(PPh_3_)_4_, K_2_CO_3_, toluene/ethanol/H_2_O, (c) BTMABr_3_, CH_2_Cl_2_, and (d) chiral resolution (chiral HPLC columns, CH_3_CN); (B–D) the optimized molecular structure and (E) calculated *µ*_e_, *µ*_m_, and *g*_cal_ for the S_1_ → S_0_ transition for bipyrene-OH (detailed molecular structure in Scheme S1), *R-*3 and *R-*5.

The detailed synthetic route for the target enantiomers *R-/S-*5 is presented in Fig. 1. The alkylation reaction between 7-*tert*-butyl-2-hydroxypyrene (Py-OH)^43,44^ and 1-bromopentane affords 1 in a high yield (93%). A subsequent Suzuki–Miyaura coupling reaction and bromination afforded the racemic chemical intermediates 4 (42% yield in 2 steps), which were further resolved using chiral high-performance liquid chromatography (HPLC) techniques to yield enantiomers *R-/S-*4 (Fig. S21). The ^1^H NMR spectrum of *R-/S-*4 is almost the same with one singlet peak at *δ* = 8.39 ppm (proton peak at 2′-position of pyrene), doublets at *δ* = 8.34, 8.32, 8.22 and 8.20 ppm with *J* values in the range of 1.4–1.5 Hz for the corresponding protons at 6-, 6’–, 8- and 8′-positions of the bipyrenyl core, and eight groups of doublets at *δ* = 8.60, 8.56, 8.28, 8.26,7.88, 7.84, 7.47 and 7.39 ppm for the protons with an integration ratio of 1 : 1:1 : 1:1 : 1 in the K-region (4-, 5-, 9- and 10-positions) of pyrene.

High-resolution mass spectrometry (HRMS) revealed a molecular peak at [*m*/*z*+H]^+^ of 759.1655; both results are consistent with pure enantiomers *R-/S-*4. Finally, a Suzuki–Miyaura coupling reaction between enantiomers *R-/S-*4 and 1-(4-phenylboronic acid pinacol ester)-1,2,2-triphenylethene (TPE-Bpin) affords enantiomers *R-*5 and *S-*5 in 30% and 37% yield, respectively. For comparison, 7-(*tert*-butyl)-1-(4-(1,2,2-triphenylvinyl)phenyl)pyrene (Py-TPE) was synthesized *via* a classical Suzuki–Miyaura coupling reaction, and the molecular structure is illustrated in Scheme S1. The molecular structures were fully characterized by ^1^H/^13^C NMR spectroscopy and HRMS (Fig. S1–S20). All compounds *R-/S-*4 and *R-/S-*5 show good solubility (>30 mg mL^−1^ in CH_2_Cl_2_) in common solvents (such as dichloromethane, tetrahydrofuran (THF), dimethyl sulfoxide (DMSO), *etc*).

### Theoretical calculations

The absolute molecular geometries of *R-*/*S-*5 and Py-TPE in the ground state were optimized using density functional theory (DFT) at the CAM-B3LYP/6-311G(d,p) level.^45^ As shown in Fig. 1B–D, S22 and S23, both pyrene rings adopt a non-planar conformation with a dihedral angle of 68.25° and −68.25° for *R-*5 and *S-*5, respectively. Moreover, the corresponding *R-*5 and *S-*5 show a clear bisignate Cotton effect with opposite signs in the 250–450 nm region. The computational results reflect the chiral relationship between *R-*5 and *S-*5 (Fig. S25). Thus, these bipyrenyl-based molecules are novel chiral compounds, and the key chiral source originates from the intrinsically chiral bipyrenyl skeleton. The key parameters of |*µ*|, |*m*|, and vector angle *θ*_*µ*,*m*_ value for 3 and *R-*5 and *S-*5 were estimated by time-dependent DFT calculations (CAM-B3LYP/6-311G(d,p)), and the calculated parameters are summarized in Tables S1 and S2. At the single-molecular level, the calculated vector angle *θ*_*µ*,*m*_ decreased from 100.5° to 44.1° in the ground state and decreased from 81.9^o^ to 55.3^o^ in the excited state following the order of *R-/S-*bipyrene-OH > *R/S-*3 > *R/S-*5 (Fig. 1E). According to eqn (1), the calculated g_abs_ value increased from 3.12 × 10^−3^ to 1.35 × 10^−2^ in the ground state, suggesting that the substituents at the 2-position and the 3,3′-positions of pyrene could enhance the chiroptical activity. The calculated *g*_lum_ value increased from 8.70 × 10^−4^ (*R/S-*3) and 3.82 × 10^−3^ (*R/S-*bipyrene-OH) to 3.77 × 10^−2^ (*R-*5), indicating that the flexible alkyl chain at the 2-position of pyrene could promote molecular flexibility, resulting in a lowering of the *g*_lum_ value,^46^ while the substituents at the 3,3′-positions are beneficial for improving the *g*_lum_ value. In addition, the dihedral angle between the two pyrenes is 53.95° in the excited state, which is smaller than that in the ground state. These results indicate that the changes in molecular conformations in the two states can lead to a differential chirality dynamic process. Additionally, although the helical configuration of the tetraphenylethylene (TPE) moiety can contribute to chirality delivery through intramolecular cyclization and dynamic covalent bonds^40,47,48^ the four phenyl rings of the TPE unit can rotate freely with a low energy barrier (∼0.5 kcal mol^−1^) in the present system (Fig. S28). As a result, its influence on chirality delivery is limited in these intrinsically chiral bipyrenyl-based AIEgen systems.

Furthermore, due to the steric influence of the alkyl group at the 2-position, the dihedral angle is 80.7° between the phenyl ring of the TPE unit at the 3-position and the pyrene ring, which is larger than that for the other TPE unit at the 3′-position with the pyrene ring (53.5°). The twist conformation could weaken electronic communication, resulting in the HOMO and LUMO being primarily located on the two pyrene rings and a section of the TPE unit at the 3′-position (Fig. S26 and Table S3).

### Optical properties

**Fig. 2:**
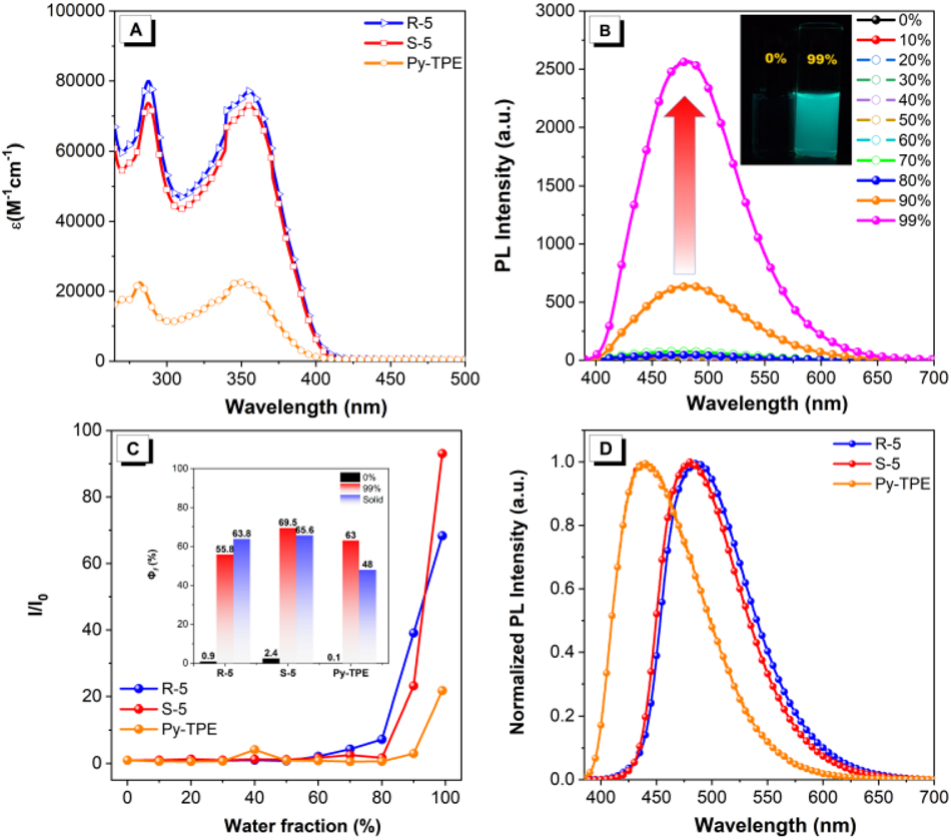
Photophysical property analysis. (A) Absorption spectra; (B) emission spectra of compound *S-*5 in THF and THF/H_2_O mixture with various water fractions (*f*_w_) (10 µM). Inset: fluorescence images of compound *S-*5 in *f*_w_ = 0% and 99% under UV irradiation (*λ*_ex_ = 365 nm); (C) plot of relative PL intensity *I*/*I*_0_ at different water fractions for *R*-/*S*-5. Inset: quantum yields of compounds *R-/S-*5 in *f*_w_ = 0% (black), 99% (red), and in the thin film (blue); (D) emission spectra of compounds *R-/S-*5 in the solid state.

The UV-vis absorption spectra of enantiomers *R-/S-*5 and Py-TPE were measured in dilute THF solution (10^−5^ M). As shown in Fig. 2A, these compounds exhibit similar well-resolved absorption bands in the region of 270–420 nm with a large molar absorption coefficient (*ε*) of up to 10^4^ M cm^−1^. The short-wavelength absorption and long-wavelength absorption bands of *R-/S-*5 are located at ∼290 nm and ∼356 nm, respectively. The observed absorption band at 356 nm is assigned to the overlapped S_0_ → S_1_ transition (*f* = 0.7204 for *R-*5, and 0.7318 for *S-*5) and S_0_ → S_2_ transition (*f* = 0.8698 for *R-*5, and 0.8283 for *S-*5) (Table S3).^43^ Evidently, the *ε* value of the long-wavelength absorption band is almost identical for both *R-*5 (7.93 × 10^4^ M^−1^ cm^−1^) and *S-*5 (7.29 × 10^4^ M^−1^ cm^−1^). Comparatively, compound Py-TPE exhibits a slightly blue-shifted absorption with the maximum band at 348 nm and a decreased *ε* value of 2.27 × 10^4^ M^−1^ cm^−1^. This may be attributed to the substituent effect of only one TPE unit attached to the 1-position of the pyrene core.^49^

Upon excitation, both compounds *R-*5 and *S-*5 containing two TPE units displayed similar emission behaviour in pure THF and the THF/H_2_O mixture with different water fractions (*f*_w_) (∼10^−5^ M) (Fig. 2B and S32); the results are summarized in Table 1. For example, *S-*5 emits a weak blue emission with a maximum emission peak at 433 nm and a *Φ*_f_ of 0.02 in THF solution. The fluorescence intensity remains very weak when the water fraction (*f*_w_) is less than 80%. Subsequently, the fluorescence intensity rapidly increased 5-fold with a maximum emission peak red-shifted to 482 nm upon increasing the *f*_w_ from 80 to 99%, with an enhanced *Φ*_f_ of 0.68. Meanwhile, *R-*5 also exhibits enhanced emission intensity with increased *Φ*_f_ from 0.01 to 0.64 as the *f*_w_ increased to 99%. Thus, both *R-*5 and *S-*5 exhibit clear AIE characteristics (Fig. 2B, C and S32). Meanwhile, TPE-decorated Py-TPE exhibits the characteristic AIE effect, and is non-emissive in THF solution with an extremely low *Φ*_f_ (less than 0.01), but the emission intensity increases approximately 22-fold with a *Φ*_f_ value of up to 0.63 (Tables 1 and S33). Additionally, the enantiomers *R-/S-*5 emit bright blue emission at 487 and 481 nm with slightly decreased *Φ*_f_ values in the range of 0.55–0.63 in the solid state (Fig. 2D). The maximum emission peak of Py-TPE is located at 437 nm. The *Φ*_f_ value decreased to 0.48 compared to its value in *f*_w_ = 99%, which is ascribed to the stronger molecular aggregation in the solid state. Furthermore, the calculated energy barrier of *R-*5 is slightly lower than that of *S-*5, indicating that the *R-*5 configuration possesses greater thermodynamic stability, which is consistent with its relatively high *Φ*_f_ (Fig. S27). Additionally, the photoluminescence (PL) behaviour of these TPE-decorated pyrenes showed no clear dependence on solvent polarity (Fig. S29–S31).

**Table 1:** Summary of photophysical properties of compounds *R-/S-*5

Compd.	*λ*_maxabs_ (*ε*_max_)^a^ (M^−1^ cm^−1^)	*λ*_maxPL_ (nm)	*τ* (ns)	*Φ*_f_	*α*_AIE_ d	*k*_r_ e (10^7^ s^−1^)	*k*_nr_ f (10^7^ s^−1^)	FWHM (nm)
*R*-5	287 (8.20 × 10^4^)	432^a^	1.32^a^	0.01^a^	64	0.76^a^	75.00^a^	68^a^
		487^b^	3.35^b^	0.64^b^		19.10^b^	10.70^b^	103^b^
	355 (7.93 × 10^4^)	487^c^	2.77^c^	0.60^c^		21.70^c^	14.40^c^	88^c^
*S*-5	290 (7.16 × 10^4^)	433^a^	1.32^a^	0.02^a^	33	1.52^a^	74.25^a^	77^a^
		482^b^	3.42^b^	0.68^b^		19.90^b^	9.36^b^	103^b^
	356 (7.29 × 10^4^)	481^c^	2.67^c^	0.56^c^		21.10^c^	16.5^c^	85^c^
Py-TPE	281 (2.23 × 10^4^)	400^a^	3.20^a^	0.0003^a^	2100	0.01^a^	31.24^a^	61^a^
		490^b^	3.34^b^	0.63^b^		18.86^b^	11.08^b^	87^b^
	348 (2.27 × 10^4^)	437^c^	2.48^c^	0.48^c^		19.35^c^	20.97^c^	86^c^

a Measured in THF solution.

b Measured in a THF/H_2_O mixture with *f*_w_ = 99%.

c Measured in a thin film.

d *α* = *Φ*_film_/*Φ*_soln_.

e *k*_r_ = radiative decay rate (*Φ*/*τ*).

f *k*_nr_ = nonradiative decay rate (1/*τ* − *k*_r_).

Time-resolved photoluminescence (TRPL) experiments were performed (Fig. S34). The enantiomers *R-/S-*5 and Py-TPE exhibit single exponential fluorescence decay with similar fluorescence quantum lifetime (*τ*) in the range of 1.32–3.42 ns in both THF solution and the solid state. On the other hand, the radiative decay rate (*k*_r_) and non-radiative decay (*k*_nr_) of compounds *R-*5, *S-*5 and Py-TPE were calculated following the equations *k*_r_ = *Φ*_f_/*τ* and *Φ*_f_ = *k*_r_/(*k*_r_ + *k*_nr_), where *Φ*_f_ and *τ* are the fluorescence quantum yield (*Φ*_f_) and fluorescence lifetime, respectively. The calculated *k*_r_ increased from 0.76 ×10^7^ to 17.61 ×10^7^ for *R*-5, 1.51 × 10^7^ to 10.46 ×10^7^ for *S-*5, and 0.01 × 10^7^ to 19.35 × 10^7^ for Py-TPE, while *k*_nr_ decreased from 75.00 × 10^7^ to 12.24 × 10^7^ for *R-*5, 74.25 × 10^7^ to 18.78 × 10^7^ for *S-*5 and 31.24 × 107 to 20.97 × 10^7^ for Py-TPE, as the *f*_w_ increased from 0 to 99%, respectively. Thus, these TPE-decorated enantiomers *R-/S-*5 and Py-TPE are indeed typical AIE-active molecules with high solid-state emission behaviour. More importantly, the presence of a large number of TPE units is beneficial for enhancing the *Φ*_f_ value in the solid-state.

### CD spectra, CPL spectra and morphology analysis

**Fig. 3:**
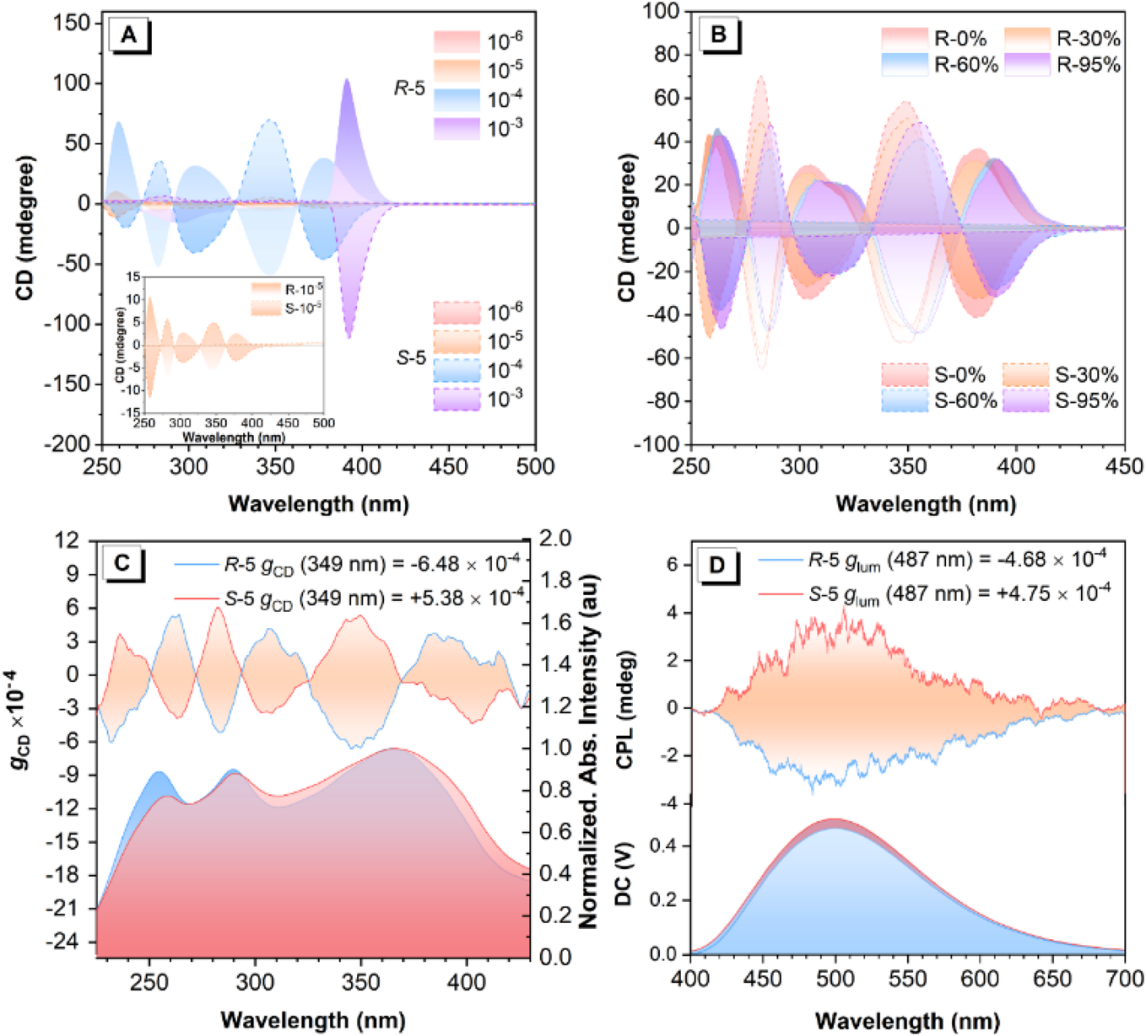
Chiroptical properties. (A) Concentration-dependent CD spectra of *R-/S-*5 in THF solution (10^−6^∼10^−3^ M); (B) CD spectra of compounds *R-/S-*5 in THF and in THF/H_2_O mixture with water fractions (*f*_w_) = 0%, 30%, 60%, and 95% (10 µM), respectively; (C) CD spectra and *g*_CD_ factors and (D) CPL spectra, *g*_lum_ factors and PL spectra of *R-/S-*5 in a KBr pellet at one rotation angle.

Circular dichroism (CD) spectroscopy provides a reliable technique for identifying the differential absorption of left- and right-handed circularly polarized light by chiral molecules. As shown in Fig. 3A, both *R-/S-*5 in THF solution (∼10^−5^ M) exhibit an almost silent CD signal in the absorption band range of 200–450 nm with a concentration of ∼10^−7^ M (Fig. 3A and S35). However, as the concentration increased from ∼10^−7^ to 10^−4^ M, both *R-*5 and *S-*5 gradually exhibited enhanced mirror-image CD signals with opposite Cotton effects in the range from 250 nm to 500 nm, respectively, with an enhanced absorption dissymmetry factor (|*g*_CD_|) value of 4.73 × 10^−5^ (10^−6^ M) to 4.12 × 10^−3^ (10^−4^ M) (378 nm). More importantly, when the concentration increases from 10^−4^ to 10^−3^ M, stronger mirror-image, positive and negative CD peaks merged at 391 nm for both compounds with a |*g*_CD_| value of 7.34 × 10^−3^. These observations suggest that these bipyrenyl-based architectures are chiral molecules, which undergo a concentration-induced CD effect with an enhanced *g*_CD_ value, indicating that chirality amplification occurs from the mono-axisymmetric enantiomers to aggregated enantiomers *via* the formation of merged ordered self-assembled chiral architectures (Fig. S36).

Furthermore, the CD spectra were further measured in THF and a mixed THF/H_2_O solution with different water fractions (*f*_w_) (∼10^−5^ M). As illustrated in Fig. 2B and S37, compounds *R-*5 and *S*-5 show a clear aggregation-induced CD (AICD) effect as the *f*_w_ increased from 0 to 99%, with three AICD peaks originally at 257 nm, 303 nm, and 379 nm in THF gradually red-shifted to 266 nm, 316 nm, and 393 nm, respectively, accompanied by a slightly decreased intensity of the CD signal. This indicates that the self-assembled molecules in the aggregated state were capable of inducing chirality amplification. Therefore, we attribute the difference in *g*_CD_ values between the theoretical calculations and the experimental measurements to molecular aggregation behaviour. The theoretical model is based on isolated molecules in the gas phase, while the experimental measurements reflect the behaviour of the molecules in an aggregated state. It is therefore reasonable to observe differences between the two sets of values, as the molecular environment and intermolecular interactions differ substantially.

Fig. 3C displays a mirror-image Cotton effect with a maximum |*g*_CD_| value of up to 6.48 × 10^−4^ in the solid state. This effect was further verified by multiple angle-resolved measurements around the optical axis (Fig. S38). Although no clear CPL signals were observed for either chiral AIEgen *R-*5 or *S-*5 in THF solution, both *R-*5 and *S-*5 exhibited distinct CPL characteristics with the mirror-image peaks at 487 nm in the aggregated state (Fig. 3D and S39). As calculated using eqn (1), these chiral AIEgens exhibit |*g*_lum_| values of 4.68 × 10^−4^–4.75 × 10^−4^. This observation strongly suggests that the molecular chirality of compounds *R-*5 and *S-*5 can be effectively translated into measurable CPL signals through self-assembly processes occurring in the aggregated state. Compared to previous work (Table S4), the intrinsically chiral bipyrenyl-based *R-5* and *S-5* show higher *Φ*_f_ (0.66) and enhanced |*g*_lum_| values in the solid state *versus* TPE-decorated BINOL-based enantiomers.^50,51^

The morphology of the self-assembled nanostructures in different states was further investigated by SEM (Fig. S38–S40 and S41). As the concentration increased from 10^−5^ to 10^−3^ M, the morphologies changed from uniform block solids to micelles and then to vesicles with a clear shape for both *R-*5 and *S-*5. Meanwhile, as *f*_w_ increased from 0 to 99%, the shape of the block solids of *R-*5 in THF (10^−5^ M) also changed to helical linear-shape self-assembled fibers with increased average diameters (d) from *ca*. 1 µm to 10 µm. Moreover, the minimum units of the linear-shape self-assembled aggregates were uniform, hollow vesicles with diameters of *ca*. 700–900 nm. Similarly, the morphologies of *S-*5 changed from a colloidal state to hollow vesicles (*d* ≈ 500 nm) as *f*_w_ increased from 0 to 60%, which further led to the formation of helical linear-shape self-assembled fibers when *f*_w_ = 99%. We speculate that the hydrophilic, chiral bipyrenyl-based AIEgens prefer to self-assemble into hollow vesicles, which then evolve into opposite helical fibers during the aggregation process. The process corresponds to the enhanced mirror CD signals as the concentration of the two enantiomers increases. Moreover, due to the strengthening of the intermolecular interaction in the aggregated state, the CD signals show slightly red-shifted CD spectra.^52^ Moreover, the spontaneous formation of helical fibers through self-assembly generates the CPL signal in the aggregated state.

### Femtosecond transient absorption spectroscopy

**Fig. 4:**
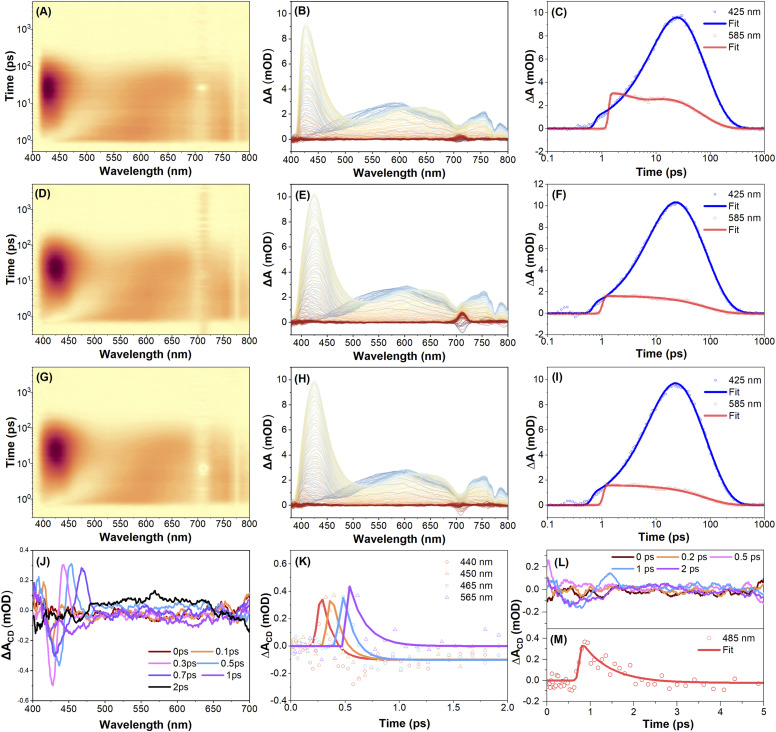
Femtosecond transient absorption spectroscopy analysis. (A, D, and G) Pseudo colour 2D plots of fs-TA data; (B, E, and H) fs-TA spectra at various time delays under 350 nm excitation and TA traces (C, F, and I) at 425 nm and 585 nm probe wavelengths of *S-*5 in THF solution. Both the pump and probe beams are linearly polarized for (A, B, and C). The pump is left circularly polarized (LCP), while the probe is set to be LCP for (D, E, and F), and right circularly polarized (RCP) for (G, H, and I), respectively; (J) spectral evolution and (K) transient dynamics for differential TA spectra of *S-*5; (L) spectral evolution and (M) transient dynamics for differential TA spectra of *R-*5.

To investigate the excited-state dynamics of the enantiomers *R-*5 and *S-*5, femtosecond transient absorption (fs-TA) spectra were recorded in THF solution with linear polarization at the magic angle configuration under 350 nm excitation. Full experimental details, including polarization modulation protocols and spectral acquisition parameters, are summarized in the Experimental methods section. Fig. 4A–C illustrates the fs-TA response of compound *S-*5. Immediately after excitation, a broad positive excited-state absorption (ESA) band appears at around 585 nm, which is attributed to the absorption from the upper excited states S_*n*_ → S_m_ (where *m* > *n*).^49,53,54^ This ESA band rapidly decays and red-shifts toward ∼650 nm, while a new band grows in at ∼424 nm within 11 ps. This is attributed to internal conversion (IC) from S_*n*_ to S_1_, followed by S_1_→ S_m_ ESA. Subsequently, the ESA bands at 425, 585, and 750 nm decay exponentially within ∼78 ps, ∼68 ps, and ∼66 ps, respectively, without further spectral evolution (Table S6). The TA spectra of *R-*5 under identical conditions are essentially the same as *S-*5's (Fig. 4L, M, S42 and Table S7), indicating comparable relaxation dynamics for both enantiomers under linear polarization excitation conditions.

To further explore the chiral-dependent excited-state dynamics, circularly polarized femtosecond transient absorption (CP-fs-TA) spectra of enantiomers *R-*5 and *S-*5 were recorded in THF solvent under 350 nm excitation.^55^ The CP-fs-TA setup employs circularly polarized pump and probe pulses generated *via* the combination of linear polarizers and quarter-wave plates. The pump beam is set to be left circularly polarized (LCP), while probe pulses alternate between LCP and right-circular polarization (RCP). No obvious difference was observed in compound *S-*5 in the CP- fs-TA spectra under LCP (Fig. 4D–F) or RCP (Fig. 4G–I) light at 350 nm excitation. The chiral-correlated spectral features were extracted according to the differential TA spectra: Δ*A*(CD) = Δ*A*(LCP) − Δ*A*(RCP). The kinetic curves and fitting results for *S*-5 are summarized in Fig. 4J–K and Table S7. A negative band at 425 nm and a positive band at 440 nm immediately appear after excitation and rapidly decay within 0.3 ps. Accordingly, this differential shape signal gradually red-shifted to 450 nm and 446 nm after a 0.7 ps delay; the red-shifted band then diminishes, accompanied by the emergence of a new positive band at 565 nm within 2 ps. This indicates that circularly polarized light excitation induces chirality-sensitive changes in the excited-state population, which may originate from transient conformational changes (*e.g.*, chiral structural relaxation in the excited states). This result was supported by theoretical calculations, where the torsion angle between the two pyrene rings is different for the S_0_ and S_1_ states under the optimized molecular conformation conditions (Fig. S22 and S24). However, for *R-*5, the differential spectroscopy did not show clear changes in this spectral region until 1 ps; a clear negative band and positive band at 450 nm and 485 nm were observed. Thus, these results reflect that both intrinsically chiral compounds *R-*5 and *S-*5 exhibit a fast dynamic process *via* excitation-induced chirality conformation changes in solution within a less than 2 ps delay.

**Fig. 5:**
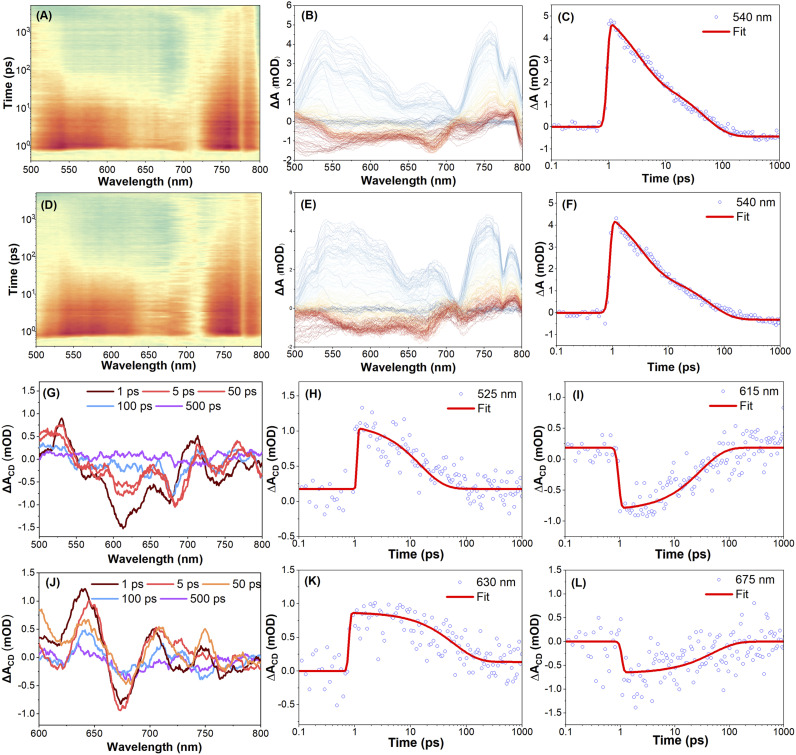
Femtosecond transient absorption spectroscopy analysis. (A and D) Pseudo colour 2D plots of TA data under 350 nm excitation; (B and E) fs-TA spectra at various time delays and fs-TA traces (C and F) at a 600 nm probe wavelength of *S-*5 in the solid state. The pump is left circularly polarized (LCP), while the probe is set to be LCP for (A, B, and C), and right circularly polarized (RCP) for (D, E, and F), respectively; (G) spectral evolution and the transient dynamics for differential TA spectra (H) at 525 nm and (I) at 615 nm probe wavelengths of *S-*5 in the film state; (J) spectral evolution and the transient dynamics for differential TA spectra (K) at 630 nm and (L) at 675 nm probe wavelengths of *R-*5 in the film state.

To further investigate the solid-state chiral photophysics, the CP-fs-TA spectra of compounds *R-*5 and *S-*5 in thin films were examined under LCP light excitation at *λ*_ex_ = 350 nm (Fig. 5 and Table S8). Due to intense fluorescence interference from the solid-state emission, TA spectra data were collected in the range 500–800 nm. For *S-*5, the initial spectra of *S-*5 show excited state absorption (ESA) bands at 540 nm and 750 nm under an LCP light excitation (Fig. 5A–C). The intensity of the TA spectra was enhanced within 3 ps delay time, which is assigned to the S_*n*_ → S_m_ transition (where *m* > *n*) and is attributed to the conformational relaxation. Meanwhile, under RCP pump excitation at *λ*_ex_ = 350 nm, *S-*5 exhibits similar TA absorption spectra to those observed for *R-*5 during the whole delay time process (Fig. 5D–F). There was no obvious difference in the fs-TA spectra of compound *R-*5 under LCP or RCP probe *versusS-*5 (Fig. S43). However, the differential spectroscopy of compounds *R-*5 and *S-*5 shows a non-zero differential transient absorption Δ*A*(CD) signal with mirror-symmetry in the range of 500–800 nm under the same experimental conditions, indicating that *R-*5 and *S-*5 are enantiomorphs. As shown in Fig. 5G–I, the differential spectrum exhibits two featuring bands at 525 and 713 nm, and a negative band at 615 nm for *S-*5 after 1 ps delay, while the differential spectrum of *R-*5 shows a positive band at 630 nm and a negative band at 675 nm (Fig. 5J–L). Furthermore, the differential spectra decay with a time constant of ∼500 ps for both *S-*5 and *R-*5, which is a hundred-fold order of magnitude longer compared to that of the solution (less than 2 ps). The distinctive CP-fs-TA spectra provide compelling evidence to elucidate why both enantiomers *S-*5 and *R-*5 did not show a CPL signal in solution, but exhibit a profound mirror CPL signal in the film. This behaviour is attributed to a dynamic chirality reconfiguration that occurs in the solid state, resulting in a longer excited state chirality delay time.^42^

#### Experimental methods

##### General methods

^1^H and ^13^C NMR spectra (400 MHz) were recorded on a Bruker AV 400M spectrometer using chloroform-d or DMSO-d_6_ as the solvent and tetramethylsilane as the internal reference. *J*-values are given in Hz. High-resolution mass spectra (HRMS) were recorded on an LC/MS/MS system, which consisted of an HPLC system (Ultimate 3000 RSLC, Thermo Scientific, USA) and a Q Exactive Orbitrap mass spectrometer. High performance liquid chromatography was performed on a Waters UPCC analytical SFC using the SFC analytical method (column: Daicel ChiralPak IH, 3 mm I.D.×100 mm, 3 µm; mobile phase: A for CO_2_ and B for MeOH; gradient: B 35% in 7 min; flow rate: 2.0 mL min^−1^; column temperature: 35 °C; wavelength: 210 nm). UV-vis absorption spectra and photoluminescence (PL) spectra were recorded on a Shimadzu UV-2600 and a Hitachi F-4700 spectrofluorometer. Circular dichroism (CD) spectra were obtained by using a JASCO J-815 spectrometer. The solid-state samples for the CD spectra were prepared using the KBr pellet method, and the samples were rotated after each measurement to eliminate chiroptical artifacts. The solid-state samples for CPL spectra were also prepared using the KBr pellet method. The method for measuring the CPL of the solid samples in a KBr pellet from different angles is as previously reported.^3^ The CPL spectra of KBr pellets were measured using a JASCO CPL-300 spectrometer and an internal xenon lamp as the excitation light source with ‘standard’ sensitivity, at 500 nm min^−1^ scan speed and a response time of 1.0 s, an excitation wavelength of 340 nm, and the slit widths of 3000 µm for Ex and 3000 µm for Em, respectively. CPL spectra were recorded with at least 8 times accumulation in a KBr pellet. The PL quantum yields were measured using an integrating sphere on an Edinburgh FLS 980 instrument. The fluorescence lifetime was recorded on an Edinburgh FLS 980 instrument and measured using a time-correlated single-photon counting method. The quantum chemistry calculations were performed using the Gaussian 16 (CAM-B3LYP/6–31G (d,p) basis set) software package.

### Femtosecond transient absorption spectroscopy measurement

The transient absorption (TA) spectra were recorded using an amplified Ti laser system (Solstice Ace, Spectra Physics), which delivers 800 nm pulses at a 1 kHz repetition rate, with a pulse duration of 35 fs and a power output of 5.6 W. Twenty percent of the 800 nm output (1.12 W) was directed to an optical parametric amplifier (TOPAS-Prime, Newport) to generate 370 nm pump pulses. Sixty-four percent of the 800 nm output (3.584 W) was converted to its second harmonic at 400 nm using a 2 mm barium borate (BBO) crystal. A small fraction of the 800 nm fundamental beam (500 nJ per pulse) was focused onto a 10 mm cuvette containing distilled water to generate the white-light continuum (WLC) probe pulses. The probe pulse polarization was set to the magic angle relative to the excitation pulse. A computer-controlled, piezo-driven high-precision translation stage (Physik Instrumente), combined with a long-travel motorized stage (Newport), was positioned in the pump beam path to create a time delay between the pump and probe pulses, with a time delay precision of 1 fs and a time window of 5 ns. All measurements were performed under ambient conditions at room temperature.

#### Synthesis of 7-*tert*-butyl-2-pentyloxypyrene (1)

Under a nitrogen atmosphere, 7-*tert*-butyl-2-hydroxylpyrene (Py-OH)^34,35^ (3.0 g, 10.9 mmol, 1.0 eq.), *n*-amyl iodide (5.4 g, 27.2 mmol, 2.5 eq.) and Cs_2_CO_3_ (2.0 g, 55.5 mmol, 1.0 eq.) were added into a round-bottomed flask containing a solution of *N*,*N*-dimethylformamide (12 mL). The mixture was stirred at 130 °C for 24 h. After cooling, the mixed solution was quenched with H_2_O and extracted with DCM (50 mL × 3), and the organic layer was washed with water and brine. The combined organic layer was dried with anhydrous MgSO_4_ and then evaporated. The crude product was further purified by silica gel column chromatography using *n*-hexane as the eluent to obtain a light white solid powder 2 (3.5 g, 93%). ^1^H NMR (400 MHz, CDCl_3_) *δ* 8.18 (s, 2H), 8.02 (d, *J* = 9.0 Hz, 2H), 7.95 (d, *J* = 9.0 Hz, 2H), 7.68 (s, 2H), 4.25 (t, *J* = 6.6 Hz, 2H), 2.02–1.81 (m, 2H), 1.60–1.53 (m, 2H), 1.57 (s, 9H), 1.52–1.43 (m, 2H), and 0.98 (t, *J* = 7.2 Hz, 3H) ppm. ^13^C NMR (100 MHz, CDCl_3_) *δ* 157.2, 156.7, 148.0, 132.4, 131.6, 130.0, 128.2, 127.5, 126.7, 123.0, 122.5, 120.0, 111.3, 110.8, 68.5, 35.2, 32.0, 29.7, 29.2, 28.4, 22.6, and 14.1 ppm.

#### Synthesis of 7-*tert*-butyl-1-bromo-2-pentyloxypyrene (2)

Under a nitrogen atmosphere, 7-*tert*-butyl-2-pentyloxypyrene (1) (1.0 g, 2.9 mmol, 1.0 eq.) and *N*-bromosuccinimide (NBS) (517 mg, 2.9 mmol, 1.0 eq.) were added into a round-bottom flask containing dry DCM (20 mL), and the mixture was stirred at room temperature for 24 h. After reaction, the mixed solution was quenched with H_2_O and extracted with DCM (50 mL×3), and the combined organic layer was washed with water and brine. The combined organic layer was dried with anhydrous MgSO_4_ and then evaporated. The crude product was further purified by silica gel column chromatography using *n*-hexane as the eluent to obtain a light white powder 7-*tert*-butyl-1-bromo-2-pentyloxypyrene (2) (710 mg, 59%). ^1^H NMR (400 MHz, CDCl_3_) *δ* 8.44 (d, *J* = 9.3 Hz, 1H), 8.22 (d, *J* = 2.7 Hz, 2H), 8.13 (d, *J* = 9.3 Hz, 1H), 8.06 (d, *J* = 9.0 Hz, 1H), 7.95 (d, *J* = 8.9 Hz, 1H), 7.68 (s, 1H), 4.32 (t, *J* = 6.5 Hz, 2H), 2.01 (dq, *J* = 9.5, 6.6 Hz, 2H), 1.63 (dd, *J* = 7.9, 2.9 Hz, 2H), 1.58 (s, 9H), 1.51–1.41 (m, 2H), and 0.99 (t, *J* = 7.3 Hz, 3H) ppm. ^13^C NMR (100 MHz, CDCl_3_) *δ* 153.4, 148.6, 131.2, 131.1, 130.1, 129.9, 129.7, 128.3, 126.5, 125.7, 123.1, 123.0, 122.2, 120.8, 110.2, 109.0, 69.9, 35.2, 31.9, 29.1, 28.4, 22.5, and 14.1 ppm.

#### Synthesis of racemic 7,7′-di-*tert*-butyl-2-pentyloxy-1,1′-bipyrene (3)

Under a nitrogen atmosphere, 7-*tert*-butyl-1-bromo-2-pentyloxypyrene (540 mg, 1.28 mmol, 1.0 eq.), 1-bpin-7-*tert*-butylpyrene (540 mg, 1.41 mmol, 1.1 eq.) and K_2_CO_3_ (530 mg, 3.84 mmol, 3.0 eq.) were added into a round bottomed flask containing a mixed solution of toluene, ethanol and water (14 mL) (*V*_toluene_ : *V*_ethanol_ : *V*_water_ = 5 : 1 : 1). The mixture was stirred for 5 min and then Pd(PPh_3_)_4_ (100 mg, 0.08 mmol) was added. The mixture was stirred at 90 °C for 24 h. After cooling, the mixed solution was quenched with H_2_O and extracted with DCM (50 mL × 3), and the organic layer was washed with water and brine. The combined organic layer was dried with anhydrous MgSO_4_ and then evaporated. The crude product was further purified by silica gel column chromatography using *n*-hexane as the eluent to obtain a light-yellow solid powder racemic 7,7′-di-*tert*-butyl-2-pentyloxy-1,1′-bipyrene (3) (540 mg, 70%). ^1^H NMR (400 MHz, CDCl_3_) *δ* 8.31 (d, *J* = 7.8 Hz, 1H), 8.26 (d, *J* = 1.9 Hz, 1H), 8.24 (d, *J* = 1.8 Hz, 1H), 8.18 (d, *J* = 9.0 Hz, 1H), 8.16–8.14 (m, 2H), 8.12 (d, *J* = 2.3 Hz, 3H), 8.03 (d, *J* = 7.7 Hz, 1H), 7.90 (s, 1H), 7.81 (d, *J* = 9.2 Hz, 1H), 7.76 (d, *J* = 9.3 Hz, 1H), 7.50 (d, *J* = 9.2 Hz, 1H), 7.37 (d, *J* = 9.3 Hz, 1H), 4.23–4.06 (m, 2H), 1.58 (s, 9H), 1.56 (s, 9H), 1.52–1.39 (m, 4H), 0.90–0.83 (m, 2H), and 0.52 (t, *J* = 6.8 Hz, 3H) ppm. ^13^C NMR (100 MHz, CDCl_3_) *δ* 155.4, 149.1, 148.3, 132.5, 132.2, 131.8, 131.4, 131.1, 130.7, 130.5, 130.5, 130.1, 129.2, 128.3, 128.3, 128.2, 127.6, 127.5, 126.9, 126.1, 125.6, 125.6, 125.0, 124.5, 123.3, 123.1, 122.7, 122.6, 122.3, 122.3, 120.2, 109.1, 69.3, 35.3, 35.3, 32.1, 29.9, 28.88, 28.85, 28.1, 22.19, 22.16, 13.8, and 13.8 ppm. HRMS (FTMS + p APCI) *m*/*z*: [M + H]^+^ calcd for C_45_H_45_O 601.3470; found, 601.3469.

#### Synthesis of enantiomer 7,7′-di-*tert*-butyl-3,3′-dibromo-2-pentyloxy- 1,1′-bipyrene (*R*-/*S*-4)

Under a nitrogen atmosphere, 7,7′-di-*tert*-butyl-2-pentyloxy-1,1′-bipyrene (4) (540 mg, 0.9 mmol, 1.0 eq.) and *N*-bromosuccinimide (NBS) (369 mg, 2.07 mmol, 2.3 eq.) were added to dry DCM (20 mL), and the mixture was stirred at room temperature for 24 h. After the reaction, the mixed solution was quenched with H_2_O and extracted with DCM (50 × 3 mL), and the combined organic layer was washed with water and brine. The combined organic layer was dried with anhydrous MgSO_4_ and then evaporated. The crude product was further purified by silica gel column chromatography using *n*-hexane as eluent to obtain a light white powder racemic 7,7′-di-*tert*-butyl-3,3′-dibromo-2-pentyloxy-1,1′-bipyrene (4) (510 mg, 75%). The racemic 4 was purified using chiral high performance liquid chromatography (HPLC) techniques using CO_2_ and methanol (gradient: B 35% in 7 min, flow rate: 2.0 mL min^−1^) as mobile phases to achieve enantiomers 250 mg (retention time: 2.540 min) of *R-4* and 260 mg of *S-4* (retention time: 4.288 min) (weight ratio: *R-*4 : *S-*4 = 49.1 : 50.9) respectively. *R-*4:^1^H NMR (400 MHz, CDCl_3_) *δ* 8.60 (d, *J* = 9.3 Hz, 1H), 8.56 (d, *J* = 9.2 Hz, 1H), 8.39 (s, 1H), 8.34 (d, *J* = 1.6 Hz, 1H), 8.32 (d, *J* = 1.7 Hz, 1H), 8.28 (d, *J* = 5.3 Hz, 1H), 8.26 (d, *J* = 5.3 Hz, 1H), 8.22 (d, *J* = 1.6 Hz, 1H), 8.19 (d, *J* = 1.6 Hz, 1H), 7.87 (d, *J* = 9.2 Hz, 1H), 7.84 (d, *J* = 9.3 Hz, 1H), 7.47 (d, *J* = 9.2 Hz, 1H), 7.38 (d, *J* = 9.2 Hz, 1H), 3.91–3.76 (m, 1H), 3.54–3.49 (m, 1H), 1.59 (s, 9H), 1.58 (s, 9H), 1.30–1.23 (m, 1H), 1.21–1.09 (m, 1H), 0.77–0.62 (m, 3H), 0.46–0.40 (m, 1H), and 0.34 (t, *J* = 7.0 Hz, 3H) ppm. ^13^C NMR (125 MHz, CDCl_3_) *δ* 152.5, 150.1, 149.7, 133.1, 132.2, 131.3, 131.2, 131.0, 130.8, 130.7, 130.5, 130.0, 129.9, 129.6, 129.5, 129.2, 128.7, 128.4, 126.2, 126.1, 126.0, 125.4, 125.2, 123.5, 123.4, 123.4, 123.3, 122.9, 122.5, 122.3, 119.5, 116.2, 74.1, 35.4, 35.3, 32.0, 29.6, 27.7, 22.1, and 13.7 ppm. *S-*4: ^1^H NMR (400 MHz, CDCl_3_) *δ* 8.60 (d, *J* = 9.2 Hz, 1H), 8.56 (d, *J* = 9.2 Hz, 1H), 8.39 (s, 1H), 8.34 (d, *J* = 1.5 Hz, 1H), 8.32 (d, *J* = 1.4 Hz, 1H), 8.27 (d, *J* = 9.3 Hz, 1H), 8.26 (d, *J* = 9.3 Hz, 1H), 8.22 (d, *J* = 1.3 Hz, 1H), 8.19 (d, *J* = 1.4 Hz, 1H), 7.87 (d, *J* = 9.3 Hz, 1H), 7.83 (d, *J* = 9.3 Hz, 1H), 7.47 (d, *J* = 9.2 Hz, 1H), 7.39 (d, *J* = 9.2 Hz, 1H), 3.92–3.74 (m, 1H), 3.54–3.49 (m, 1H), 1.59 (s, 9H), 1.58 (s, 9H), 1.30–1.22 (m, 1H), 1.16–1.12 (m, 1H), 0.81–0.57 (m, 3H), 0.46–0.43 (m, 1H), and 0.34 (t, *J* = 7.0 Hz, 3H) ppm. ^13^C NMR (100 MHz, CDCl_3_) *δ* 152.5, 150.0, 149.6, 133.0, 132.2, 131.2, 131.2, 131.0, 130.7, 130.6, 130.5, 130.0, 129.8, 129.5, 129.4, 129.2, 128.6, 128.4, 126.1, 126.0, 125.9, 125.3, 125.1, 123.5, 123.4, 123.3, 123.2, 122.8, 122.5, 122.20, 119.4, 116.1, 76.7, 74.0, 68.0, 35.3, 35.3, 31.9, 29.5, 27.6, 25.6, 22.0, and 13.6 ppm. HRMS (FTMS + p APCI) *m*/*z*: [M + H]^+^ calcd for C_45_H_43_Br_2_O 757.1660; found, 759.1655.

#### Synthesis of enantiomers *R*-5 and *S*-5

Compounds *R-*5 and *S-*5 were synthesized by a Suzuki–Miyaura coupling reaction between *R-/S-*4 and 2-(7-(*tert*-butyl)pyren-1-yl)-4,4,5,5-tetramethyl-1,3,2-dioxaborolane with a considerable yield. The detailed synthetic procedures of *R-*5 and *S-*5 are illustrated below as an example.

#### Synthesis of *R*-5

Under a nitrogen atmosphere, 3,3′-dibromo-7,7′-di-*tert*-butyl-2-(pentyloxy)-1,1′-bipyrene (*R-*4) (155 mg, 0.2 mmol, 1.0 eq), 2-(7-(*tert*-butyl)pyren-1-yl)-4,4,5,5-tetramethyl-1,3,2-dioxaborolane (197 mg, 0.43 mmol, 2.1 eq.) and K_2_CO_3_ (113 mg, 0.82 mmol, 4.0 eq.) were added into a round bottomed flask containing a mixed solution of toluene, ethanol and water (7 mL) (*V*_toluene_ : *V*_ethanol_:*V*_water_ = 5 : 1 : 1). The mixture was stirred for 5 min and then Pd(PPh_3_)_4_ (100 mg, 0.08 mmol) was added. The mixture was stirred at 90 °C (oil bath) for 24 h. After cooling, the mixed solution was quenched with H_2_O and extracted with DCM (3 × 50 mL), and the organic layer was washed with water and brine. The combined organic layer was dried with anhydrous MgSO_4_ and then evaporated. The crude product was further purified by silica gel column chromatography using *n*-hexane as the eluent to obtain a light-yellow solid powder *R-5* (80 mg, 30%). *R-5*: ^1^H NMR (400 MHz, DMSO-d_6_) *δ* 8.37 (d, *J* = 8.7 Hz, 2H), 8.30 (s, 1H), 8.27 (s, 1H), 8.24 (d, *J* = 9.3 Hz, 1H), 8.18 (d, *J* = 9.4 Hz, 1H), 8.10 (d, *J* = 9.2 Hz, 1H), 7.98 (d, *J* = 9.4 Hz, 1H), 7.94 (d, *J* = 8.9 Hz, 2H), 7.73 (d, *J* = 9.3 Hz, 1H), 7.44–7.39 (m, 4H), 7.35 (d, *J* = 8.0 Hz, 2H), 7.25–6.96 (m, 34H), 3.26–3.11 (m, 1H), 3.11–2.98 (m, 1H), 1.54 (s, 9H), 1.52 (s, 9H), 0.98–0.90 (m, 1H), 0.88–0.80 (m, 3H), 0.77 (t, *J* = 3.7 Hz, 1H), 0.66–0.52 (m, 2H), and 0.42–0.27 (m, 2H) ppm. ^13^C NMR (100 MHz, CDCl_3_) *δ* 153.2, 149.2, 148.8, 143.9, 143.8, 143.8, 143.7, 142.8, 142.7, 141.3, 141.2, 141.0, 140.8, 139.1, 136.9, 135.1, 132.0, 131.5, 131.5, 131.5, 131.4, 131.4, 131.3, 131.2, 131.2, 131.1, 131.0, 130.9, 130.8, 130.6, 130.5, 130.1, 129.9, 129.7, 128.0, 127.9, 127.8, 127.8, 127.7, 127.7, 127.5, 126.6, 126.5, 125.9, 125.7, 125.3, 125.2, 124.8, 123.4, 123.0, 122.6, 122.5, 122.4, 122.2, 121.8, 73.6, 68.0, 35.2, 35.2, 31.9, 30.4, 29.7, 29.5, 27.4, 25.6, 22.0, and 13.5 ppm. HRMS (FTMS + p APCI) *m*/*z*: [M + Na]^+^ calcd for C_97_H_80_ONa 1284.6140; found, 1284.6127.

#### Synthesis of *S*-5

Light yellow solid powder *S*-5 (90 mg, 37%). ^1^H NMR (400 MHz, DMSO-d_6_) *δ* 8.37 (d, *J* = 8.8 Hz, 2H), 8.31 (s, 1H), 8.28 (s, 1H), 8.24 (d, *J* = 9.4 Hz, 1H), 8.19 (d, *J* = 9.4 Hz, 1H), 8.10 (d, *J* = 9.3 Hz, 1H), 8.00–7.93 (m, 3H), 7.74 (d, *J* = 9.3 Hz, 1H), 7.44 (d, *J* = 8.5 Hz, 2H), 7.40–7.34 (m, 4H), 7.24–6.94 (m, 34H), 3.22–3.13 (m, 1H), 3.10–2.98 (m, 1H), 1.54 (s, 9H), 1.52 (s, 9H), 0.98–0.90 (m, 1H), 0.85–0.76 (m, 4H), 0.61–0.59 (m, 2H), 0.37–0.31 (m, 2H), and 0.23–0.18 (m, 2H) ppm. ^13^C NMR (100 MHz, CDCl_3_) *δ* 152.2, 148.1, 147.8, 142.9, 142.8, 142.7, 142.7, 141.7, 141.6, 140.2, 140.17, 140.0, 139.7, 138.1, 135.8, 134.0, 131.0, 130.5, 130.4, 130.4, 130.3, 130.1, 130.0, 129.8, 129.7, 129.5, 129.1, 128.8, 128.6, 127.0, 126.9, 126.7, 126.7, 126.6, 126.5, 125.5, 125.5, 124.8, 124.6, 124.3, 124.1, 122.0, 121.6, 121.4, 121.3, 121.1, 120.8, 72.6, 67.0, 34.2, 34.1, 30.9, 28.5, 26.3, 25.9, 24.6, 20.9, and 12.5 ppm. HRMS (FTMS + p APCI) *m*/*z*: [M + Na]^+^ calcd for C_97_H_80_ONa 1284.6140; found, 1284.6122.

#### Synthesis of 7-(*tert*-butyl)-1-(4-(1,2,2-triphenylvinyl)phenyl)pyrene (Py-TPE)

Under a N_2_ atmosphere, a mixture of 1-bromo-7-(*tert*-butyl)pyrene (1 g, 3.03 mmol, 1 eq.), 4,4,5,5-tetramethyl-2-(4-(1,2,2-triphenylvinyl)phenyl)-1,3,2-dioxaborolane (1.38 g, 3.03 mmol, 1 eq.) and potassium carbonate (2.24 g, 18.19 mmol, 6 eq.) was dissolved in a mixed solution of tetrahydrofuran (40 mL) and H_2_O (20 mL) and stirred for 15 min at room temperature. Then, tetrakis(triphenylphosphine)palladium(0) (Pd(PPh_3_)_4_) (70 mg, 0.061 mmol) was added into the mixture and vigorously stirred at 68 °C for 24 h. After cooling to room temperature, the mixture was quenched with water (40 mL) and extracted three times with dichloromethane (3 × 60 mL), the combined organic phase was washed with saturated brine (60 mL), and dried with anhydrous magnesium sulfate (MgSO_4_), and the organic phase was evaporated. The residue was further purified by column chromatography with hexane/dichloromethane (*V*_Hexane_ : *V*_CH_2_Cl_2__ = 6 : 1) as the eluent to afford product Py-TPE as a white powder (1.5 g, yield: 84%). ^1^H NMR (400 MHz, CDCl_3_) *δ*_H_ 8.25 (d, *J* = 8.9 Hz, 2H), 8.17 (d, *J* = 7.8 Hz, 1H), 8.13 (d, *J* = 9.4 Hz, 1H), 8.07 (s, 2H), 8.03 (d, *J* = 9.3 Hz, 1H), 7.94 (d, *J* = 7.8 Hz, 1H), 7.41 (d, 2H), 7.25–7.13 (m, 18H), and 1.63 (s, 9H) ppm. ^13^C NMR (100 MHz, CDCl_3_) *δ* 149.2, 143.9, 143.8, 142.7, 141.4, 140.8, 139.3, 137.4, 131.5, 131.4, 130.9, 130.4, 129.9, 127.9, 127.8, 127.6, 127.4, 127.2, 126.6, 126.5, 125.2, 124.9, 124.5, 123.2, 122.4, 122.1, 35.3, and 32.0 ppm.

## Conclusion

In summary, we have developed intrinsically chiral TPE-decorated bipyrenyl-based enantiomers (*R-5/S-*5) that simultaneously overcome ACQ limitations and enable efficient chirality amplification. They display excellent chiroptical properties with a solid-state *Φ*_fl_ of up to 0.66 and an amplified |*g*_CD_| value of 10^−3^ and enhanced |*g*_lum_| values of ∼10^−4^*via* self-assembled aggregation behaviour. Moreover, the combination of SEM, CP-fs-TA spectroscopy and theoretical calculations reveals details on the dynamic chirality amplification in this pyrene system: (1) concentration-dependent CD and morphological evolution (from vesicles to helical fibers) confirm supramolecular chirality amplification. (2) SEM results directly visualize how aggregation-driven configurational reorganization in the excited state (*e.g.*, dihedral angle changes) underpins CPL enhancement. (3) Theoretical calculations demonstrate that the synergetic effect between excited-state configurational reorganization and optimized vector angle (*θ*_µ,m_) enables concurrent enhancement of both *Φ*_fl_ and |*g*_lum_| in the aggregated state. (4) Differential TA spectra reveal a hundred-fold order of prolongation of chirality amplification decay in thin films (>500 ps) compared to solution (<2 ps) within the TPE-decorated intrinsically chiral bipyrenyl-based AIEgen system. This extended chirality retention suppresses solution-phase CPL detection while amplifying the CPL signal with an enhanced |*g*_lum_| value in the aggregated state. Although the *g*_lum_ of these intrinsically chiral bipyrenyl-based AIEgens did not match that reported for self-assembled chiral systems (which achieve *g*_lum_ values on the order of 10^−2^ or higher), the high *Φ*_f_ of the bipyrenyl-based system is a notable merit. This work not only provides a new chiral source for constructing new CPL materials *via* a molecular design strategy, but also elucidates the dynamic chirality amplification mechanism in this new system. The bipyrenyl framework serves as a novel chiral skeleton for advanced CPL materials, demonstrating promising potential in 3D displays, chiral sensing, and quantum information technologies.

## Author contributions

Z. Xie: methodology, data curation, investigation; J. Deng: data curation, investigation; D. Liu: data curation; J. Lin: data curation; T. Jiang: data curation; X. Wang: investigation; W. Liu: data curation; L. Ma: investigation, writing – review & editing; F. Song: data curation, investigation, writing – review & editing; Z. Xiong: data curation; J. Chen: methodology; J. Zhang: data curation, investigation; C. Redshaw: writing – review & editing; Z. Zhao: investigation, writing – review & editing; X. Feng: supervision, writing – review & editing, funding acquisition; B. Tang: source, writing – review & editing.

## Conflicts of interest

There is no conflict of interest to report.

## Supplementary Material

SC-017-D5SC08358C-s001

## Data Availability

Supplementary information (SI): experimental procedures, characterization data of the compounds (NMR, HRMS, UV-vis and emission spectra, CD/CPL spectra, high performance liquid chromatography, and scanning electron microscope analysis), DFT calculations and fs-TA data. See DOI: https://doi.org/10.1039/d5sc08358c.
