# Supplementary material for: Unlocking intrinsically chiral bipyrenyl-based aggregation-induced emission luminogens: circularly polarized luminescence and dynamic chirality amplification
Source: Chem Sci. 2026 Jan 7;17(8):4145–56. doi: 10.1039/d5sc08358c (PMC12776029; doi:10.1039/d5sc08358c)
Supplement: SC-017-D5SC08358C-s001 [file SC-017-D5SC08358C-s001.pdf]

Supplementary Information (SI) for Chemical Science.
This journal is © The Royal Society of Chemistry 2025

# Unlocking Intrinsically Chiral Bipyrenyl-based Aggregation-induced Emission Luminogens: Circularly Polarized Luminescence and Dynamic Chirality Amplification

Zhixin Xie,[1‡] Junpeng Deng,[2‡] Dan Liu,[3‡] Jieyu Lin,[1‡] Tao Jiang,[1] Xiaohui Wang,[1] Wei Liu,[1] Lin Ma,[2*] Fengyan Song,[4*] Zuping Xiong,[5] Junru Chen,[1] Jianyu Zhang,[5*] Carl Redshaw,[6] Zujin Zhao,[7] Xing Feng,[1*] and Ben Zhong Tang[3*]

[1] Guangdong Provincial Key Laboratory of Functional Soft Condensed Matter, School of Materials and Energy, Guangdong University of Technology, Guangzhou 510006, P. R. China. E-mail: hyxhn@sina.com

[2] School of Physics and Optoelectronic Engineering, Guangdong Provincial Key Laboratory of Sensing Physics and System Integration Applications, Institute for Frontier Physics and Advanced Instruments, Guangdong University of Technology, Guangzhou 510006, P. R. China. Email: malin@gdut.edu.cn

[3] Guangdong Basic Research Center of Excellence for Aggregate Science, School of Science and Engineering, The Chinese University of Hong Kong, Shenzhen, Guangdong 518172, China. E-mail: tangbenz@cuhk.edu.cn

[4] Center of Excellence for Environmental Safety and Biological Effects, Beijing Key Laboratory for Green Catalysis and Separation, Department of Chemistry, College of Chemistry and Life Science, Beijing University of Technology, Beijing, China. E-mail: fengyansong@bjut.edu.cn.

[5] State Key Laboratory (SKL) of Biobased Transportation Fuel Technology, Department of Polymer Science and Engineering, Zhejiang University, Hangzhou 310058, China. E-mail: zhangjianyu@zju.edu.cn

[6] Department of Chemistry, Graduate School of Science, Tokyo Metropolitan University, 1-1 Minami Osawa, Hachioji, Tokyo 1920397, Japan.

[7] State Key Laboratory of Luminescent Materials and Devices, Guangdong Provincial Key Laboratory of Luminescence from Molecular Aggregates, South China University of Technology, Guangzhou 510640, P. R. China

*Corresponding Author(s): Xing Feng: hyxhn@sina.com, Lin Ma: malin@gdut.edu.cn, Ben Zhong Tang: tangbenz@cuhk.edu.cn, Feng Yan Song: fengyansong@bjut.edu.cn, Jianyu Zhang: zhangjianyu@zju.edu.cn. Z. Xie, J. Deng, D. Liu and J. Lin contributed equally to this work.

Table of Contents

1. Experimental section......S3
2. NMR spectra......S4
3. High-Resolution Mass Spectrometry (HRMS)......S12
4. High Performance Liquid Chromatography ......S15
5. Theoretical Calculations ......S16
6. Photophysical Properties......S21
7. Circular Dichroism Spectroscopy......S24
8. Circularly Polarized Luminescence Spectroscopy......S26
9. Scanning Electron Microscope Analysis ......S29
10. Femtosecond Transient Absorption Spectroscopy Analysis......S31
References......S56

# 1. Experimental section

## 1.1 Materials

Unless otherwise stated, all reagents used were purchased from commercial sources and were used without further purification. Specpure grade solvents were used for spectroscopic measurements. The starting compound 7-*tert*-butyl-2-hydroxypyrene (**Py-OH**) was synthesized following the previously reported procedure.[1 2]

## 1.2 Synthetic Procedures

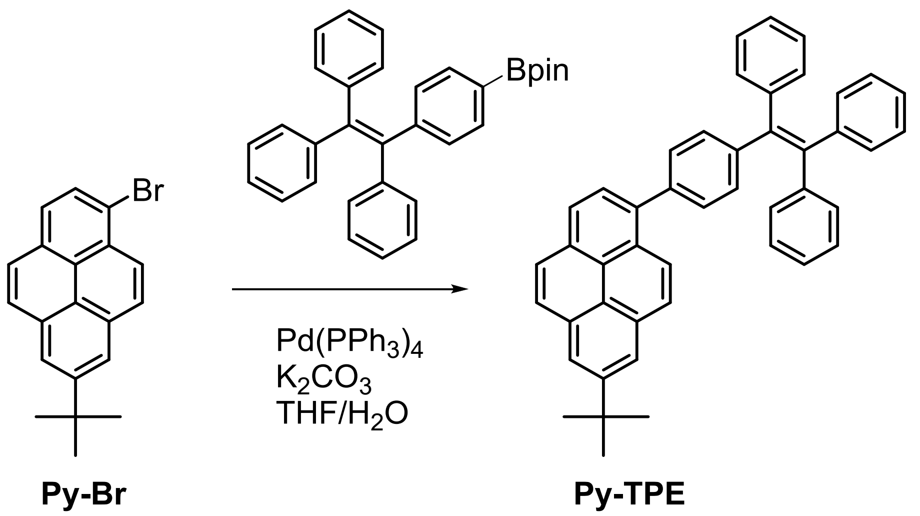


Scheme S1. Synthetic route for Py-TPE

## 2. NMR spectra

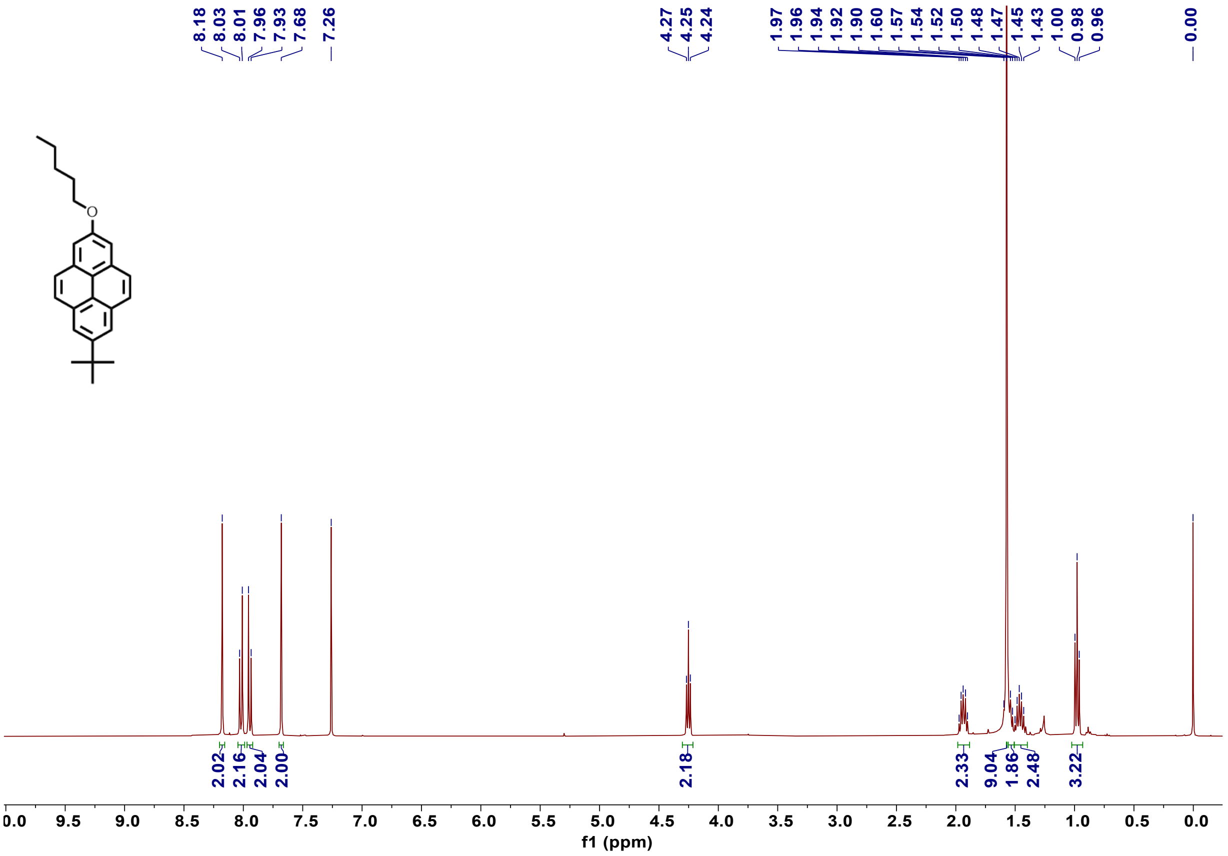


**Figure S1**. $^{1}$H-NMR spectrum (400 MHz, 293 K, *$CDCl_3$) for **1**.

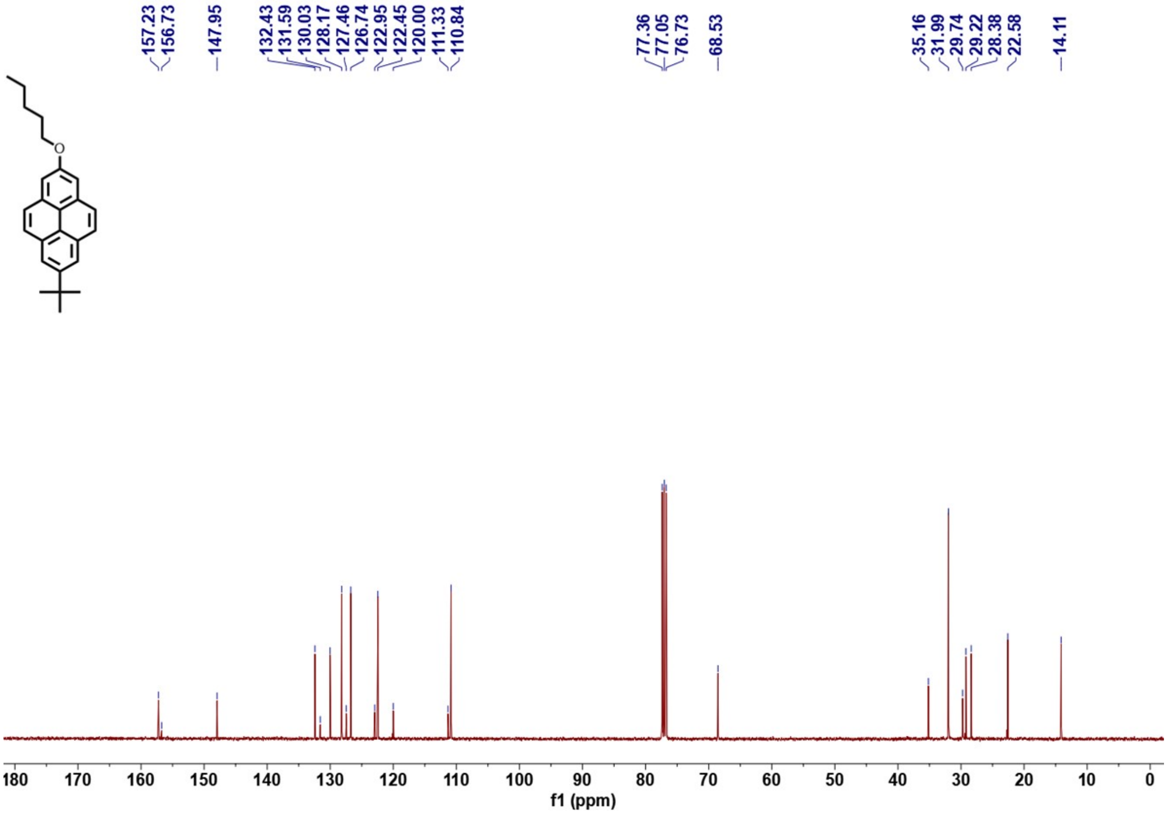


**Figure S2**. $^{13}$C-NMR spectrum (100 MHz, 293 K, *$CDCl_3$) for **1**.

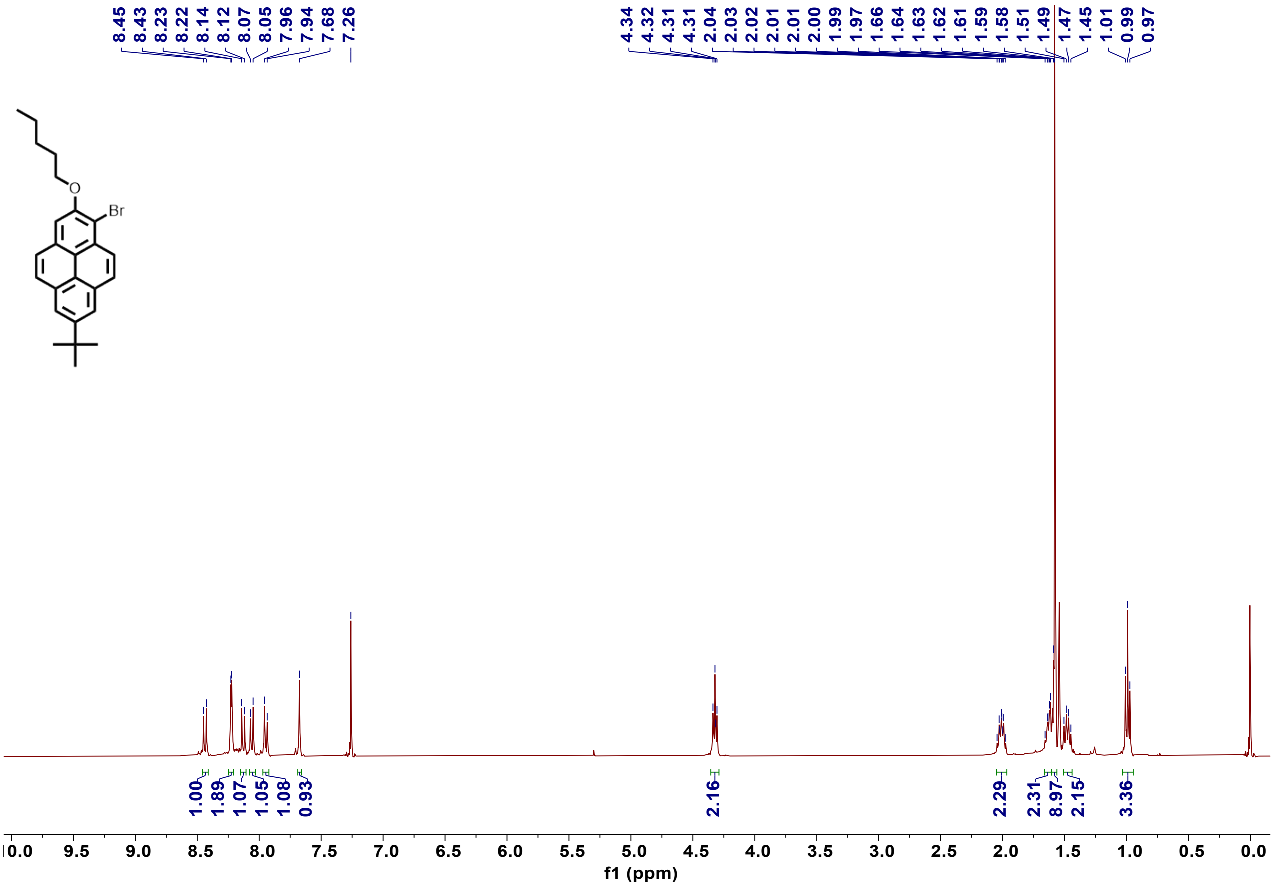

**Figure S3**. $^{1}$H-NMR spectrum (400 MHz, 293 K, *$CDCl_3$) for **2**.

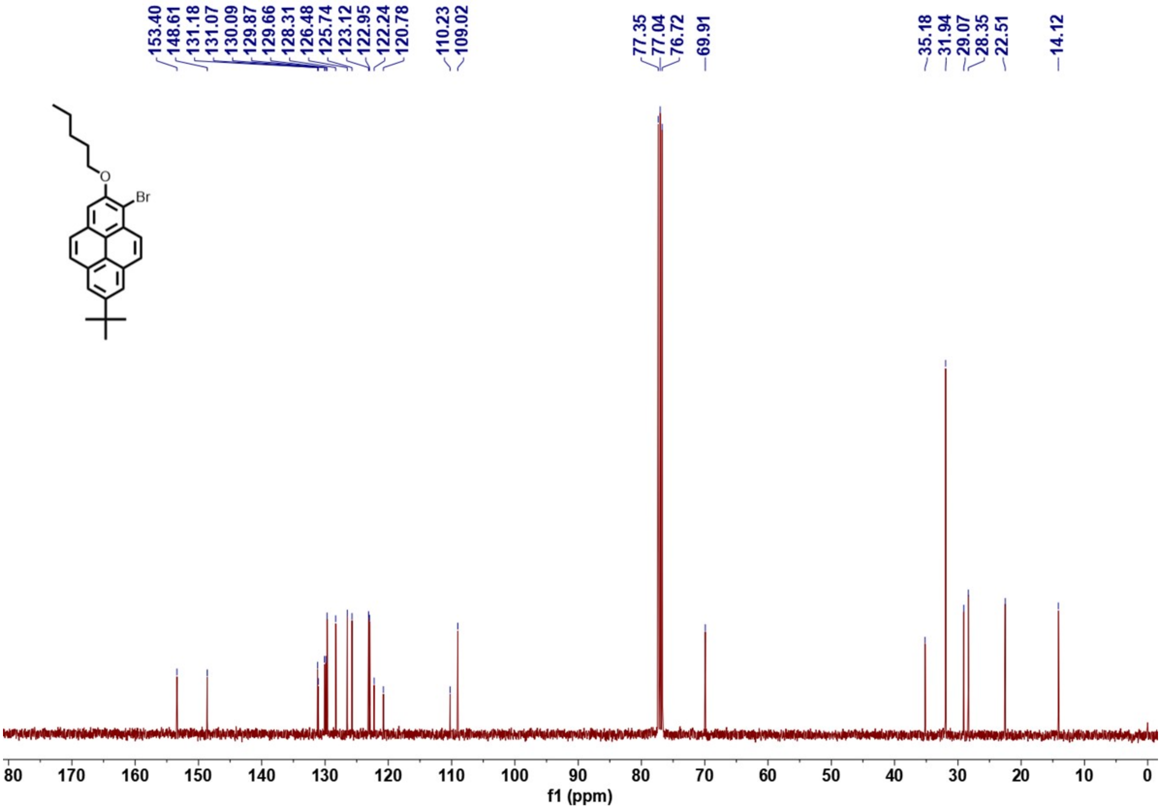

**Figure S4**. $^{13}$C-NMR spectrum (100 MHz, 293 K, *$CDCl_3$) for **2**.

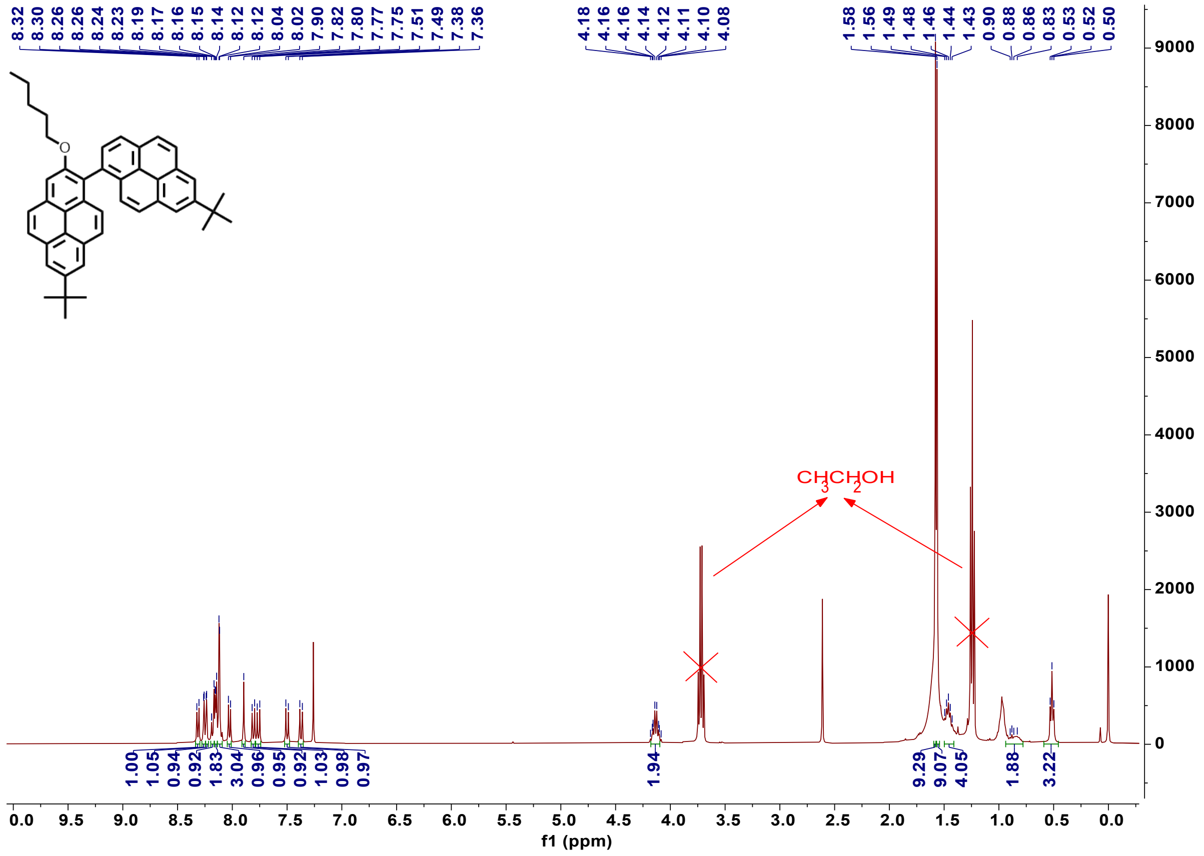


**Figure S5**. $^{1}$H-NMR spectrum (400 MHz, 293 K, *$CDCl_3$) for **3**.

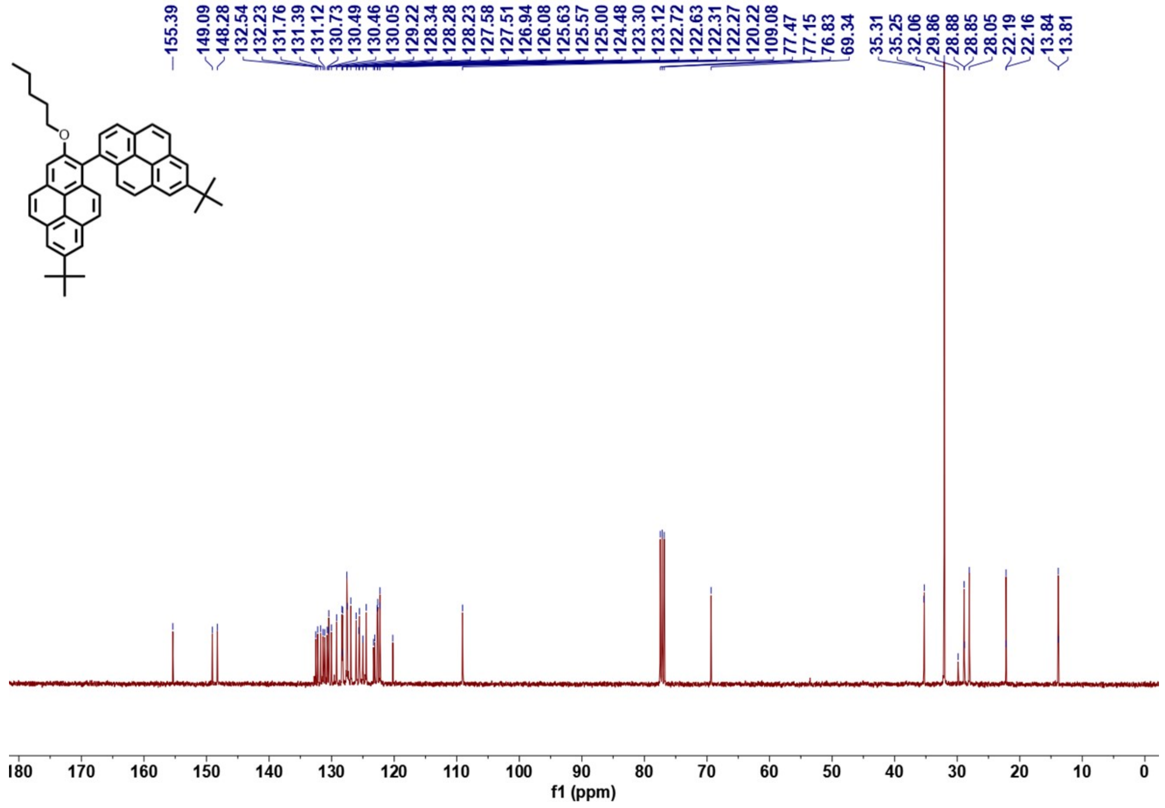


**Figure S6**. $^{13}$C-NMR spectrum (100 MHz, 293 K, *$CDCl_3$) for **3**.

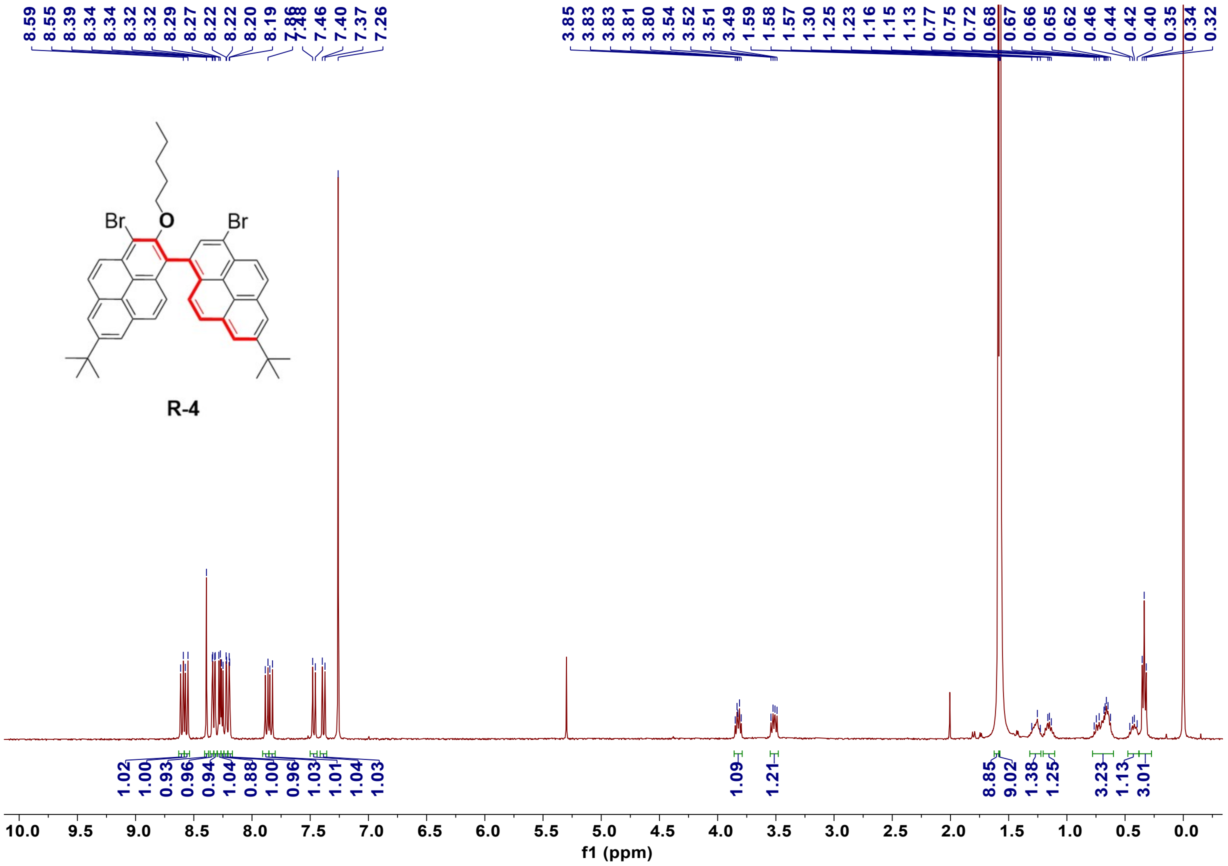


**Figure S7**. $^{1}H$-NMR spectrum (400 MHz, 293 K, *$CDCl_3$) for ***R*-4**.

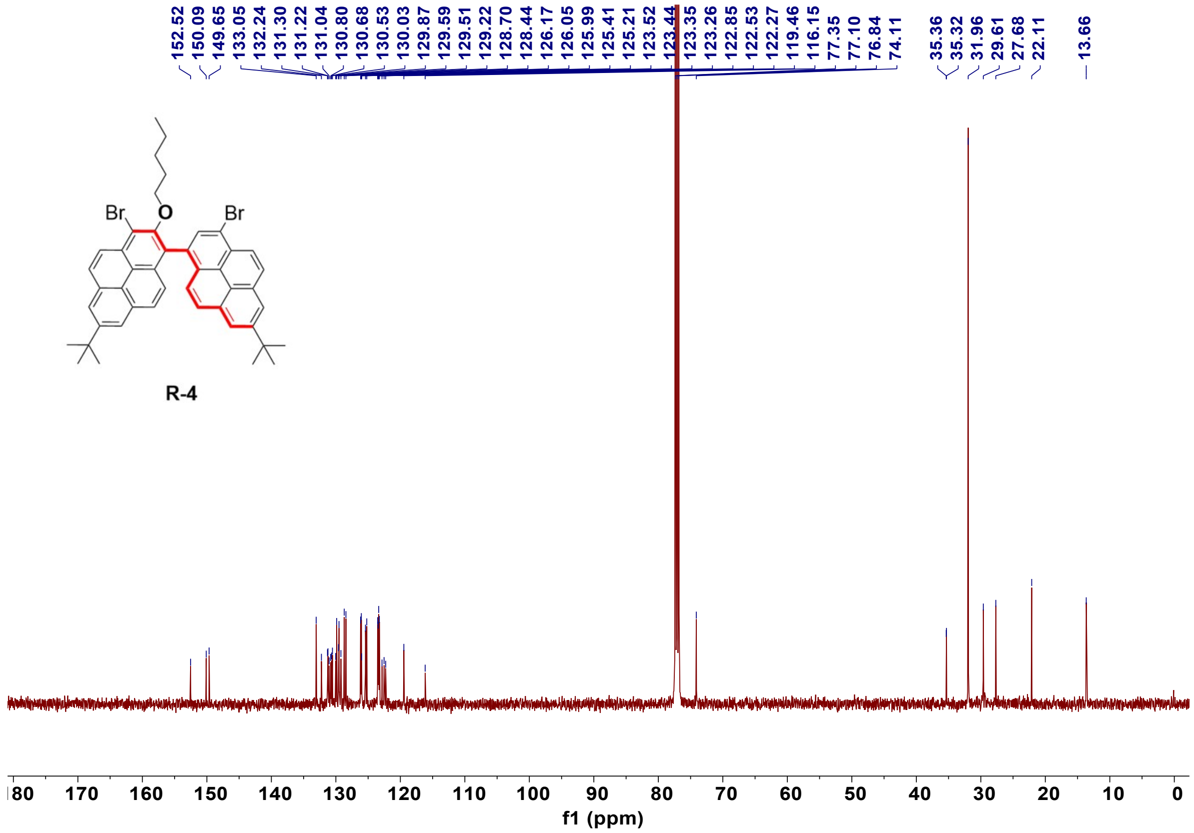


**Figure S8**. $^{13}C$-NMR spectrum (100 MHz, 293 K, *$CDCl_3$) for ***R*-4**.

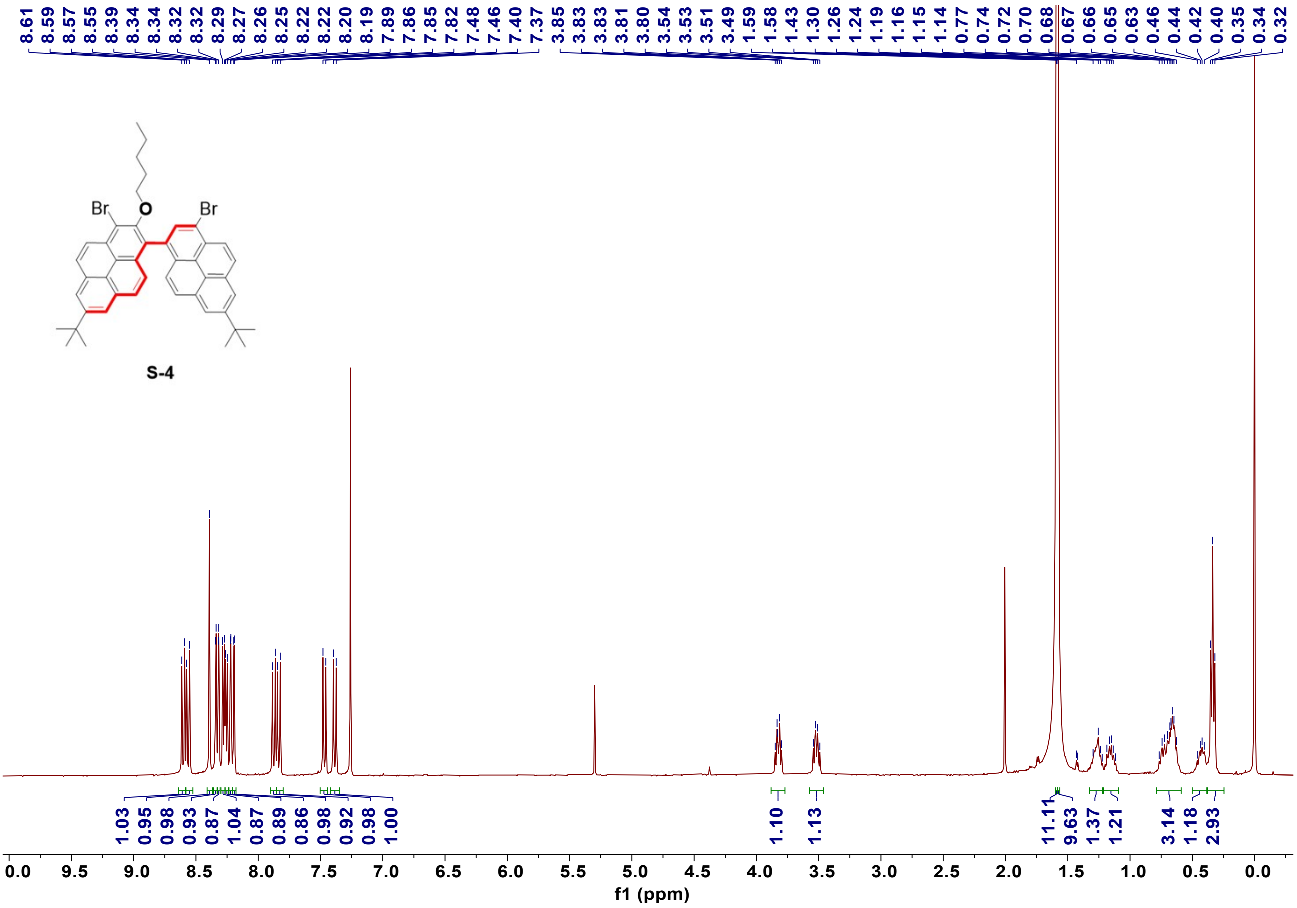


**Figure S9**. $^{13}$C-NMR spectrum (100 MHz, 293 K, *$CDCl_3$) for ***S*-4**.

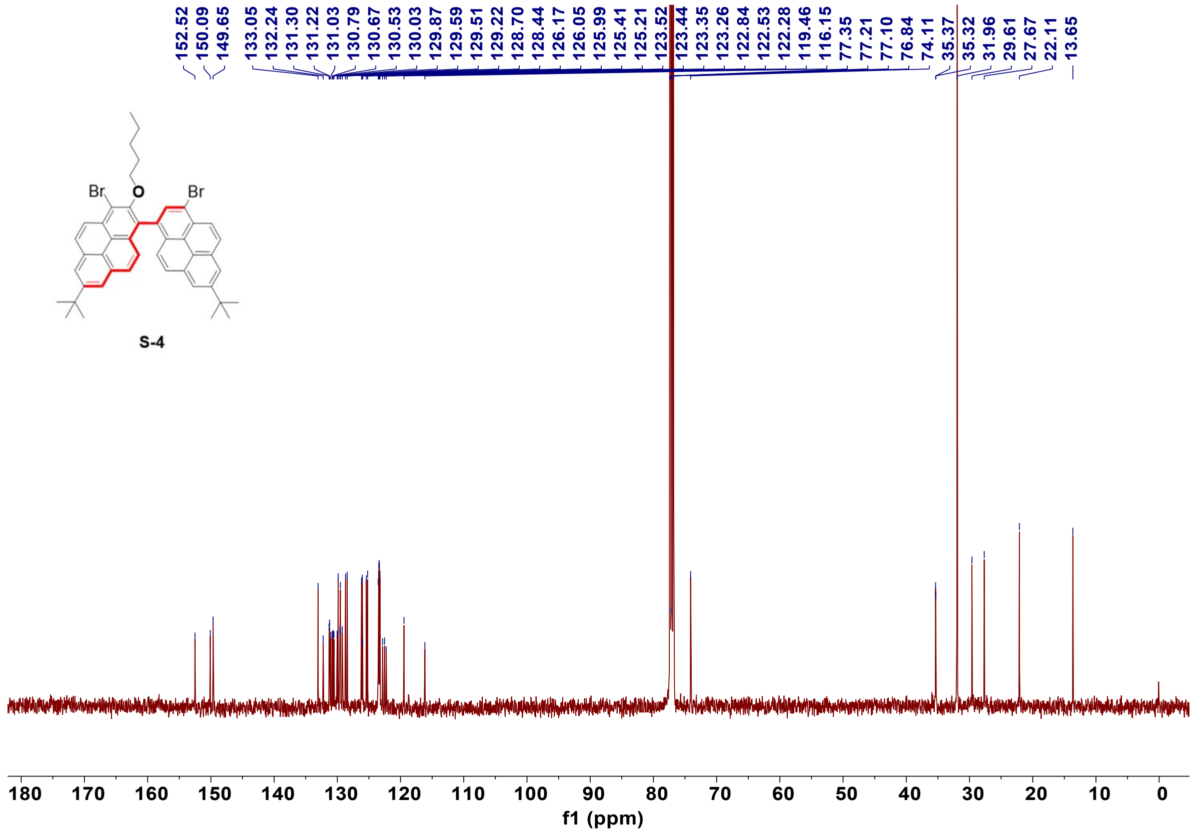


**Figure S10**. $^{13}$C-NMR spectrum (100 MHz, 293 K, *$CDCl_3$) for ***S*-4**.

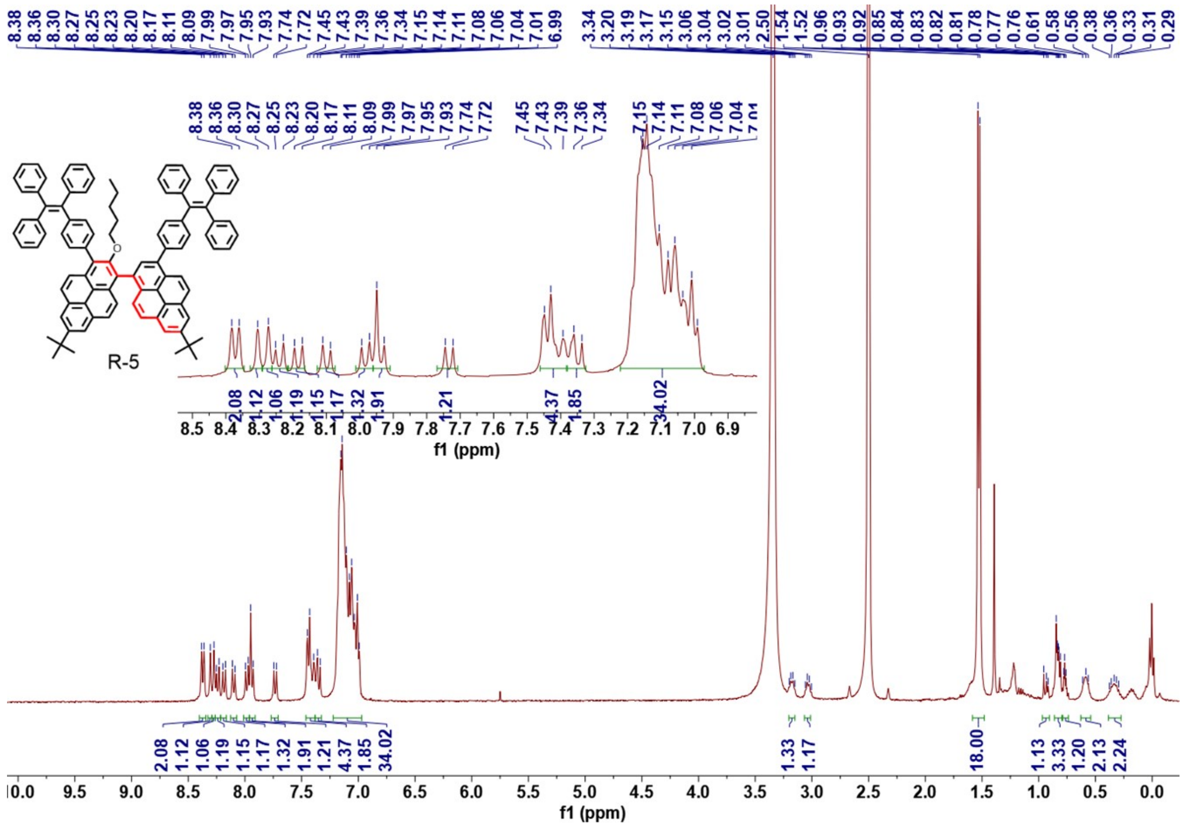


**Figure S11**. $^{1}$H-NMR spectrum (400 MHz, 293 K, DMSO-$d_6$) for ***R*-5**.

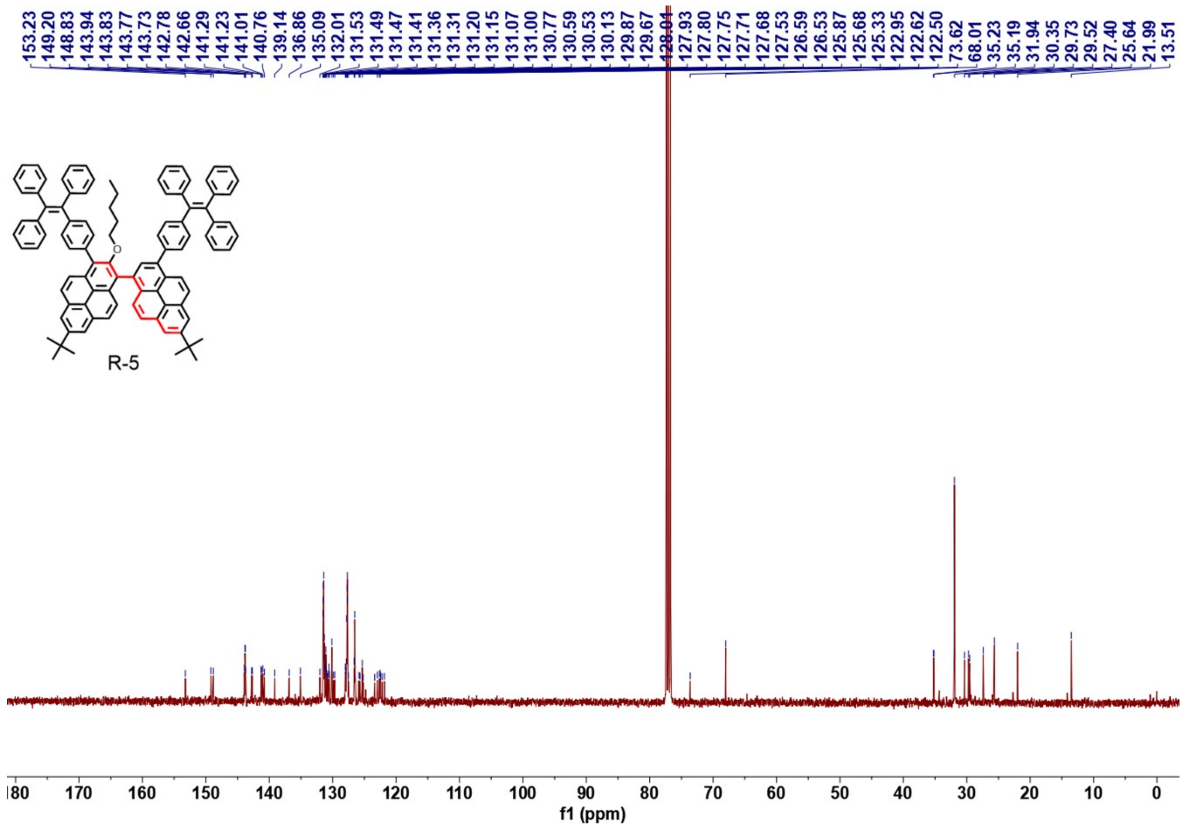


**Figure S12**. $^{13}$C-NMR spectrum (100 MHz, 293 K, *$CDCl_3$) for ***R*-5**.

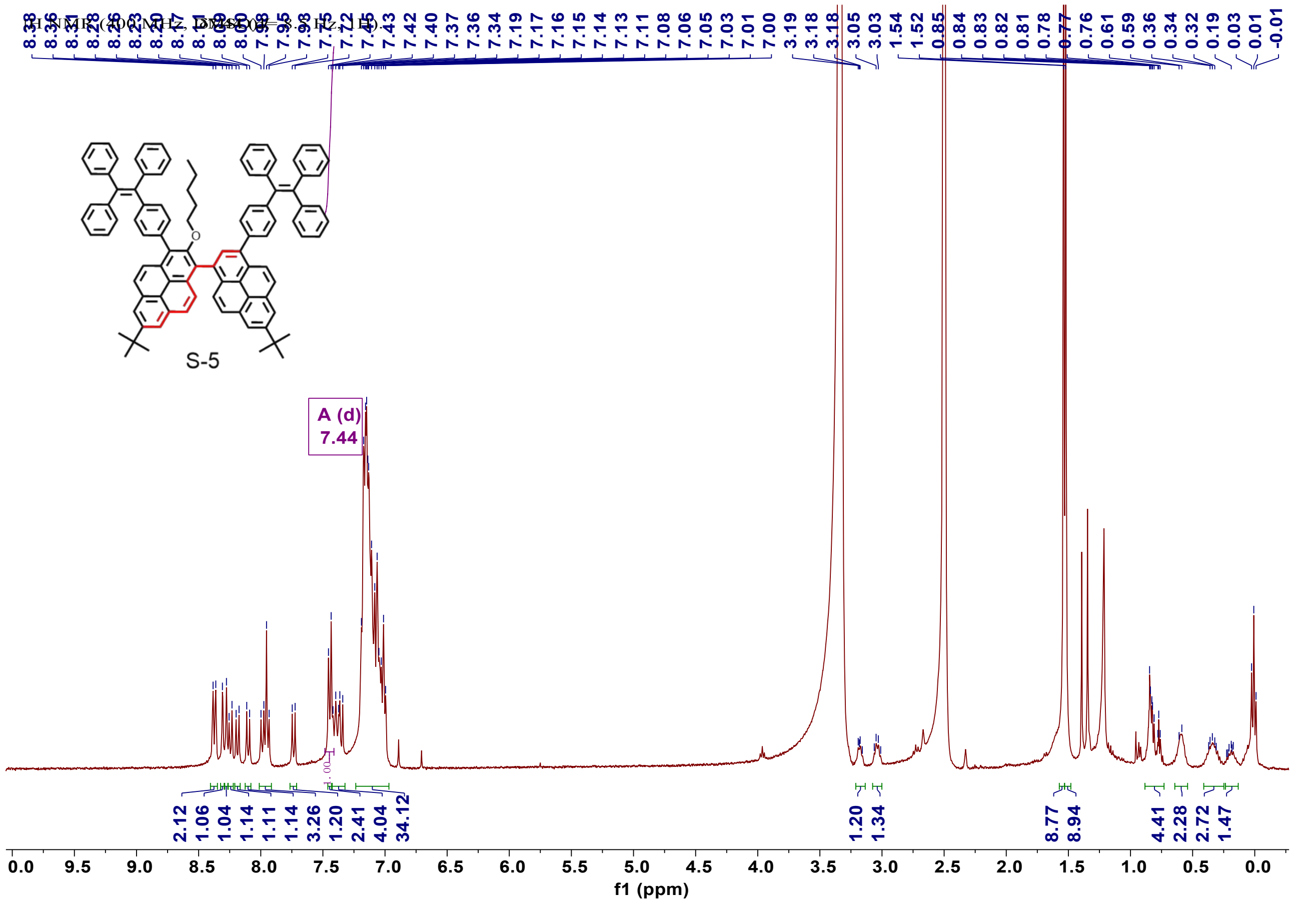

**Figure S13**. $^{1}$H-NMR spectrum (400 MHz, 293 K, DMSO-$d_6$) for ***S*-5**.

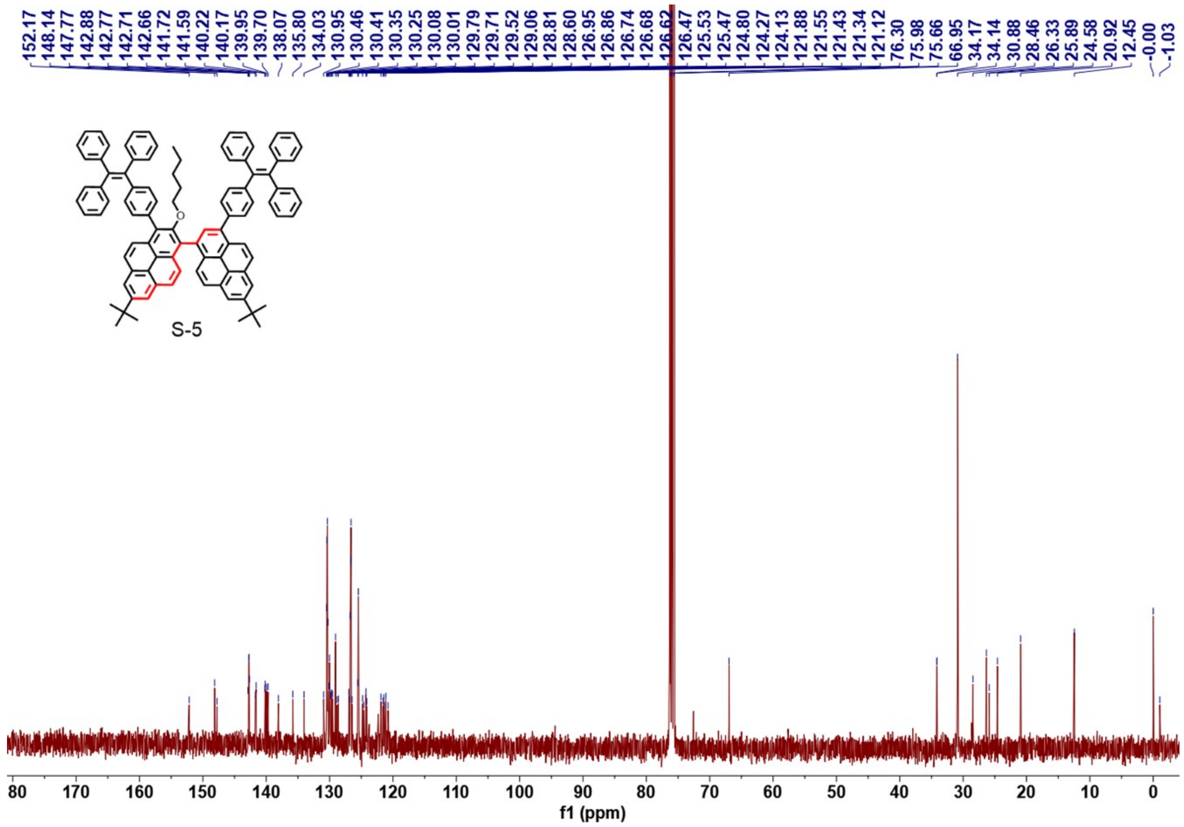

**Figure S14**. $^{13}$C-NMR spectrum (100 MHz, 293 K, *$CDCl_3$) for ***S*-5**.

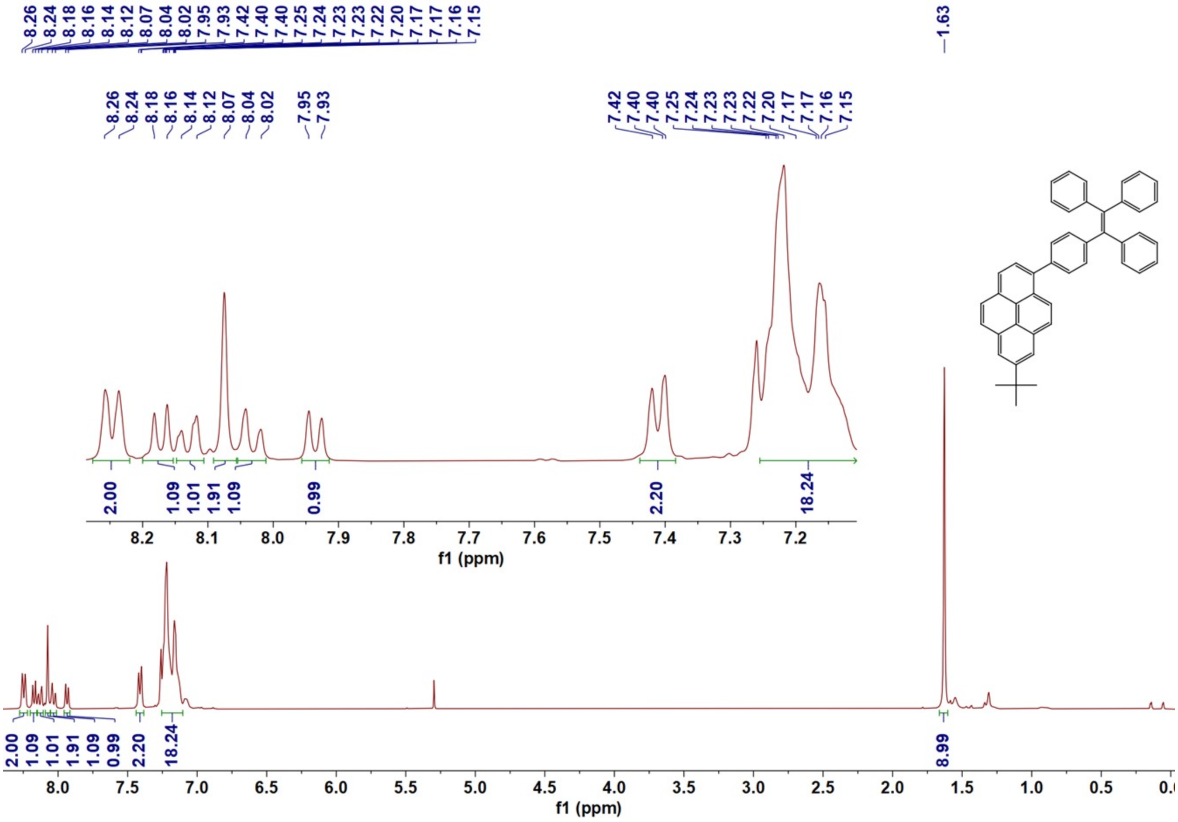


**Figure S15** $^{1}$H-NMR spectrum (400 MHz, 293 K, * $CDCl_3$) for **Py-TPE**

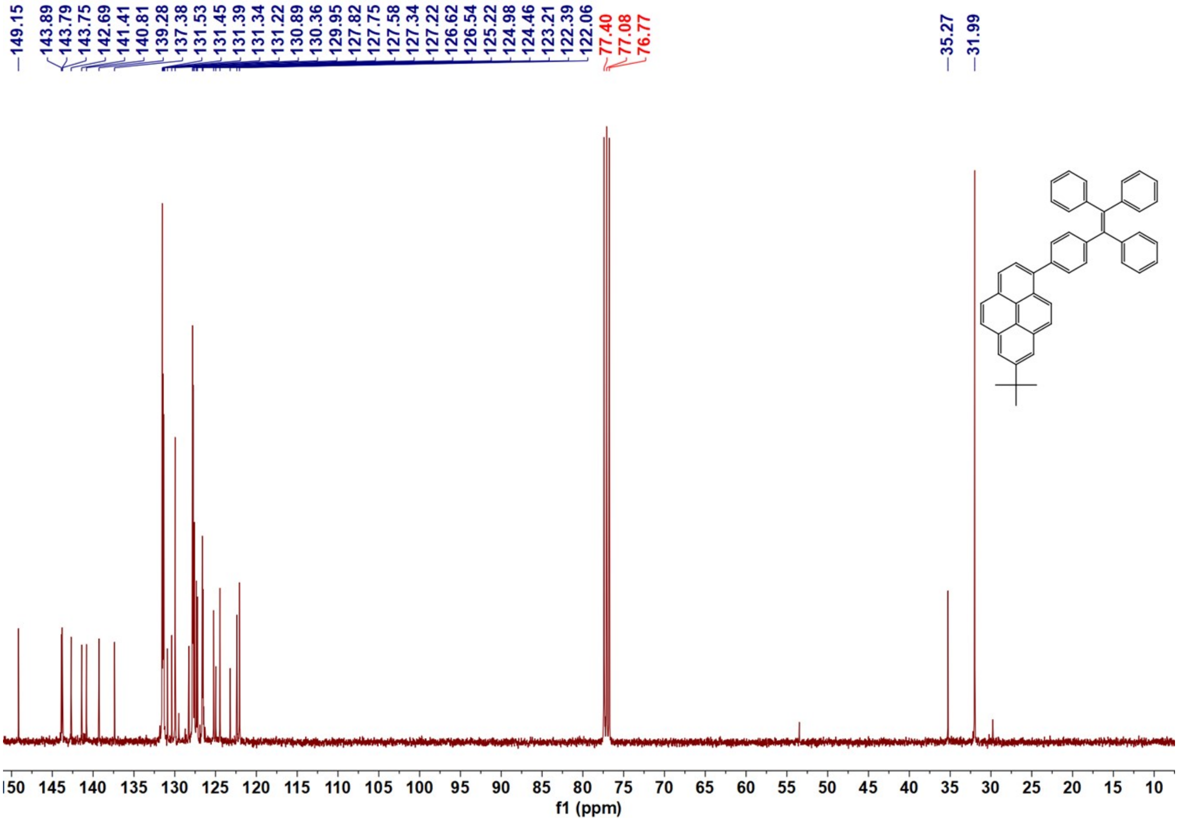


**Figure S16** $^{13}$C-NMR spectrum (100 MHz, 293 K, * $CDCl_3$) for **Py-TPE**

## 3. High-Resolution Mass Spectrometry (HRMS)

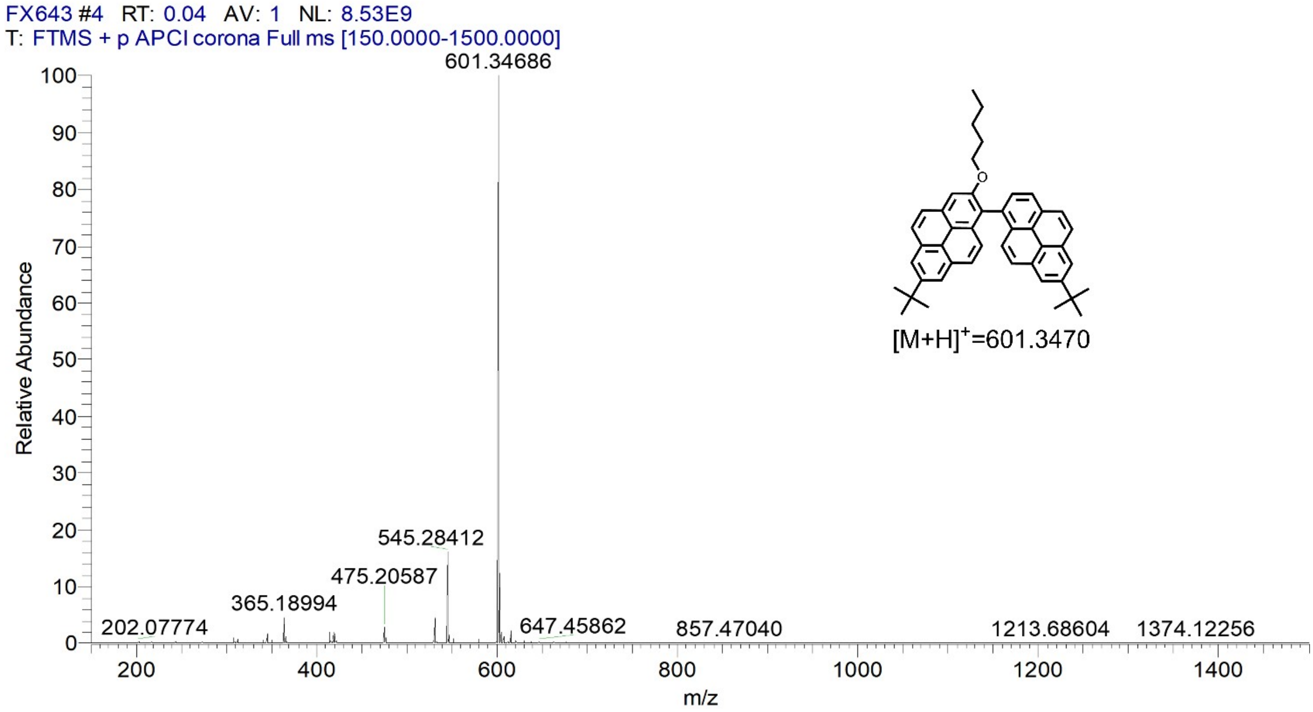


**Figure S17**. HRMS spectra of *racemic* **3**.

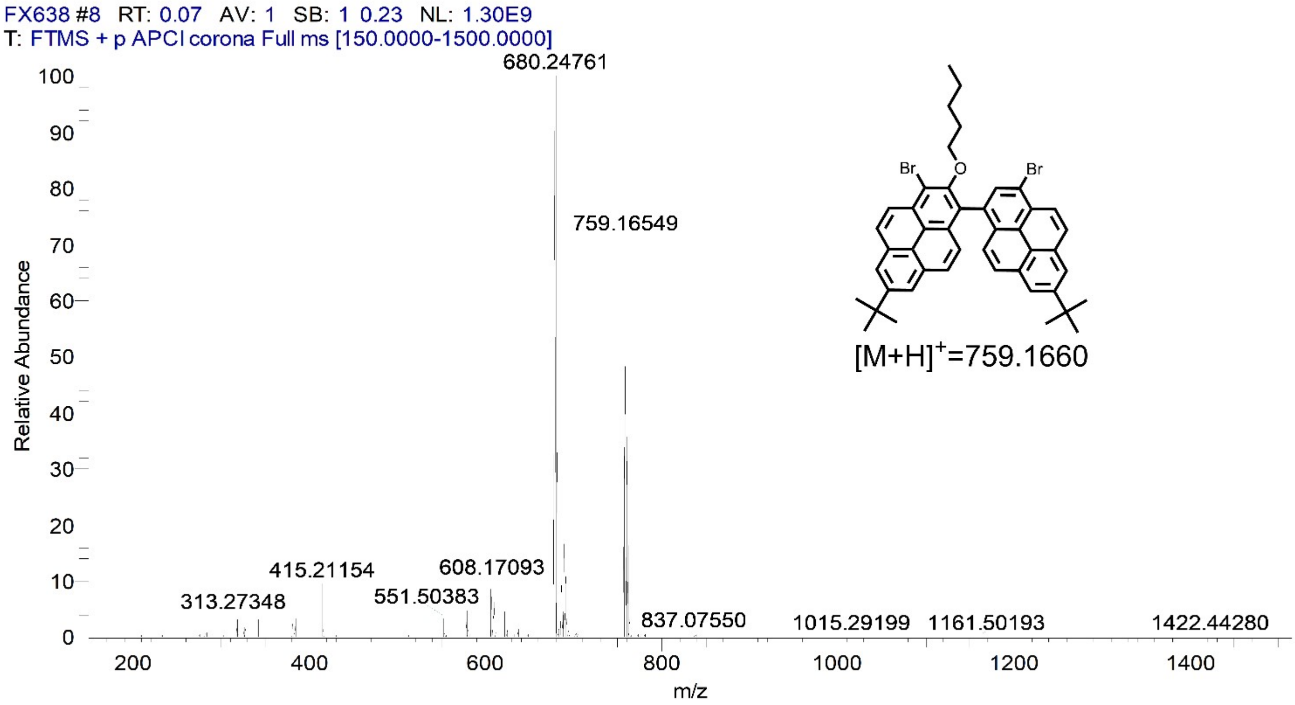


**Figure S18**. HRMS spectra of *racemic* **4**.

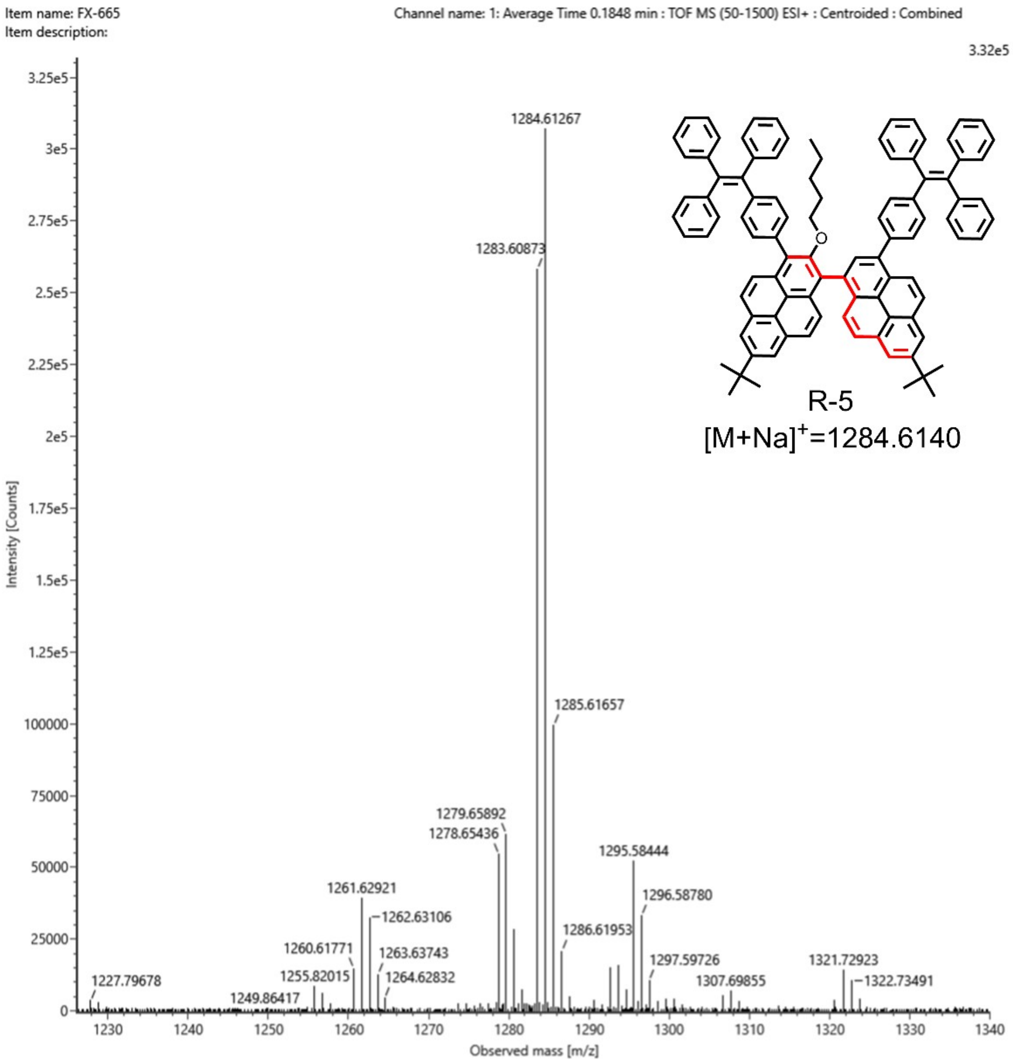


**Figure S19**. HRMS spectra of ***R*-5**.

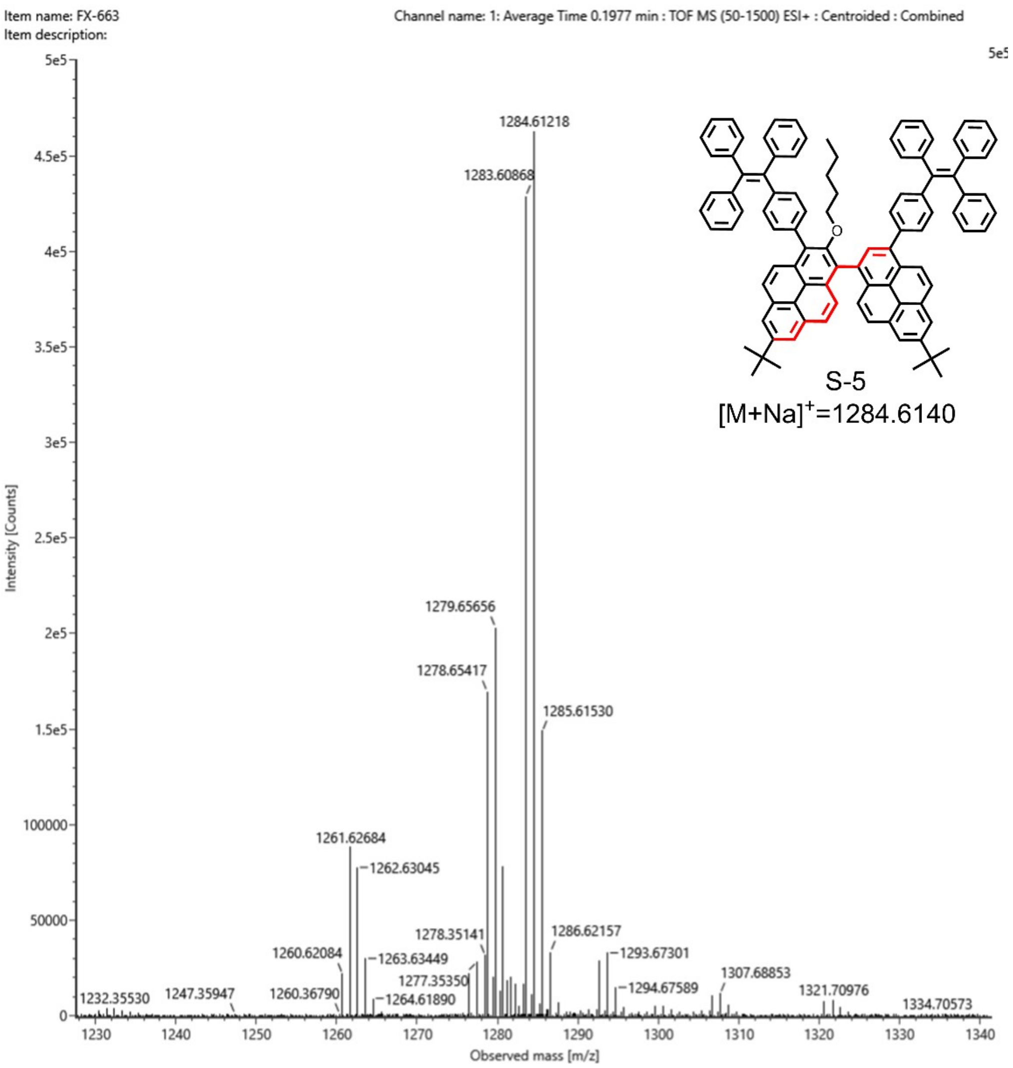


**Figure S20**. HRMS spectra of ***S*-5**.

# 4. High Performance Liquid Chromatography

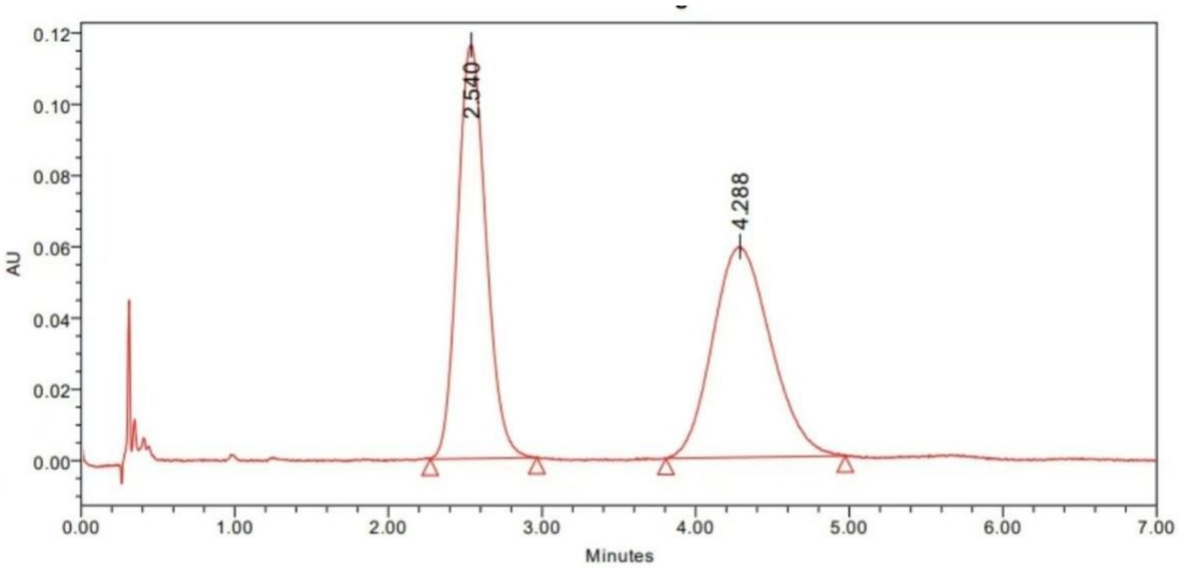


Peak Results

| | RT | USP Tailing | EP Plate Count | Resolution | Height | % Height | Area | % Area |
|---|---|---|---|---|---|---|---|---|
| 1 | 2.540 | | | | 116076 | 66.22 | 1501961 | 49.08 |
| 2 | 4.288 | | | | 59200 | 33.78 | 1558116 | 50.92 |

**Figure S21**. Chiral HPLC chromatograms of ***R*-/*S*-5** collected at $\lambda_{ex}$ = 210 nm excitation (Column: Daicel ChiralPak IH, 3mm I.D.×100mm, 3um, Mobile phase: A for $CO_2$ and B for MeOH, Gradient: B 35% in 7 min, Flow rate: 2.0 mL/min, Column temperature: 35°C).

## 5. Theoretical Calculations

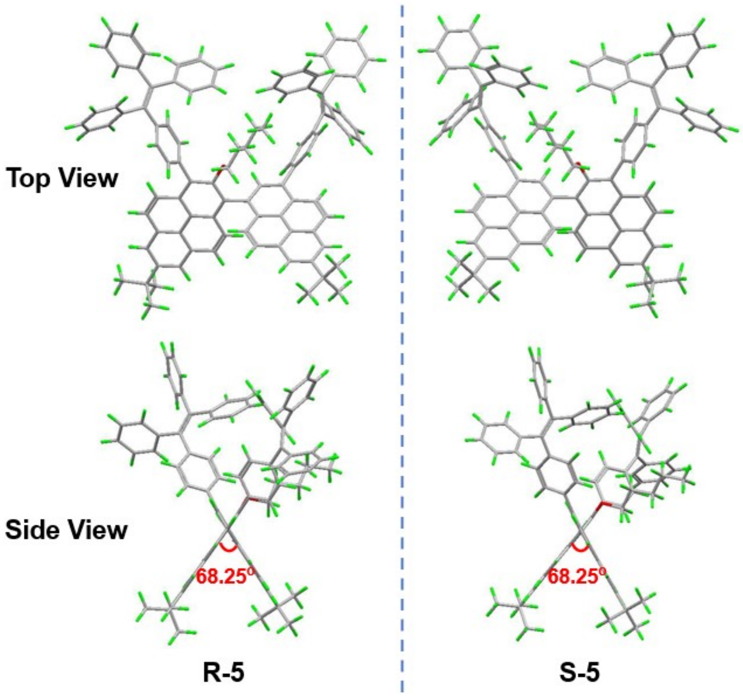


**Figure S22**. The optimized geometric molecular structure of ***R*-5** and ***S*-5** at ground state (CAM-B3LYP/6-311G(d,p)).

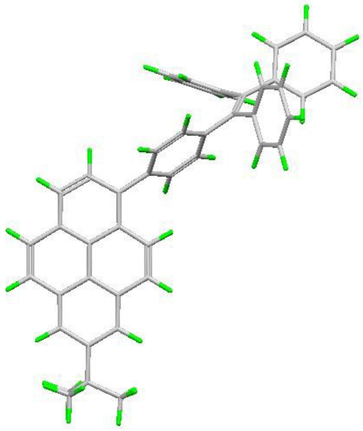

**Figure S23**. The optimized geometric molecular structure of **Py-TPE** at ground state (CAM-B3LYP/6-311G(d,p)).

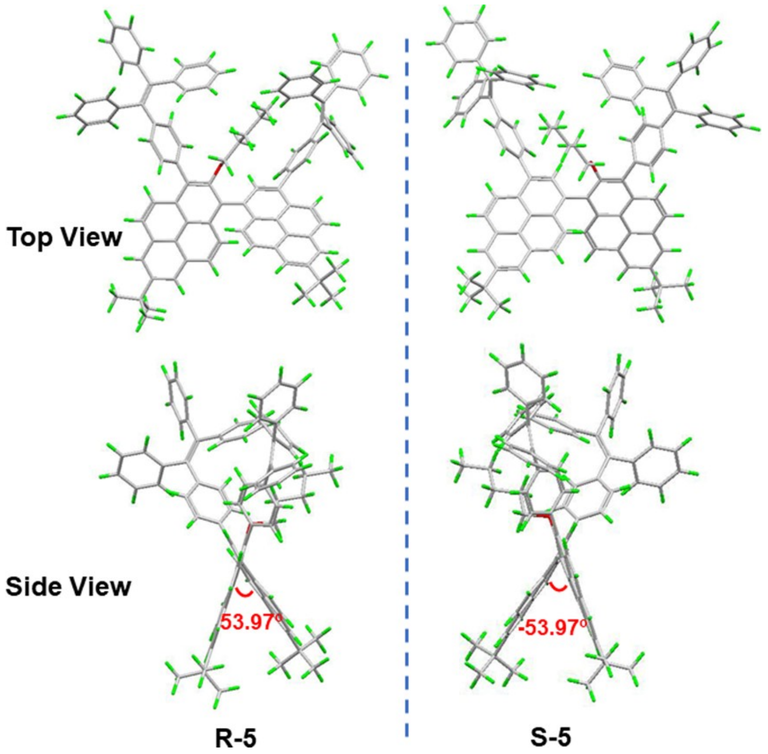


**Figure S24**. The optimized geometric molecular structure of ***R*-5** and ***S*-5** at excited state (CAM-B3LYP/6-311G(d,p)).

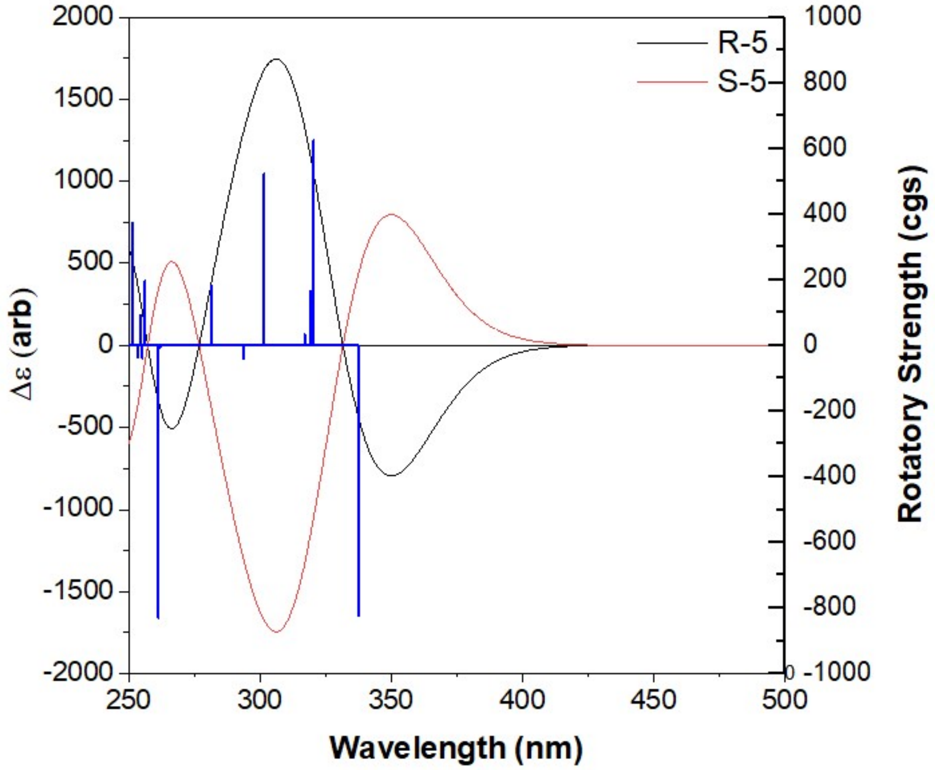


**Figure S25**. Simulated CD spectra of ***R*/*S*-5** based on the optimized geometric structure at ground state (CAM-B3LYP/6-311G(d,p)).

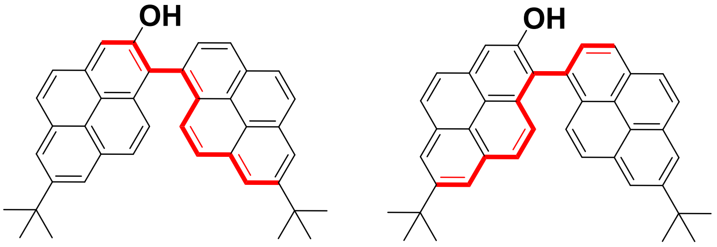


***R*-dipyrenol** ***S*-dipyrenol**

**Scheme S1**. Molecular structure of ***R/S*-dipyrenol**.

**Table S1**. Calculated of the parameters of the transition electric dipole moment ($\mu$) and transition magnetic dipole moment ($m$), the $\theta_{\mu,m}$ and $g_{cal\ abs}$ at the ground state[a]

| Compounds | $\mu$ | $m$ | $\theta_{\mu,m}$ | $g_{cal}$ |
|---|---|---|---|---|
| ***R/S*--dipyrenol** | $5.92\times10^{-18}$ | $2.53\times10^{-20}$ | 100.5° | $3.12\times10^{-3}$ |
| ***R/S*-3** | $7.38\times10^{-18}$ | $4.38\times10^{-20}$ | 75.6° | $5.90\times10^{-3}$ |
| ***R/S*-5** | $7.01\times10^{-18}$ | $3.31\times10^{-20}$ | 44.1° | $1.35\times10^{-2}$ |

[a] Calculated by CAM-B3LYP/6-311G(d,p)

**Table S2**. Calculated of the parameters of the transition electric dipole moment ($\mu$) and transition magnetic dipole moment ($m$), the $\theta_{\mu,m}$ and $g_{cal\ lum}$ at the excited state[a]

| Compounds | $\mu$ | $m$ | $\theta_{\mu,m}$ | $g_{cal}$ |
|---|---|---|---|---|
| ***R/S*--chiral dipyrenol** | $7.12\times10^{-18}$ | $4.82\times10^{-20}$ | 81.9° | $3.82\times10^{-3}$ |
| ***R/S*-3** | $7.36\times10^{-18}$ | $6.81\times10^{-20}$ | 76.4° | $8.70\times10^{-4}$ |
| ***R/S*--5** | $7.97\times10^{-18}$ | $1.32\times10^{-20}$ | 55.3 | $3.77\times10^{-3}$ |

[a] Calculated by CAM-B3LYP/6-311G(d,p)

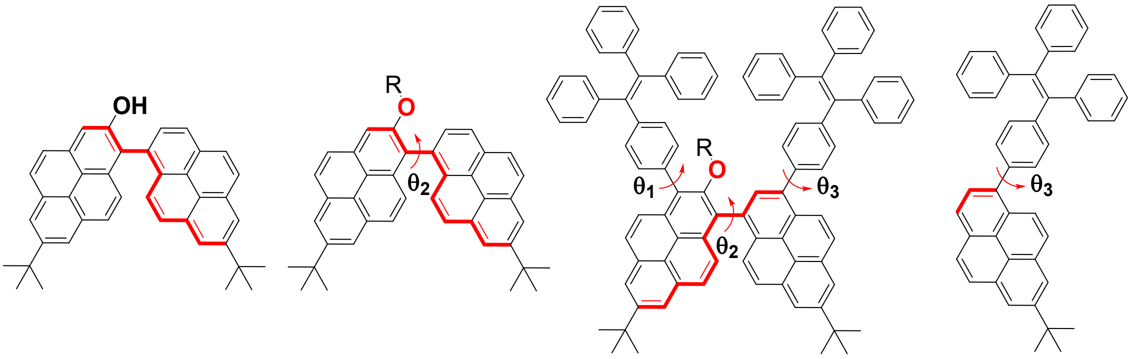


***R-/S*-dipyrenol** **R-/S-3** **R-/S-5** **Py-TPE**

**Table S3**. Calculated twist angle[a]

| Compounds | $\theta_1$ | $\theta_2$ | $\theta_3$ |
|---|---|---|---|
| ***R/S*--chiral dipyrenol** | - | 97.7[b] / 46.9[c] | - |
| ***R/S*-3** | - | 69.8[b] / 46.3[c] | - |
| ***R/S*-5** | 80.7[b] / 61.6[c] | 64.9[b] / 48.3[c] | 53.5[b] / 53.5[c] |
| **Py-TPE** | - | - | 57.1[b] / |

[a] Calculated by CAM-B3LYP/6-311G(d,p)
[b] at ground state
[c] at excited state

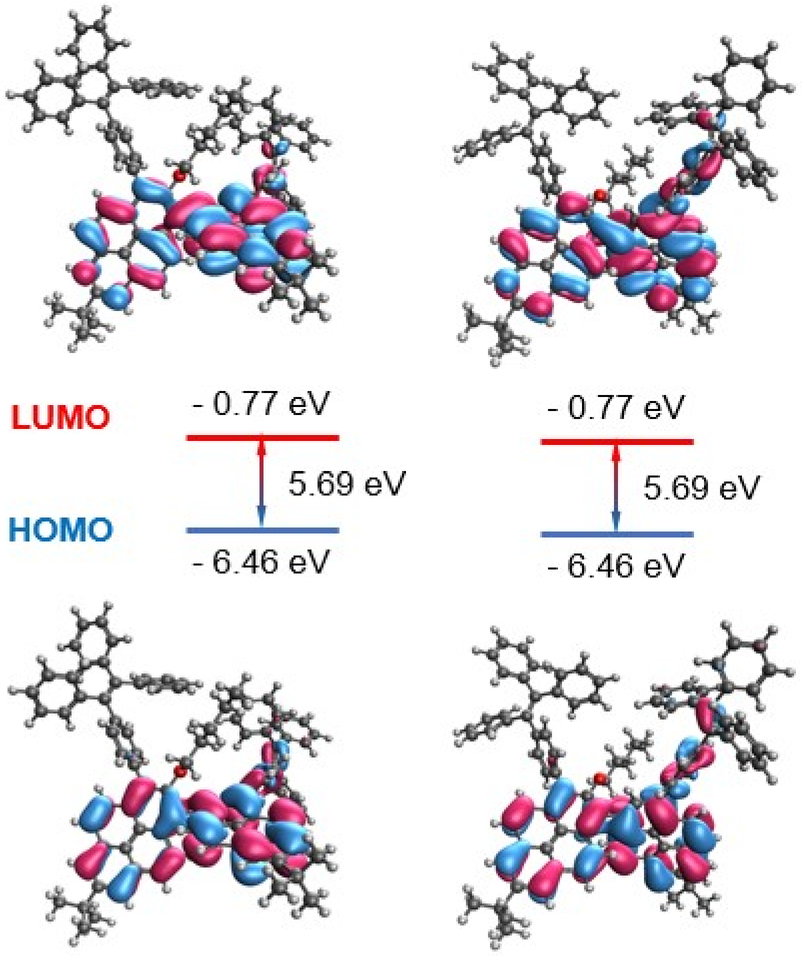


**Figure S26.** Molecular orbital plots of compounds ***R*/*S*-5** calculated at the ground state (CAM-B3LYP/6-311G(d,p) level).

**Table S4.** Excitation energies calculated at CAM-B3LYP/6-311G(d,p) optimized geometries.

| Compound | Excitation | E/eV | λ/nm | Osc. Strength | *i*→*a* | $k_{ia}$ |
|---|---|---|---|---|---|---|
| ***R*-5** | $S_0 \rightarrow S_1$ | 2.99 | 415 | 0.7204 | H-1→L+1 | 2.3 |
| | | | | | H→L | 93.3 |
| | $S_0 \rightarrow S_2$ | 3.54 | 350 | 0.8698 | H-5→L | 3.2 |
| | | | | | H-4→L+1 | 3.7 |
| | | | | | H-3→L | 3.6 |
| | | | | | H-1→L | 68.4 |
| | | | | | H→L+1 | 6.6 |
| | | | | | H→L+5 | 4.5 |
| | $S_0 \rightarrow S_3$ | 3.58 | 346 | 0.3502 | H-5→L | 5.7 |
| | | | | | H-4→L | 2.6 |
| | | | | | H-4→L+1 | 3.1 |
| | | | | | H-1→L+4 | 3.8 |
| | | | | | H→L+1 | 68.5 |
| | | | | | H→L+5 | 5.0 |
| | $S_0 \rightarrow S_4$ | 3.70 | 335 | 0.0311 | H-5→L | 6.87 |
| | | | | | H-5→L+1 | 8.85 |
| | | | | | H-4→L | 32.1 |
| | | | | | H-2→L | 7.38 |
| | | | | | H-1→L+5 | 7.02 |
| | | | | | H→L+4 | 22.7 |
| ***S*-5** | $S_0 \rightarrow S_1$ | 3.03 | 409 | 0.7318 | H-1→L+1 | 2.6 |
| | | | | | H→L | 93.3 |
| | $S_0 \rightarrow S_2$ | 3.56 | 348 | 0.8283 | H-5→L | 7.5 |
| | | | | | H-1→L | 62.5 |
| | | | | | H→L+1 | 7.9 |
| | | | | | H→L+5 | 4.4 |
| | $S_0 \rightarrow S_3$ | 3.62 | 343 | 0.3487 | H-5→L | 11.1 |
| | | | | | H-4→L+1 | 2.4 |
| | | | | | H-1→L+4 | 3.3 |
| | | | | | H→L+1 | 61.5 |
| | | | | | H→L+4 | 3.1 |
| | | | | | H→L+5 | 6.9 |

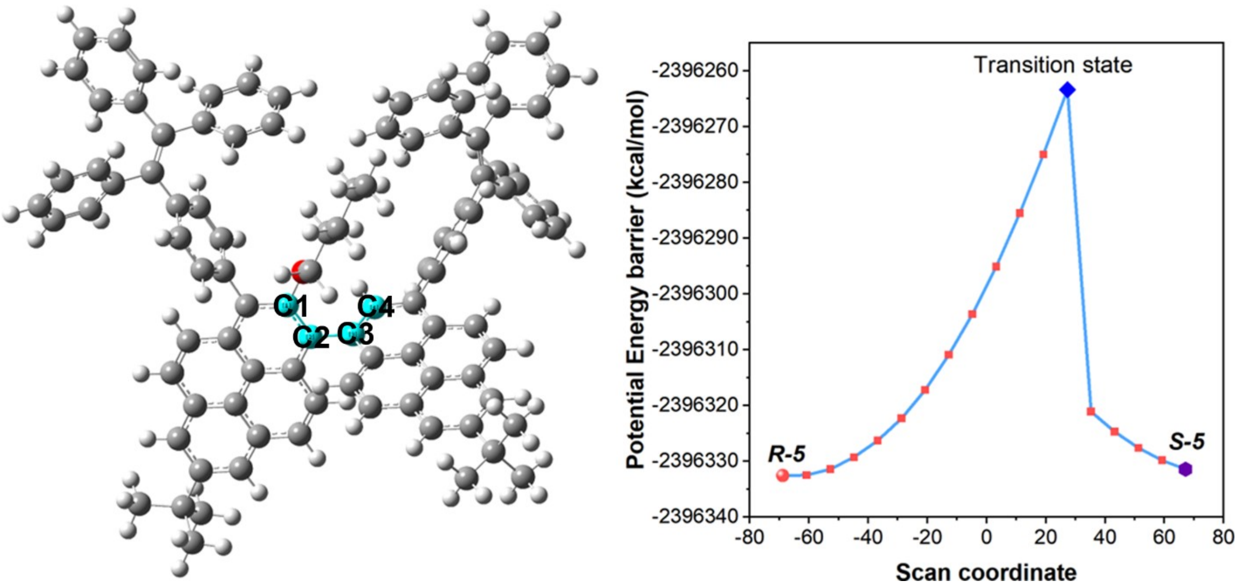


**Figure S27** (Left): the molecular conformation, and (Right )the energy profile for ***R-5*** ↔ **S-5**, transition state conformational interconversion and the associated energy barrier along the C1-C4 (blue sky atom).

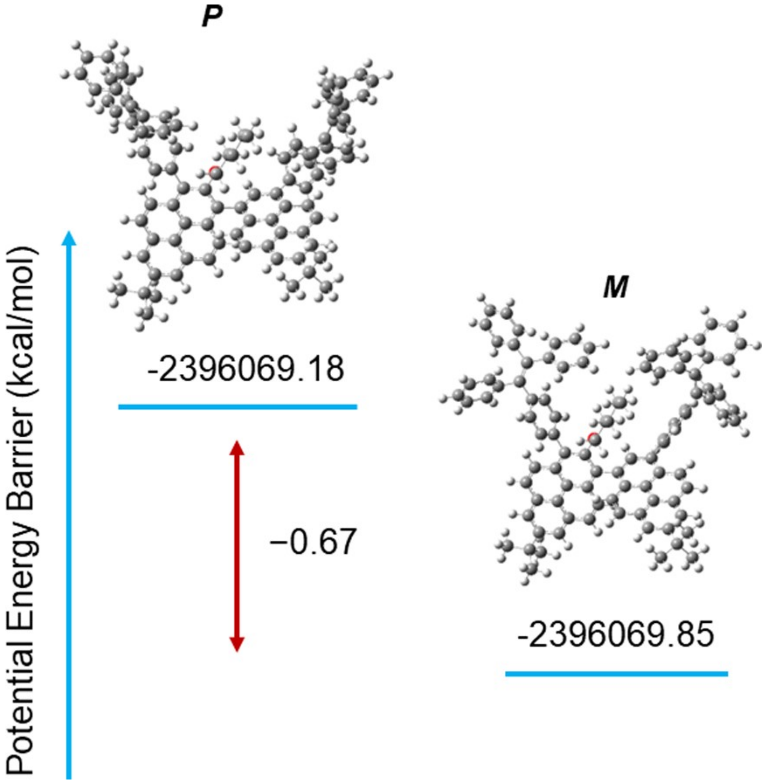


**Figure S28** The energy barrier between plus (*P*) to minus (*M*) helical configurations of ***R-5*** CAM-B3LYP/6-311G(d,p).

## 6. Photophysical Properties

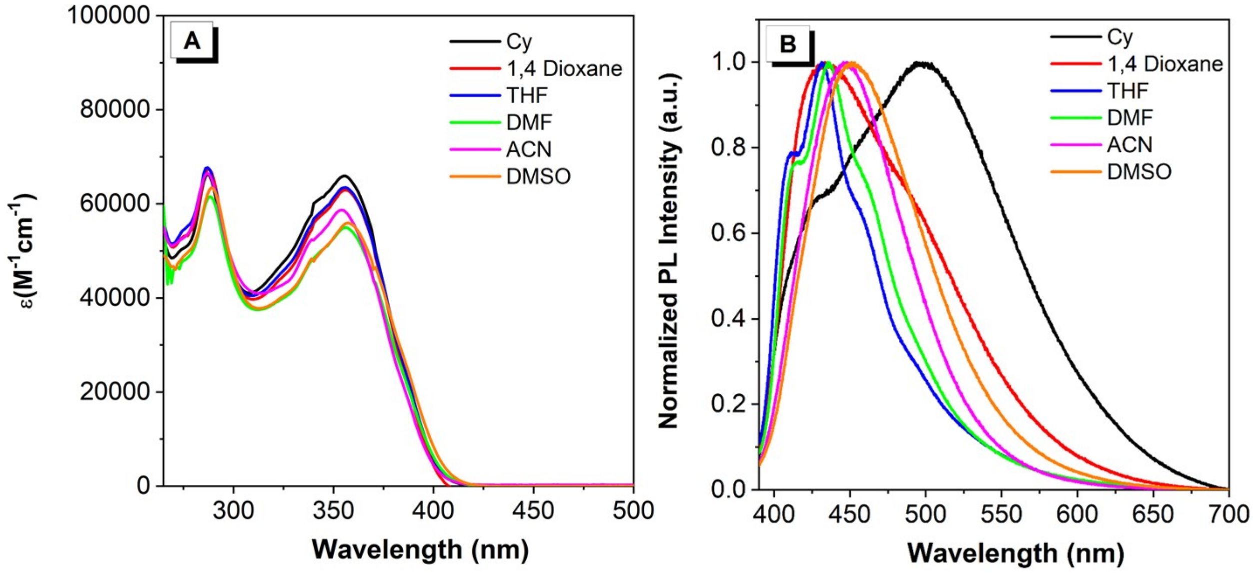


**Figure S29**. (A) UV-vis spectra and (B) Fluorescence spectra of the compound ***R*-5** recorded in six solvents at ~$10^{-5}$ M and 25 °C.

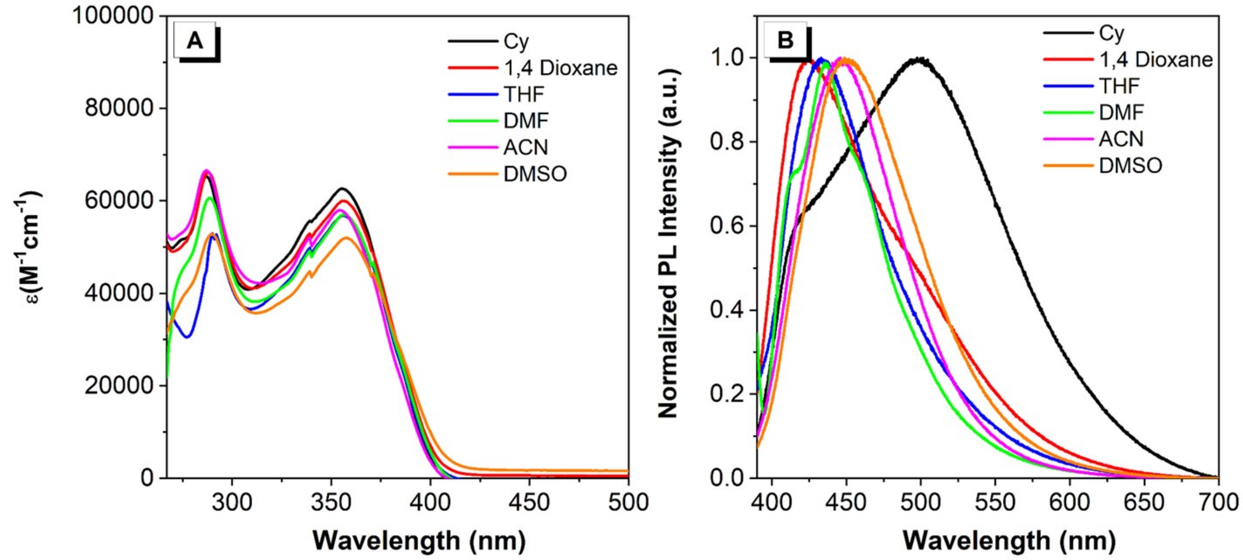


**Figure S30.** (A) UV-vis spectra and (B) Fluorescence spectra of the compound ***S*-5** recorded in six solvents at ~$10^{-5}$ M and 25 °C.

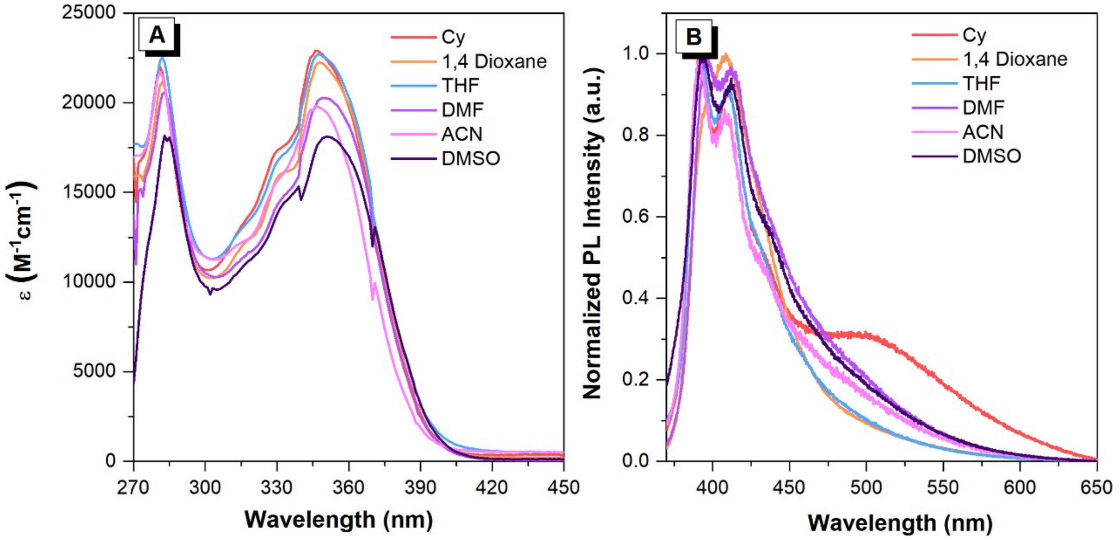


**Figure S31.** (A) UV-vis spectra and (B) Fluorescence spectra of the compound **Py-TPE** recorded in six solvents at ~$10^{-5}$ M and 25 °C.

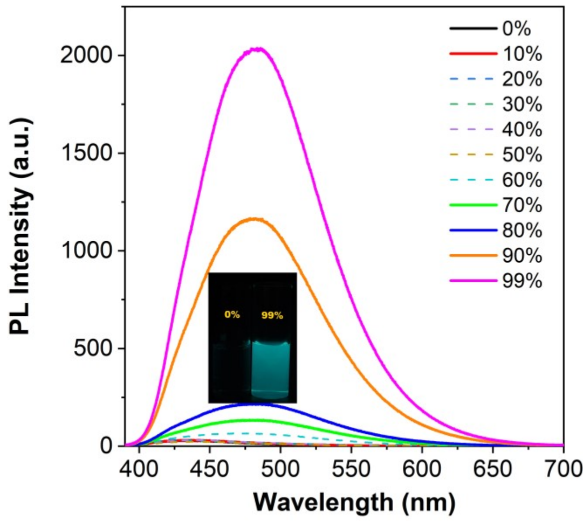


**Figure S32**. PL spectra of ***R*-5** in THF/water mixtures with different water fractions ($f_w$) (~$10^{-5}$ m), Insert: Fluorescence images of compound ***R*-5** in $f_w$ = 0% and 99% under 365 nm irradiation.

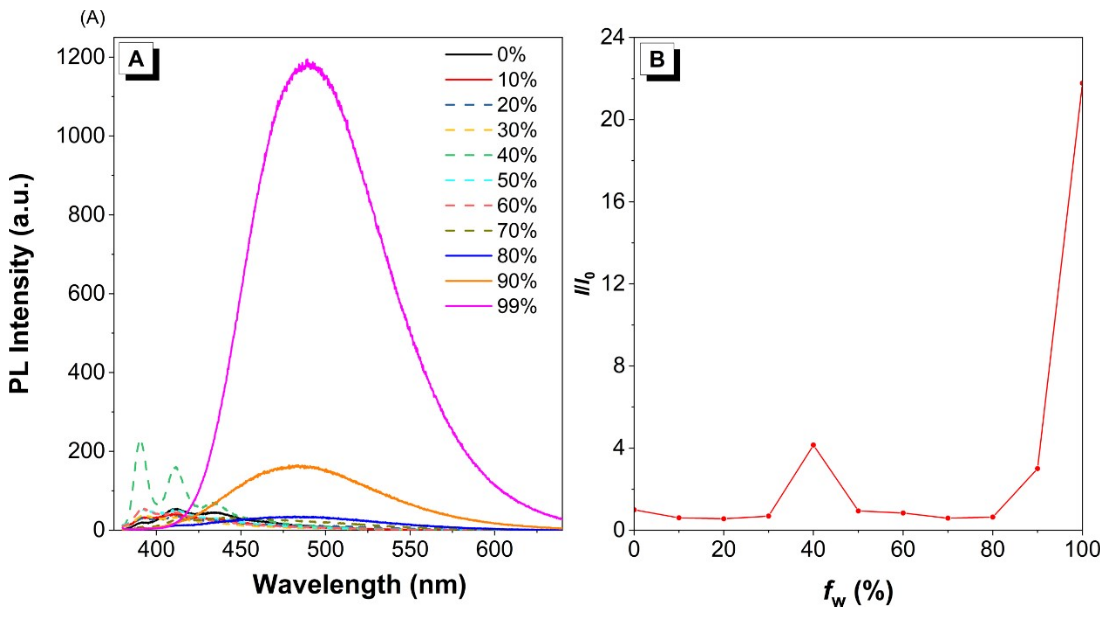


**Figure S33**. (A) PL spectra of **Py-TPE** in THF/water mixtures with different water fractions ($f_w$) (≈$10^{-5}$ m); (B) The plot of relative PL intensity $I/I_0$.

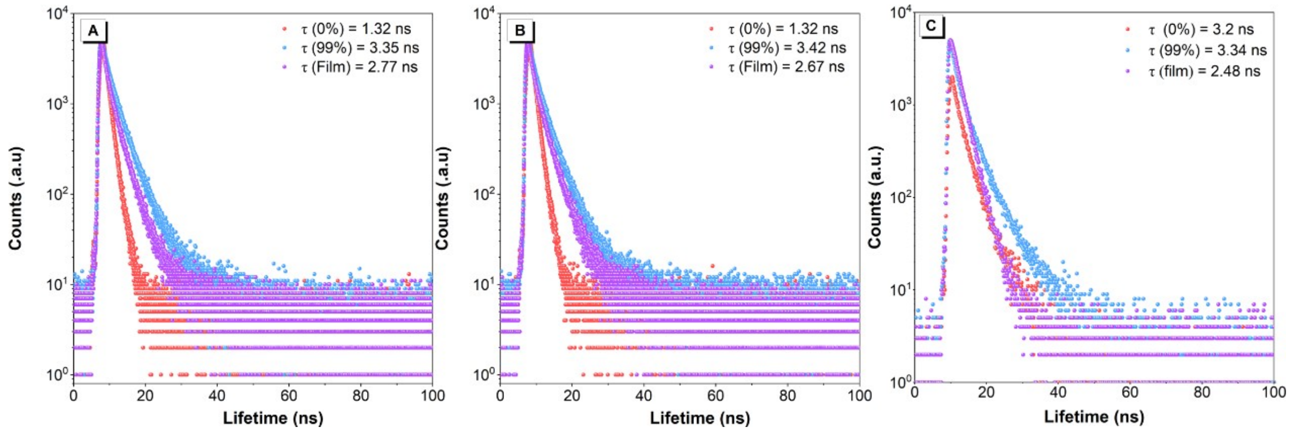


**Figure S34.** Time-resolved fluorescence decay profiles of (A) ***R*-5,** (B) ***S*-5** and (C) **Py-TPE** in THF solution, in $f_w$ = 99%, and in the solid-state.

## 7. Circular Dichroism Spectroscopy

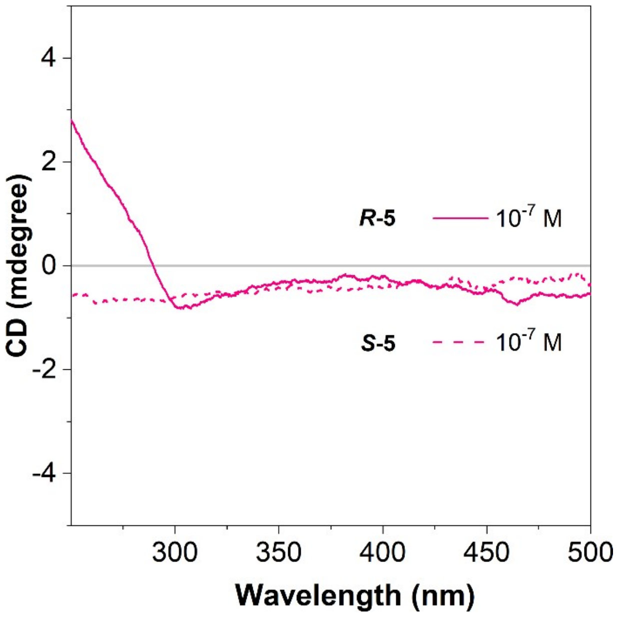


**Figure S35**. CD spectra of ***R-/S*-5** in THF solution of *ca*. $10^{-7}$ M.

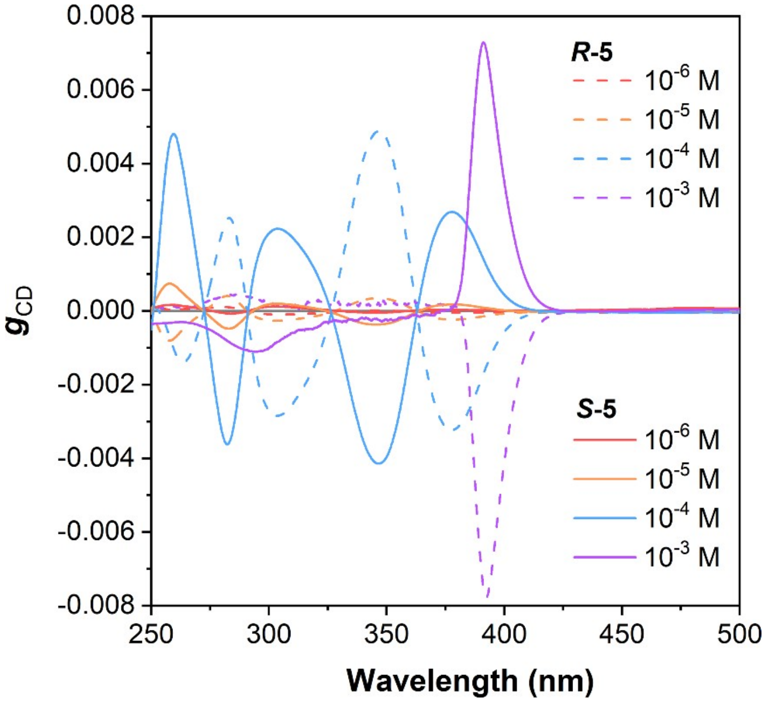


**Figure S36.** The $g_{CD}$ value of concentration-dependent CD spectra of ***R-/S*-5** in THF solution ($10^{-6}$~$10^{-3}$ M).

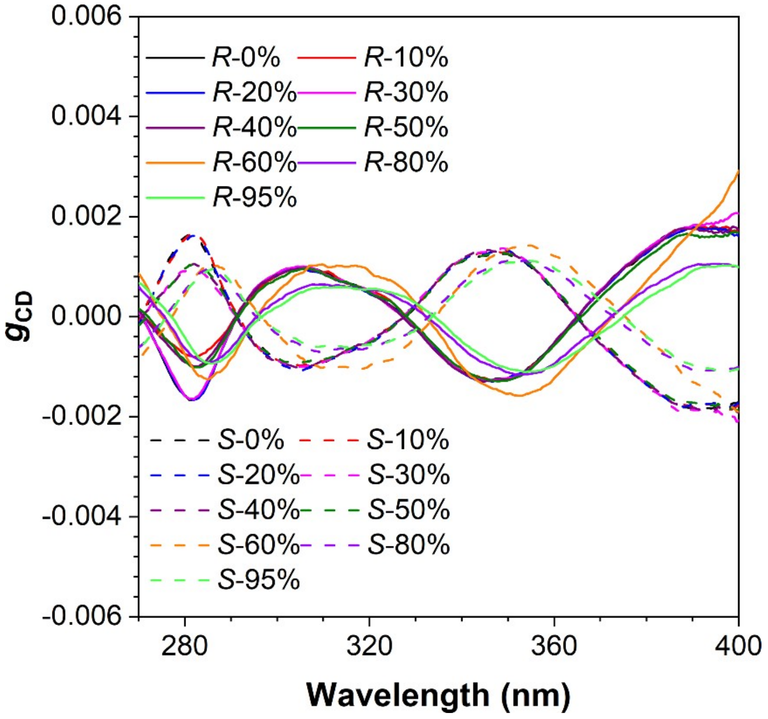


**Figure S37.** The $g_{CD}$ value of compounds ***R*-/*S*-5** in THF and in THF/$H_2O$ mixtures with various water fractions ($f_w$) (10 $\mu$M).

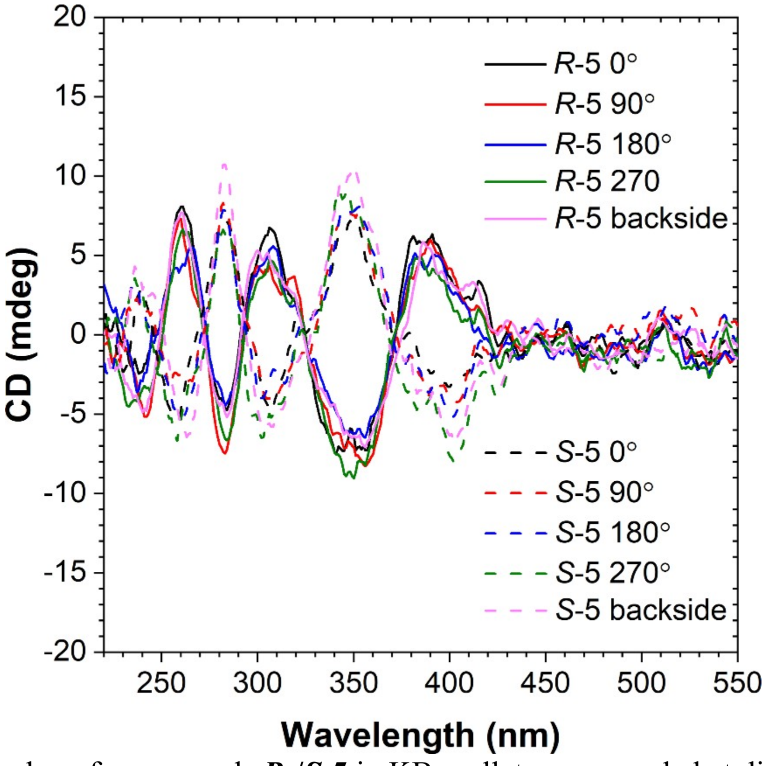


**Figure S38.** The $g_{CD}$ value of compounds ***R*-/*S*-5** in KBr pellet was recorded at different rotation angles in the plane perpendicular to the light axis.

## 8. Circularly Polarized Luminescence Spectroscopy

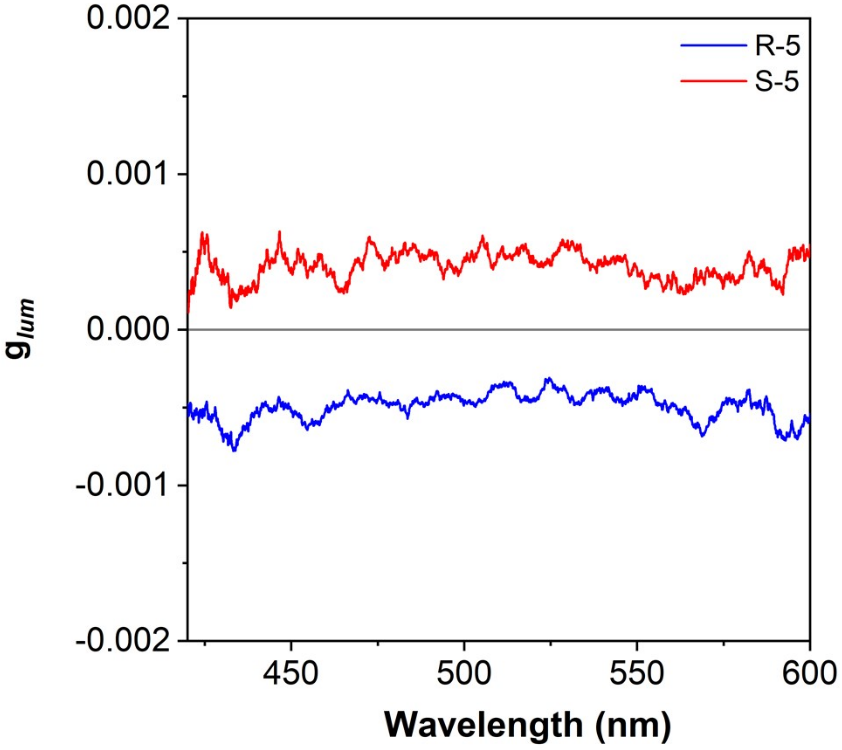


**Figure S39**. $g_{\text{lum}}$ of ***R*/*S*-5** in a KBr pellet.

**Table S5**. Summary of chiroptical properties in pyrene-based chiral molecules

| Compound | $\Phi_{fl}$ | $\lvert g_{abs}\rvert$ | $\lvert g_{lum}\rvert$ | Ref. |
|---|---|---|---|---|
| | Solution /solid state | | | |
| 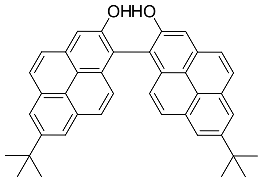 | 0.57/nd | $3.6\times10^{-4}$/nd | $1.2\times10^{-3}$/nd | Ref.4 |
| 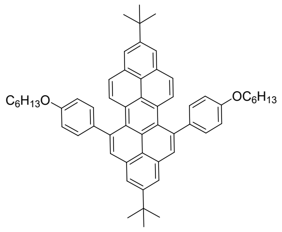 | nd | $1.2\times10^{-3}$/nd | $7.7\times10^{-4}$/nd | Ref.5 |
| 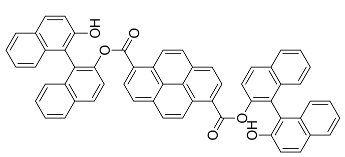 | nd | nd | $6.9\times10^{-3}$/nd | Ref.6 |
| 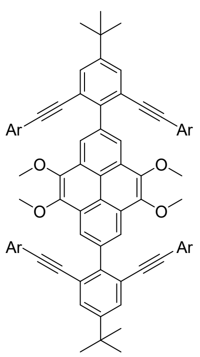 | nd | $1.0\times10^{-3}$/nd | nd | Ref.7 |
| 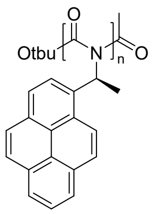 | nd | nd | nd/$1.9\times10^{-2}$ | Ref.8 |
| 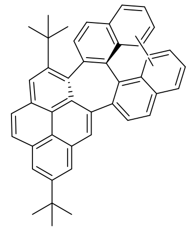 | 0.3/nd | $2.3\times10^{-3}$/nd | nd | Ref.9 |
| 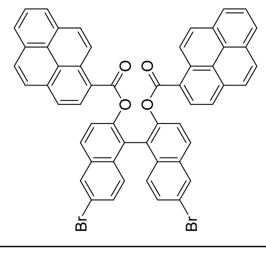 | nd/55.9 | nd | nd/$4.3\times10^{-3}$ | Ref.10 |

| | | | | |
|---|---|---|---|---|
| 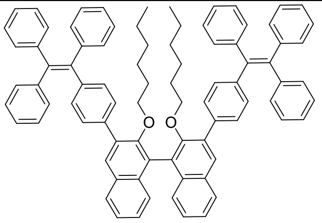 | 0.2/0.28 | nd | nd | Ref.11 |
| 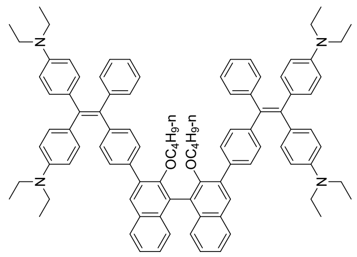 | nd/0.40 | $3\times10^{-4}/7.8\times10^{-4}$ | nd/$3.6\times10^{-3}$ | Ref.12 |
| 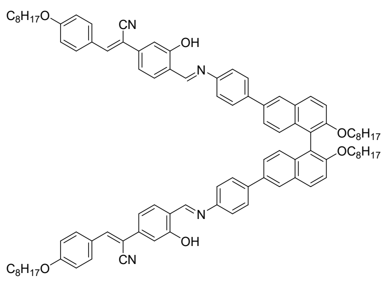 | 0.12/nd | $1.27\times10^{-3}$/nd | $1.7\times10^{-4}$/nd | Ref.13 |
| 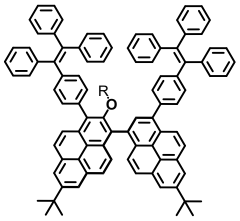 R = n-pentyl | < 0.01 / **0.66** | $4.12\times10^{-3}/6.48\times10^{-4}$ | Non / $4.68\times10^{-4}$ | **This work** |

Notes: nd: no report. Non: no detect.

## 9. Scanning Electron Microscope Analysis

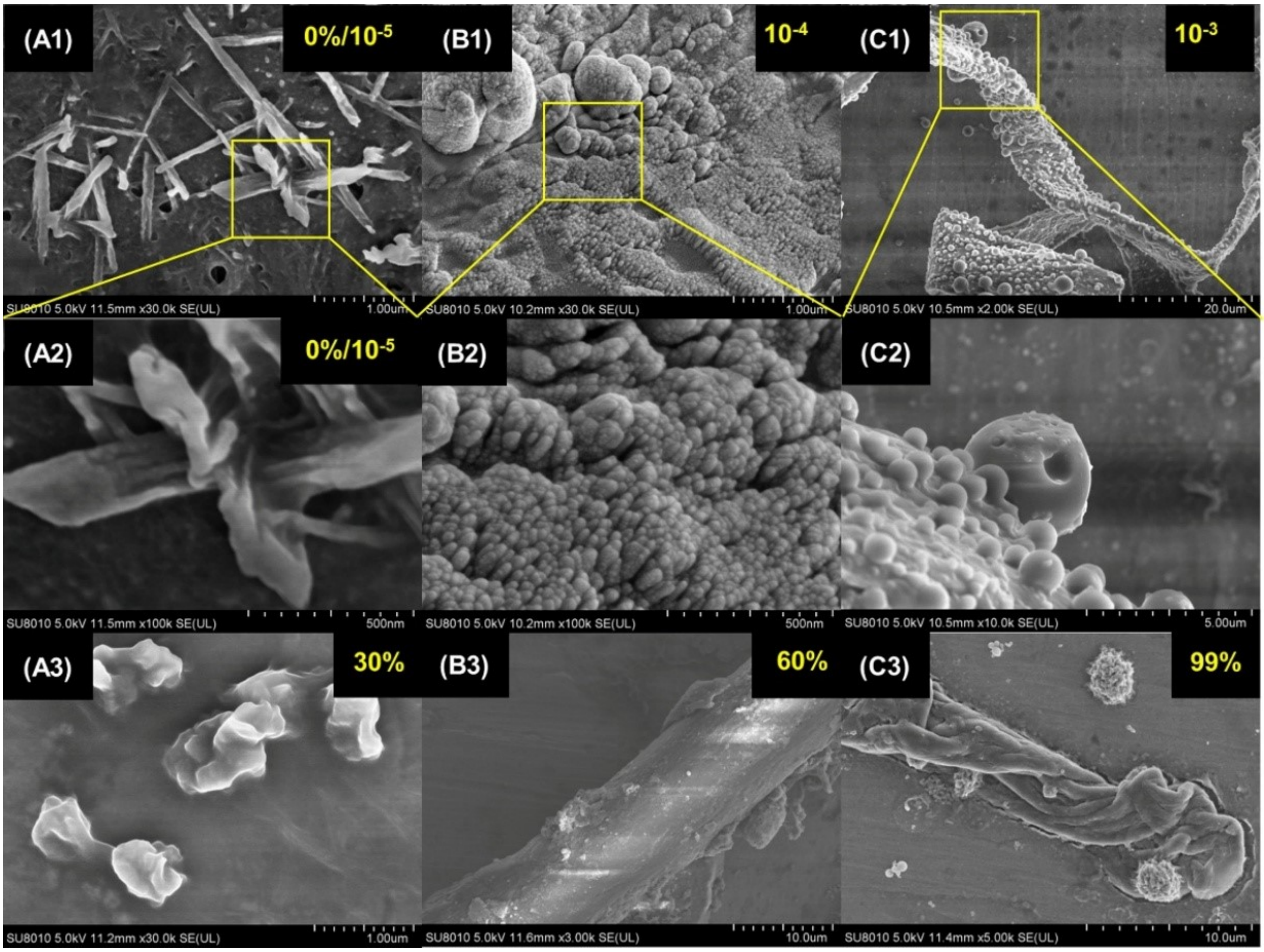


**Figure S40**. SEM of ***R*-5** (A1) in $10^{-5}$ M (in $f_w$ = 0%), (B1) in $10^{-4}$ M and (C1) in $10^{-3}$ M, respectively, and (A2-C2) the zoom of the selected region. (A3-C3) SEM of ***R*-5** in $f_w$ = 30%, 60% and 99% (10 $\mu$M).

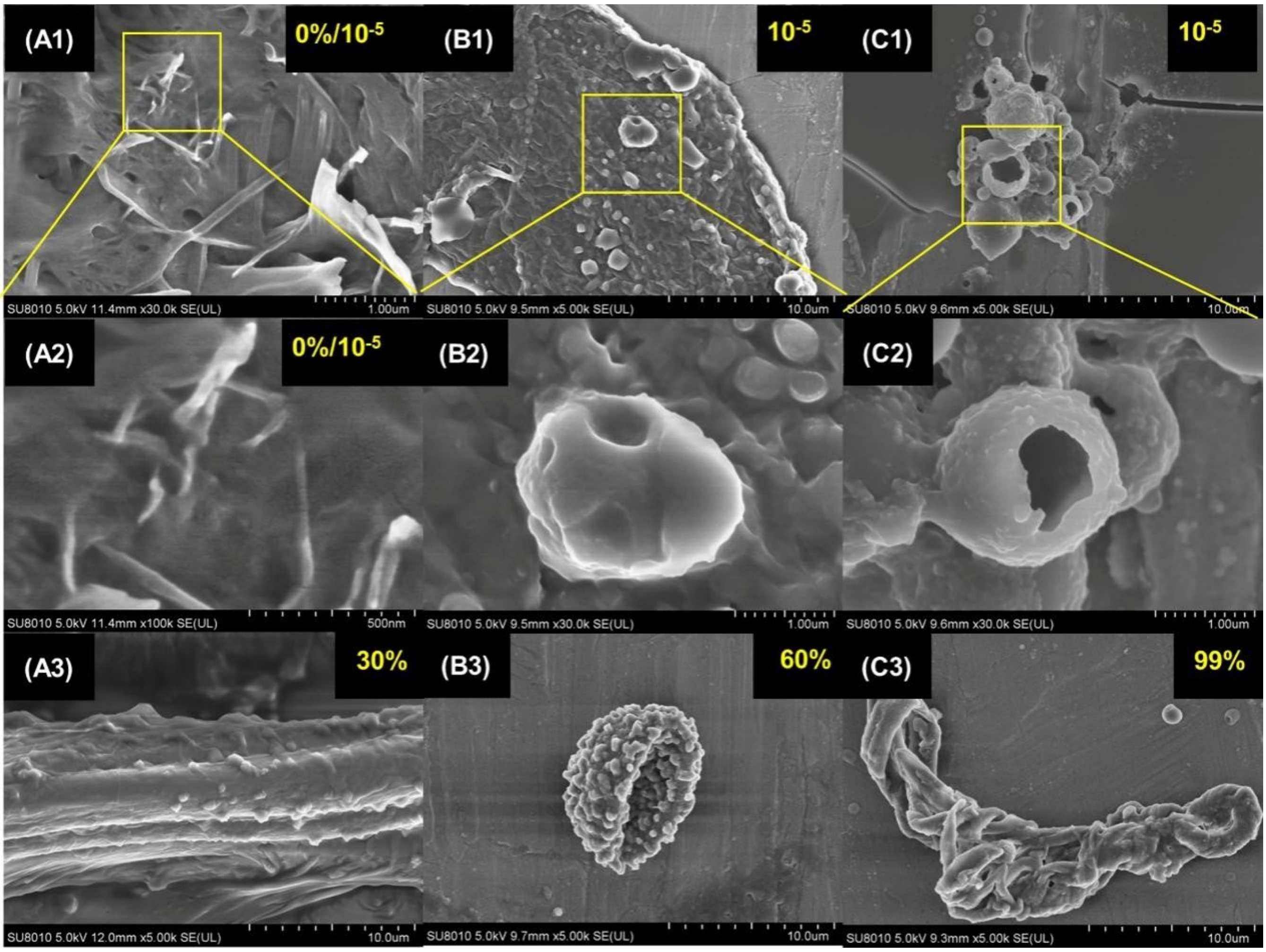


**Figure S41**. SEM of ***S*-5** (A1) in $10^{-5}$ M (in $f_w$ = 0%), (B1) in $10^{-4}$ M and (C1) in $10^{-3}$ M, respectively, and (A2-C2) the zoom of the selected region. (A3-C3) SEM of ***S*-5** in $f_w$ = 30%, 60% and 99% (10 $\mu$M).

# 10. Femtosecond Transient Absorption Spectroscopy Analysis

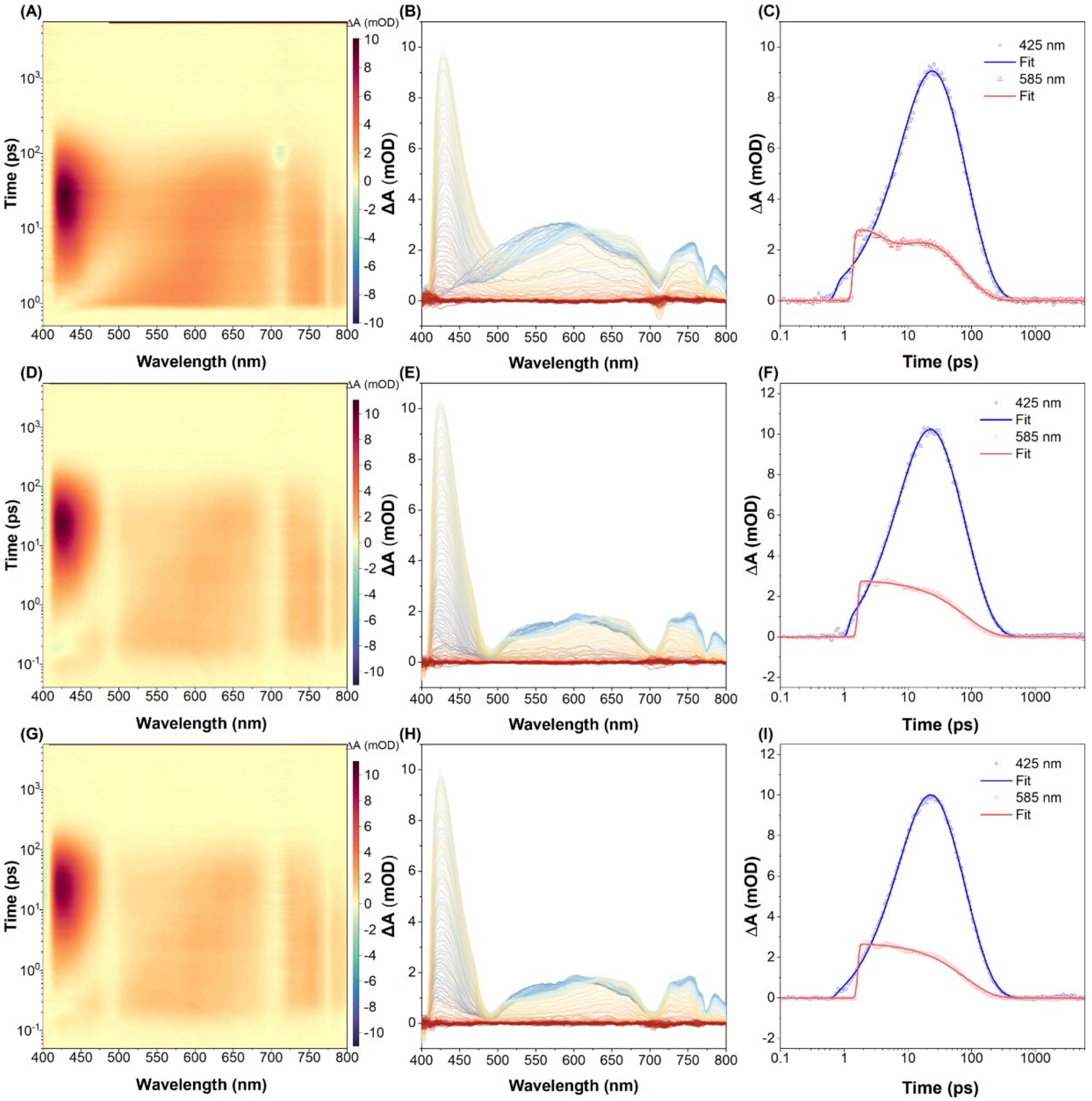


**Figure S42.** Femtosecond transient absorption spectroscopy analysis. (A, D and G) Pseudocolor 2D plots of TA data; (B, E, and H) TA spectra at various time delays and TA traces (C, F, and I) at 425 nm and 585 nm probe wavelengths of ***R*-5** in THF solution**.** Among them, the excitation wavelength is linear polarization for (A, B, and C), left circularly polarized (LCP) for (D, E, and F), and right circularly polarized (RCP) (G, H, respectively.

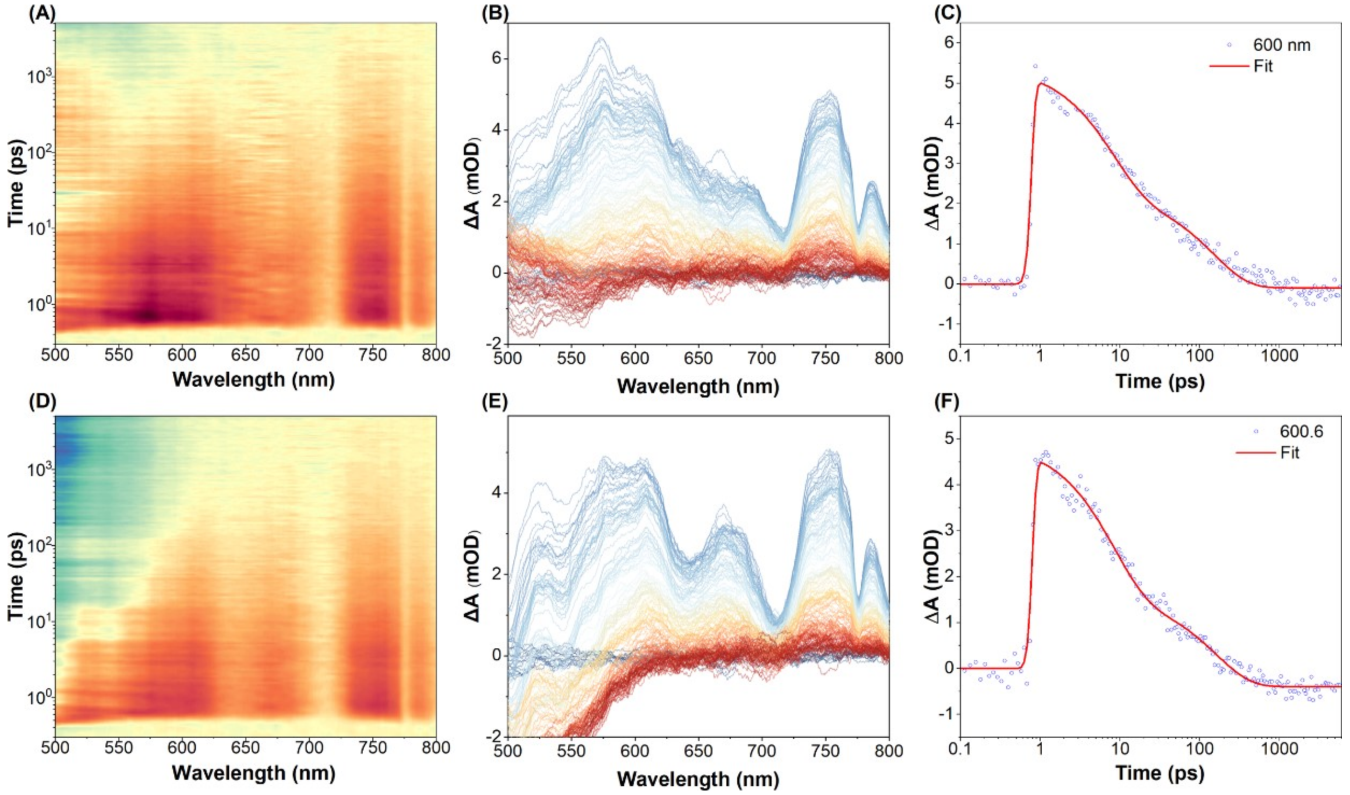


**Figure S43**. (A and D) Pseudocolor 2D plots of TA data; (B and E) TA spectra at various time delays and TA traces (C and F) at 600 nm probe wavelengths of ***R*-5** in solid state. Among them, the excitation wavelength is left circularly polarized (LCP) for (A, B, and C), and right circularly polarized (RCP) (D, E, and F) under 350 nm excitation.

**Table S6.** TA kinetics fitting results for compounds ***R*-/*S*-5** (fitted from Fig. 4A-4C and S39A-C) in THF solution.

| Compound | $\lambda_{probe}$ (nm) | $\iota$ (ps) | $A_1$ | $\iota_2$ (ps) | $A_2$ | $\iota_3$ (ps) | $A_3$ | $c^2$ |
|---|---|---|---|---|---|---|---|---|
| R-5[a)] | 425 | 10±2 | -0.95 | 82±10 | 1 | | | 0.9843 |
| | 585 | 2±0.3 | 0.26 | 11±1 | -0.05 | 71±2 | 0.74 | 0.9581 |
| | 750 | 69±1 | 1 | | | | | 0.9933 |
| S-5[a)] | 425 | 11±1 | -0.94 | 78±2 | 1 | | | 0.9988 |
| | 585 | 2±0.3 | 0.20 | 9±1 | -0.05 | 68±2 | 0.80 | 0.9416 |
| | 750 | 66±1 | 1 | | | | | 0.9956 |

[a)] probed using linear polarization at 350 nm excitation

**Table S7**. TA kinetics fitting results for compounds ***R*-/*S*-5** (fitted from Fig. 4D-4H and S39D-I) in THF solution.

| Compound | $\lambda_{probe}$ (nm) | $\iota$ (ps) | $A_1$ | $\iota_2$ (ps) | $A_2$ | $\iota_3$ (ps) | $A_3$ | $c^2$ |
|---|---|---|---|---|---|---|---|---|
| ***R*-5**[a)] | 425 | 8.9±0.1 | -0.94 | 79±1 | 1 | | | 0.9991 |
| | 585 | 80±1 | 1 | | | | | 0.9971 |
| ***R*-5**[b)] | 425 | 9.1±0.1 | -0.96 | 78±1 | 1 | | | 0.9992 |
| | 585 | 80±1 | 1 | | | | | 0.997 |
| ***R*-5**[c)] | 485 | 0.6±0.1 | 1 | | | | | 0.8521 |
| ***S*-5**[a)] | 425 | 9.4±0.1 | -0.94 | 84±1 | 1 | | | 0.9989 |
| | 585 | 84±2 | 1 | | | | | 0.9934 |
| | 750 | 0.7±0.2 | -0.1 | 72±1 | 1 | | | 0.9951 |
| ***S*-5**[b)] | 425 | 9.2±0.1 | -0.93 | 84±1 | 1 | | | 0.9987 |
| | 585 | 83±2 | 1 | | | | | 0.9914 |
| | 750 | 0.8±0.2 | -0.1 | 72±1 | 1 | | | 0.9958 |
| ***S*-5**[c)] | 440 | 0.11±0.03 | 1 | | | | | 0.8901 |
| | 450 | 0.12±0.02 | 1 | | | | | 0.8223 |
| | 465 | 0.13±0.04 | 1 | | | | | 0.7613 |
| | 565 | 0.15±0.02 | 1 | | | | | 0.8118 |

[a)] probed using left polarization at 350 nm excitation, [b)] probed using right polarization at 350 nm excitation. c) fitting results from the transient dynamics of differential TA spectra (H) at different wavelengths in THF solution.

**Table S8**. TA kinetics fitting results for compounds ***R*-/*S*-5** (fitted from Fig. 5 and S40) in thin film.

| Compound | $\lambda_{probe}$ (nm) | ι (ps) | $A_1$ | $ι_2$ (ps) | $A_2$ | $ι_3$ (ps) | $A_3$ | $c^2$ |
|---|---|---|---|---|---|---|---|---|
| ***R*-5**[a)] | 600 | 8±1 | 0.55 | 148±12 | 0.45 | | | 0.978 |
| ***R*-5**[b)] | 600 | 8±0.6 | 0.33 | 159±16 | 0.67 | | | 0.983 |
| ***R*-5**[c)] | 630 | 60±12 | 1 | | | | | 0.691 |
| | 675 | 50±10 | 1 | | | | | 0.7501 |
| ***S*-5**[a)] | 540 | 3±0.5 | 0.51 | 45±3 | 0.49 | | | 0.982 |
| ***S*-5**[b)] | 540 | 3±0.5 | 0.55 | 52±3 | 0.45 | | | 0.981 |
| ***S*-5**[c)] | 525 | 25±1 | 1 | | | | | 0.8978 |
| | 615 | 30±4 | 1 | | | | | 0.7481 |

[a)] probed using left polarization at 350 nm excitation. [b)] probed using right polarization at 350 nm excitation. c) fitting results from the transient dynamics of differential TA spectra (H) at different wavelengths in film state.

**Table S9.** Atom coordinates and absolute energies for ***R*-5** Standard orientation

| AtomicType | Coordinates(Angstroms) | | |
|---|---|---|---|
| | X | Y | Z |
| C | 2.73639000 | 1.41598000 | -0.60923500 |
| C | 3.23273000 | 2.44670200 | -1.42225100 |
| C | 2.38661300 | 3.53051000 | -1.76537000 |
| C | 1.05275300 | 3.57973900 | -1.28623900 |
| C | 0.57757300 | 2.55558400 | -0.45158100 |
| C | 1.42350300 | 1.48359900 | -0.14769800 |
| C | 2.87792000 | 4.57117000 | -2.60852600 |
| C | 2.04359600 | 5.65251500 | -2.97825200 |
| C | 0.69105400 | 5.66203700 | -2.49625200 |
| C | 0.22142200 | 4.68231600 | -1.69888100 |
| C | 4.20390800 | 4.53932300 | -3.09233800 |
| C | 4.66600700 | 5.57243900 | -3.91201200 |
| C | 3.85766000 | 6.64199500 | -4.27687100 |
| C | 2.54729800 | 6.65770700 | -3.79774300 |
| C | 4.57565000 | 2.43579000 | -1.93868700 |
| C | 5.03573200 | 3.42988800 | -2.72599100 |
| C | 4.34899200 | 7.78304000 | -5.17517000 |
| C | 5.80772900 | 7.59811800 | -5.60517100 |
| C | 4.23948000 | 9.11533100 | -4.41458700 |
| C | 3.48065900 | 7.84604400 | -6.44303000 |
| O | 0.94062600 | 0.43266500 | 0.58854200 |
| C | 0.98977000 | 0.58900400 | 2.00791300 |
| C | -0.81796100 | 2.55710400 | 0.07577500 |
| C | -1.25870500 | 3.49646000 | 1.02368200 |
| C | -2.61043600 | 3.45780400 | 1.45614400 |
| C | -3.50168300 | 2.46638800 | 0.96792100 |
| C | -3.01729600 | 1.49713200 | 0.07164800 |
| C | -1.69843500 | 1.57797300 | -0.36324300 |
| C | -3.07621400 | 4.42662100 | 2.39623000 |
| C | -4.42715500 | 4.43029400 | 2.81842100 |
| C | -5.31460400 | 3.44670800 | 2.26626400 |
| C | -4.87673500 | 2.51817100 | 1.39253600 |

| | | | |
|---|---|---|---|
| C | -2.19836300 | 5.39915700 | 2.92391700 |
| C | -2.67416300 | 6.33382100 | 3.84691100 |
| C | -3.99838700 | 6.34638700 | 4.26802900 |
| C | -4.85661700 | 5.38387700 | 3.73675700 |
| C | -0.38644500 | 4.49065200 | 1.58974100 |
| C | -0.83065400 | 5.38918200 | 2.49214100 |
| C | -4.53628100 | 7.36598100 | 5.27866900 |
| C | -3.45446600 | 8.34168300 | 5.75263900 |
| C | -5.08486600 | 6.62480800 | 6.50959500 |
| C | -5.66605600 | 8.18260000 | 4.62889100 |
| C | 3.58159800 | 0.24057600 | -0.24974000 |
| C | -3.84393600 | 0.36150600 | -0.41310000 |
| C | -4.46234200 | -0.51564200 | 0.47836900 |
| C | -5.15795600 | -1.62246400 | 0.02331800 |
| C | -5.26233900 | -1.89503700 | -1.34014100 |
| C | -4.67509400 | -0.99837000 | -2.23276600 |
| C | -3.96738500 | 0.10156200 | -1.77765700 |
| C | -5.99629600 | -3.09144700 | -1.84364700 |
| C | -5.77569700 | -4.33415200 | -1.36451200 |
| C | -6.63263800 | -5.50186200 | -1.72297400 |
| C | -4.65645300 | -4.64982000 | -0.42860700 |
| C | -6.98438700 | -2.80636200 | -2.92497700 |
| C | 3.46619300 | -0.95789400 | -0.94914300 |
| C | 4.27184300 | -2.04159500 | -0.64237600 |
| C | 5.21692200 | -1.96834900 | 0.38164900 |
| C | 5.33489000 | -0.76366100 | 1.07551400 |
| C | 4.52717400 | 0.32045200 | 0.76904200 |
| C | 6.12837100 | -3.10656000 | 0.69641000 |
| C | 5.71072600 | -4.38357500 | 0.82274500 |
| C | 7.56209900 | -2.72068700 | 0.86011700 |
| C | 6.65927900 | -5.53013300 | 0.94573300 |
| C | 4.27121500 | -4.77542900 | 0.84959000 |
| C | 8.27634000 | -3.06228800 | 2.00743700 |
| C | 9.60058000 | -2.67947700 | 2.15948100 |
| C | 10.23564200 | -1.95094900 | 1.16429600 |

| | | | |
|---|---|---|---|
| C | 9.53263300 | -1.59670300 | 0.02071900 |
| C | 8.20530700 | -1.96834800 | -0.12365200 |
| C | 3.81274300 | -5.80317600 | 0.02341500 |
| C | 2.48375800 | -6.19480100 | 0.04603900 |
| C | 1.59142000 | -5.58178700 | 0.91488400 |
| C | 2.03955300 | -4.57372000 | 1.75694000 |
| C | 3.36691600 | -4.17262500 | 1.72311600 |
| C | 6.49978700 | -6.46594300 | 1.96884600 |
| C | 7.36519100 | -7.54180700 | 2.08911600 |
| C | 8.39325000 | -7.71583700 | 1.17270900 |
| C | 8.54751200 | -6.80575100 | 0.13697000 |
| C | 7.68943900 | -5.72225100 | 0.02577800 |
| C | -6.97040000 | -3.51606200 | -4.12530200 |
| C | -7.87749400 | -3.22470200 | -5.13259700 |
| C | -8.81772600 | -2.21962200 | -4.95701500 |
| C | -8.83552300 | -1.49858900 | -3.77097700 |
| C | -7.91971600 | -1.78215900 | -2.77000600 |
| C | -6.04959500 | -6.69695600 | -2.14722000 |
| C | -6.83259700 | -7.79322500 | -2.47295600 |
| C | -8.21412800 | -7.72265100 | -2.35758300 |
| C | -8.80437100 | -6.54789600 | -1.91415400 |
| C | -8.02087300 | -5.44762500 | -1.60131100 |
| C | -4.90347200 | -5.36627100 | 0.74376800 |
| C | -3.87620800 | -5.66009700 | 1.62639200 |
| C | -2.57699600 | -5.26223200 | 1.34123400 |
| C | -2.31299000 | -4.57447600 | 0.16508700 |
| C | -3.34418700 | -4.27195800 | -0.71136600 |
| H | 0.04351600 | 6.47935300 | -2.79383700 |
| H | -0.80444000 | 4.70961100 | -1.35802900 |
| H | 5.68795500 | 5.51490600 | -4.26264300 |
| H | 1.88391700 | 7.47258400 | -4.06441100 |
| H | 5.21829000 | 1.60452900 | -1.68159500 |
| H | 6.05225500 | 3.40149900 | -3.10270100 |
| H | 6.10954700 | 8.43401000 | -6.23995300 |
| H | 5.94812300 | 6.67998600 | -6.17989400 |

| | | | |
|---|---|---|---|
| H | 6.48371500 | 7.57413000 | -4.74756900 |
| H | 4.58656700 | 9.93971000 | -5.04315600 |
| H | 3.21113300 | 9.33088400 | -4.12008700 |
| H | 4.84957900 | 9.09731600 | -3.50876000 |
| H | 3.54160100 | 6.91028200 | -7.00307100 |
| H | 3.81992600 | 8.65595000 | -7.09426400 |
| H | 2.43059400 | 8.02591300 | -6.20709300 |
| H | 0.41220700 | 1.47177900 | 2.30238000 |
| H | 2.03006800 | 0.73775500 | 2.31974300 |
| H | -1.33148100 | 0.82573000 | -1.04961300 |
| H | -6.35750600 | 3.46909900 | 2.56252600 |
| H | -5.57011100 | 1.79798100 | 0.98063700 |
| H | -1.97113000 | 7.06090600 | 4.23113800 |
| H | -5.89777200 | 5.36250400 | 4.03793600 |
| H | 0.64913400 | 4.50450600 | 1.27691800 |
| H | -0.15294700 | 6.12665300 | 2.90766600 |
| H | -3.88569700 | 9.04448300 | 6.46876300 |
| H | -3.04277700 | 8.92434900 | 4.92561300 |
| H | -2.63220100 | 7.82458700 | 6.25211100 |
| H | -5.47234000 | 7.33977100 | 7.24023000 |
| H | -5.89667100 | 5.94515700 | 6.24550600 |
| H | -4.30032200 | 6.03698900 | 6.99126900 |
| H | -5.30179900 | 8.72045900 | 3.75080800 |
| H | -6.05969600 | 8.91518600 | 5.33845800 |
| H | -6.49470700 | 7.54761100 | 4.31137500 |
| H | -4.37583700 | -0.34120400 | 1.54411900 |
| H | -5.61257100 | -2.29785200 | 0.73691200 |
| H | -4.76731700 | -1.17103400 | -3.29855400 |
| H | -3.50862000 | 0.77660000 | -2.49061400 |
| H | 2.74068600 | -1.03690900 | -1.74963900 |
| H | 4.16986700 | -2.95863800 | -1.20833000 |
| H | 6.07556800 | -0.67187400 | 1.86086800 |
| H | 4.64048600 | 1.24759500 | 1.31934400 |
| H | 7.78737000 | -3.63626700 | 2.78457700 |
| H | 10.13793000 | -2.95264400 | 3.05990400 |

| | | | |
|---|---|---|---|
| H | 11.27134200 | -1.65510700 | 1.28123200 |
| H | 10.01894500 | -1.02399700 | -0.76022200 |
| H | 7.65693500 | -1.67749800 | -1.01209500 |
| H | 4.50899300 | -6.29746300 | -0.64377900 |
| H | 2.14446400 | -6.98628700 | -0.61163500 |
| H | 0.55462900 | -5.89536900 | 0.93884500 |
| H | 1.35209700 | -4.09609300 | 2.44497800 |
| H | 3.70999100 | -3.38249400 | 2.37947500 |
| H | 5.68889200 | -6.34453300 | 2.67746300 |
| H | 7.23262600 | -8.25056200 | 2.89806700 |
| H | 9.06596900 | -8.56048900 | 1.26162300 |
| H | 9.34002100 | -6.93888400 | -0.58990800 |
| H | 7.81804600 | -5.01319400 | -0.78222200 |
| H | -6.24161800 | -4.30421300 | -4.26662700 |
| H | -7.84829600 | -3.78560300 | -6.05923300 |
| H | -9.52841300 | -1.99401000 | -5.74308200 |
| H | -9.56197600 | -0.70751800 | -3.62687600 |
| H | -7.92797900 | -1.20558500 | -1.85244600 |
| H | -4.97066200 | -6.76303600 | -2.22392500 |
| H | -6.36176500 | -8.70821500 | -2.81268000 |
| H | -8.82667400 | -8.58134400 | -2.60524200 |
| H | -9.88119700 | -6.48640100 | -1.81016900 |
| H | -8.48780600 | -4.53239600 | -1.25986700 |
| H | -5.91386600 | -5.68932900 | 0.96534900 |
| H | -4.09014200 | -6.20416200 | 2.53881100 |
| H | -1.77418500 | -5.49168000 | 2.03218900 |
| H | -1.30065000 | -4.26823600 | -0.07073500 |
| H | -3.13321700 | -3.72876700 | -1.62385700 |
| C | 0.41489900 | -0.66301100 | 2.63245300 |
| H | 1.00311300 | -1.51964000 | 2.29030200 |
| H | -0.60091500 | -0.80458500 | 2.25173700 |
| C | 0.40046700 | -0.60186400 | 4.15653000 |
| H | -0.19333500 | 0.25999800 | 4.48244600 |
| H | 1.41755300 | -0.43008400 | 4.52875300 |
| C | -0.15988000 | -1.86596500 | 4.80262600 |

| | | | |
|---|---|---|---|
| H | 0.44521400 | -2.72578000 | 4.49462700 |
| H | -1.16836600 | -2.04983500 | 4.41751100 |
| C | -0.19840900 | -1.79121700 | 6.32499400 |
| H | 0.80239300 | -1.63767300 | 6.73743500 |
| H | -0.60012400 | -2.70818100 | 6.76124300 |
| H | -0.82461700 | -0.96089300 | 6.66175900 |

Total Energy (CAM-RB3LYP) = -3818.515304 Hartree

**Table S10.** Atom coordinates and absolute energies for ***S*-5** Standard orientation

| AtomicType | Coordinates(Angstroms) | | |
|---|---|---|---|
| | X | Y | Z |
| C | -2.73639000 | 1.41598000 | -0.60923500 |
| C | -3.23273000 | 2.44670200 | -1.42225100 |
| C | -2.38661300 | 3.53051000 | -1.76537000 |
| C | -1.05275300 | 3.57973900 | -1.28623900 |
| C | -0.57757300 | 2.55558400 | -0.45158100 |
| C | -1.42350300 | 1.48359900 | -0.14769800 |
| C | -2.87792000 | 4.57117000 | -2.60852600 |
| C | -2.04359600 | 5.65251500 | -2.97825200 |
| C | -0.69105400 | 5.66203700 | -2.49625200 |
| C | -0.22142200 | 4.68231600 | -1.69888100 |
| C | -4.20390800 | 4.53932300 | -3.09233800 |
| C | -4.66600700 | 5.57243900 | -3.91201200 |
| C | -3.85766000 | 6.64199500 | -4.27687100 |
| C | -2.54729800 | 6.65770700 | -3.79774300 |
| C | -4.57565000 | 2.43579000 | -1.93868700 |
| C | -5.03573200 | 3.42988800 | -2.72599100 |
| C | -4.34899200 | 7.78304000 | -5.17517000 |
| C | -5.80772900 | 7.59811800 | -5.60517100 |
| C | -4.23948000 | 9.11533100 | -4.41458700 |
| C | -3.48065900 | 7.84604400 | -6.44303000 |
| O | -0.94062600 | 0.43266500 | 0.58854200 |
| C | -0.98977000 | 0.58900400 | 2.00791300 |
| C | 0.81796100 | 2.55710400 | 0.07577500 |
| C | 1.25870500 | 3.49646000 | 1.02368200 |
| C | 2.61043600 | 3.45780400 | 1.45614400 |
| C | 3.50168300 | 2.46638800 | 0.96792100 |
| C | 3.01729600 | 1.49713200 | 0.07164800 |
| C | 1.69843500 | 1.57797300 | -0.36324300 |
| C | 3.07621400 | 4.42662100 | 2.39623000 |
| C | 4.42715500 | 4.43029400 | 2.81842100 |
| C | 5.31460400 | 3.44670800 | 2.26626400 |
| C | 4.87673500 | 2.51817100 | 1.39253600 |

| | | | |
|---|---|---|---|
| C | 2.19836300 | 5.39915700 | 2.92391700 |
| C | 2.67416300 | 6.33382100 | 3.84691100 |
| C | 3.99838700 | 6.34638700 | 4.26802900 |
| C | 4.85661700 | 5.38387700 | 3.73675700 |
| C | 0.38644500 | 4.49065200 | 1.58974100 |
| C | 0.83065400 | 5.38918200 | 2.49214100 |
| C | 4.53628100 | 7.36598100 | 5.27866900 |
| C | 3.45446600 | 8.34168300 | 5.75263900 |
| C | 5.08486600 | 6.62480800 | 6.50959500 |
| C | 5.66605600 | 8.18260000 | 4.62889100 |
| C | -3.58159800 | 0.24057600 | -0.24974000 |
| C | 3.84393600 | 0.36150600 | -0.41310000 |
| C | 4.46234200 | -0.51564200 | 0.47836900 |
| C | 5.15795600 | -1.62246400 | 0.02331800 |
| C | 5.26233900 | -1.89503700 | -1.34014100 |
| C | 4.67509400 | -0.99837000 | -2.23276600 |
| C | 3.96738500 | 0.10156200 | -1.77765700 |
| C | 5.99629600 | -3.09144700 | -1.84364700 |
| C | 5.77569700 | -4.33415200 | -1.36451200 |
| C | 6.63263800 | -5.50186200 | -1.72297400 |
| C | 4.65645300 | -4.64982000 | -0.42860700 |
| C | 6.98438700 | -2.80636200 | -2.92497700 |
| C | -3.46619300 | -0.95789400 | -0.94914300 |
| C | -4.27184300 | -2.04159500 | -0.64237600 |
| C | -5.21692200 | -1.96834900 | 0.38164900 |
| C | -5.33489000 | -0.76366100 | 1.07551400 |
| C | -4.52717400 | 0.32045200 | 0.76904200 |
| C | -6.12837100 | -3.10656000 | 0.69641000 |
| C | -5.71072600 | -4.38357500 | 0.82274500 |
| C | -7.56209900 | -2.72068700 | 0.86011700 |
| C | -6.65927900 | -5.53013300 | 0.94573300 |
| C | -4.27121500 | -4.77542900 | 0.84959000 |
| C | -8.27634000 | -3.06228800 | 2.00743700 |
| C | -9.60058000 | -2.67947700 | 2.15948100 |
| C | -10.23564200 | -1.95094900 | 1.16429600 |

| | | | |
|---|---|---|---|
| C | -9.53263300 | -1.59670300 | 0.02071900 |
| C | -8.20530700 | -1.96834800 | -0.12365200 |
| C | -3.81274300 | -5.80317600 | 0.02341500 |
| C | -2.48375800 | -6.19480100 | 0.04603900 |
| C | -1.59142000 | -5.58178700 | 0.91488400 |
| C | -2.03955300 | -4.57372000 | 1.75694000 |
| C | -3.36691600 | -4.17262500 | 1.72311600 |
| C | -6.49978700 | -6.46594300 | 1.96884600 |
| C | -7.36519100 | -7.54180700 | 2.08911600 |
| C | -8.39325000 | -7.71583700 | 1.17270900 |
| C | -8.54751200 | -6.80575100 | 0.13697000 |
| C | -7.68943900 | -5.72225100 | 0.02577800 |
| C | 6.97040000 | -3.51606200 | -4.12530200 |
| C | 7.87749400 | -3.22470200 | -5.13259700 |
| C | 8.81772600 | -2.21962200 | -4.95701500 |
| C | 8.83552300 | -1.49858900 | -3.77097700 |
| C | 7.91971600 | -1.78215900 | -2.77000600 |
| C | 6.04959500 | -6.69695600 | -2.14722000 |
| C | 6.83259700 | -7.79322500 | -2.47295600 |
| C | 8.21412800 | -7.72265100 | -2.35758300 |
| C | 8.80437100 | -6.54789600 | -1.91415400 |
| C | 8.02087300 | -5.44762500 | -1.60131100 |
| C | 4.90347200 | -5.36627100 | 0.74376800 |
| C | 3.87620800 | -5.66009700 | 1.62639200 |
| C | 2.57699600 | -5.26223200 | 1.34123400 |
| C | 2.31299000 | -4.57447600 | 0.16508700 |
| C | 3.34418700 | -4.27195800 | -0.71136600 |
| H | -0.04351600 | 6.47935300 | -2.79383700 |
| H | 0.80444000 | 4.70961100 | -1.35802900 |
| H | -5.68795500 | 5.51490600 | -4.26264300 |
| H | -1.88391700 | 7.47258400 | -4.06441100 |
| H | -5.21829000 | 1.60452900 | -1.68159500 |
| H | -6.05225500 | 3.40149900 | -3.10270100 |
| H | -6.10954700 | 8.43401000 | -6.23995300 |
| H | -5.94812300 | 6.67998600 | -6.17989400 |

| | | | |
|---|---|---|---|
| H | -6.48371500 | 7.57413000 | -4.74756900 |
| H | -4.58656700 | 9.93971000 | -5.04315600 |
| H | -3.21113300 | 9.33088400 | -4.12008700 |
| H | -4.84957900 | 9.09731600 | -3.50876000 |
| H | -3.54160100 | 6.91028200 | -7.00307100 |
| H | -3.81992600 | 8.65595000 | -7.09426400 |
| H | -2.43059400 | 8.02591300 | -6.20709300 |
| H | -0.41220700 | 1.47177900 | 2.30238000 |
| H | -2.03006800 | 0.73775500 | 2.31974300 |
| H | 1.33148100 | 0.82573000 | -1.04961300 |
| H | 6.35750600 | 3.46909900 | 2.56252600 |
| H | 5.57011100 | 1.79798100 | 0.98063700 |
| H | 1.97113000 | 7.06090600 | 4.23113800 |
| H | 5.89777200 | 5.36250400 | 4.03793600 |
| H | -0.64913400 | 4.50450600 | 1.27691800 |
| H | 0.15294700 | 6.12665300 | 2.90766600 |
| H | 3.88569700 | 9.04448300 | 6.46876300 |
| H | 3.04277700 | 8.92434900 | 4.92561300 |
| H | 2.63220100 | 7.82458700 | 6.25211100 |
| H | 5.47234000 | 7.33977100 | 7.24023000 |
| H | 5.89667100 | 5.94515700 | 6.24550600 |
| H | 4.30032200 | 6.03698900 | 6.99126900 |
| H | 5.30179900 | 8.72045900 | 3.75080800 |
| H | 6.05969600 | 8.91518600 | 5.33845800 |
| H | 6.49470700 | 7.54761100 | 4.31137500 |
| H | 4.37583700 | -0.34120400 | 1.54411900 |
| H | 5.61257100 | -2.29785200 | 0.73691200 |
| H | 4.76731700 | -1.17103400 | -3.29855400 |
| H | 3.50862000 | 0.77660000 | -2.49061400 |
| H | -2.74068600 | -1.03690900 | -1.74963900 |
| H | -4.16986700 | -2.95863800 | -1.20833000 |
| H | -6.07556800 | -0.67187400 | 1.86086800 |
| H | -4.64048600 | 1.24759500 | 1.31934400 |
| H | -7.78737000 | -3.63626700 | 2.78457700 |
| H | -10.13793000 | -2.95264400 | 3.05990400 |

| | | | |
|---|---|---|---|
| H | -11.27134200 | -1.65510700 | 1.28123200 |
| H | -10.01894500 | -1.02399700 | -0.76022200 |
| H | -7.65693500 | -1.67749800 | -1.01209500 |
| H | -4.50899300 | -6.29746300 | -0.64377900 |
| H | -2.14446400 | -6.98628700 | -0.61163500 |
| H | -0.55462900 | -5.89536900 | 0.93884500 |
| H | -1.35209700 | -4.09609300 | 2.44497800 |
| H | -3.70999100 | -3.38249400 | 2.37947500 |
| H | -5.68889200 | -6.34453300 | 2.67746300 |
| H | -7.23262600 | -8.25056200 | 2.89806700 |
| H | -9.06596900 | -8.56048900 | 1.26162300 |
| H | -9.34002100 | -6.93888400 | -0.58990800 |
| H | -7.81804600 | -5.01319400 | -0.78222200 |
| H | 6.24161800 | -4.30421300 | -4.26662700 |
| H | 7.84829600 | -3.78560300 | -6.05923300 |
| H | 9.52841300 | -1.99401000 | -5.74308200 |
| H | 9.56197600 | -0.70751800 | -3.62687600 |
| H | 7.92797900 | -1.20558500 | -1.85244600 |
| H | 4.97066200 | -6.76303600 | -2.22392500 |
| H | 6.36176500 | -8.70821500 | -2.81268000 |
| H | 8.82667400 | -8.58134400 | -2.60524200 |
| H | 9.88119700 | -6.48640100 | -1.81016900 |
| H | 8.48780600 | -4.53239600 | -1.25986700 |
| H | 5.91386600 | -5.68932900 | 0.96534900 |
| H | 4.09014200 | -6.20416200 | 2.53881100 |
| H | 1.77418500 | -5.49168000 | 2.03218900 |
| H | 1.30065000 | -4.26823600 | -0.07073500 |
| H | 3.13321700 | -3.72876700 | -1.62385700 |
| C | -0.41489900 | -0.66301100 | 2.63245300 |
| H | -1.00311300 | -1.51964000 | 2.29030200 |
| H | 0.60091500 | -0.80458500 | 2.25173700 |
| C | -0.40046700 | -0.60186400 | 4.15653000 |
| H | 0.19333500 | 0.25999800 | 4.48244600 |
| H | -1.41755300 | -0.43008400 | 4.52875300 |
| C | 0.15988000 | -1.86596500 | 4.80262600 |

| | | | |
|---|---|---|---|
| H | -0.44521400 | -2.72578000 | 4.49462700 |
| H | 1.16836600 | -2.04983500 | 4.41751100 |
| C | 0.19840900 | -1.79121700 | 6.32499400 |
| H | -0.80239300 | -1.63767300 | 6.73743500 |
| H | 0.60012400 | -2.70818100 | 6.76124300 |
| H | 0.82461700 | -0.96089300 | 6.66175900 |

Total Energy (CAM-RB3LYP) = -3818.515304 Hartree

**Table S11.** Atom coordinates and absolute energies for ***R*-Bipyrene-OH** Standard orientation

| AtomicType | Coordinates(Angstroms) | | |
|---|---|---|---|
| | X | Y | Z |
| C | -1.91335700 | -2.98209900 | -1.72193200 |
| C | -3.02734800 | -2.17474500 | -1.50398200 |
| C | -2.95000700 | -1.14735200 | -0.53584600 |
| C | -1.75098200 | -0.95557100 | 0.19933500 |
| C | -0.64515500 | -1.78432600 | -0.03258700 |
| C | -0.74627000 | -2.78904000 | -0.99988900 |
| C | -4.07753800 | -0.31011600 | -0.30426900 |
| C | -4.01522400 | 0.72621400 | 0.65635800 |
| C | -2.78647000 | 0.90585900 | 1.38005600 |
| C | -1.71639000 | 0.11399100 | 1.16577400 |
| C | -5.27347100 | -0.50006400 | -1.03073700 |
| C | -6.36744100 | 0.33434300 | -0.78552700 |
| C | -6.31989100 | 1.35527300 | 0.15603300 |
| C | -5.13015300 | 1.53130700 | 0.86470400 |
| C | -4.25423500 | -2.34900800 | -2.23069000 |
| C | -5.32094700 | -1.55388600 | -2.00489200 |
| C | -7.51084600 | 2.27925700 | 0.43644800 |
| C | -8.72684300 | 1.94083900 | -0.43180500 |
| C | -7.10772100 | 3.73525100 | 0.14815000 |
| C | -7.92473700 | 2.14670700 | 1.91177700 |
| O | 0.35273500 | -3.56539000 | -1.19860300 |
| C | 0.62160400 | -1.64066400 | 0.74194100 |
| C | 1.72573900 | -0.94844200 | 0.21853900 |
| C | 2.91006400 | -0.84423600 | 0.99142000 |
| C | 2.97925300 | -1.43232600 | 2.27796300 |
| C | 1.86530700 | -2.11036800 | 2.76605200 |
| C | 0.71089700 | -2.20954100 | 2.00781400 |
| C | 4.03888300 | -0.14335100 | 0.47655100 |
| C | 5.22632200 | -0.03478500 | 1.24036100 |
| C | 5.26330700 | -0.64583700 | 2.54216500 |
| C | 4.19691200 | -1.30790600 | 3.03306500 |
| C | 3.98892200 | 0.45250400 | -0.80192600 |

| | | | |
|---|---|---|---|
| C | 5.10719600 | 1.13524600 | -1.28762200 |
| C | 6.27882400 | 1.24890700 | -0.54908300 |
| C | 6.31374600 | 0.65467200 | 0.71335700 |
| C | 1.70494600 | -0.32943300 | -1.07916200 |
| C | 2.77620500 | 0.33434100 | -1.56111400 |
| C | 7.51451000 | 1.99375500 | -1.06791600 |
| C | 7.29561600 | 2.58185800 | -2.46553400 |
| C | 8.70459600 | 1.02204700 | -1.13899900 |
| C | 7.85482700 | 3.14869500 | -0.11097800 |
| H | -2.73322500 | 1.70490300 | 2.11137400 |
| H | -0.80208500 | 0.27350000 | 1.72179700 |
| H | -7.26874300 | 0.16189700 | -1.35885800 |
| H | -5.05500800 | 2.31842600 | 1.60638700 |
| H | -4.30591000 | -3.13996200 | -2.97059000 |
| H | -6.23995100 | -1.69925800 | -2.56192500 |
| H | -9.54533300 | 2.62409900 | -0.19526000 |
| H | -9.08354800 | 0.92419200 | -0.25223600 |
| H | -8.50705300 | 2.04559900 | -1.49659000 |
| H | -7.94793800 | 4.40666400 | 0.34412000 |
| H | -6.27230400 | 4.05624400 | 0.77244400 |
| H | -6.81053700 | 3.85708000 | -0.89581900 |
| H | -8.21798300 | 1.12020700 | 2.14274000 |
| H | -8.77395700 | 2.80065800 | 2.12703300 |
| H | -7.11298000 | 2.42177500 | 2.58712700 |
| H | -0.14586600 | -2.74425100 | 2.40047800 |
| H | 6.17431000 | -0.56143400 | 3.12422500 |
| H | 4.23750700 | -1.76395300 | 4.01602200 |
| H | 5.03535900 | 1.58074900 | -2.27094700 |
| H | 7.21118000 | 0.72315900 | 1.31763500 |
| H | 0.79967300 | -0.40262000 | -1.66800500 |
| H | 2.73691300 | 0.79511700 | -2.54203200 |
| H | 8.20085000 | 3.10037500 | -2.78880000 |
| H | 6.47820200 | 3.30618100 | -2.47763900 |
| H | 7.07865600 | 1.80585700 | -3.20290000 |
| H | 9.59417000 | 1.53936700 | -1.50813600 |

| | | | |
|---|---|---|---|
| H | 8.94484100 | 0.60258000 | -0.16072600 |
| H | 8.48850800 | 0.19140600 | -1.81454800 |
| H | 7.02446000 | 3.85503500 | -0.04324500 |
| H | 8.73471800 | 3.68998400 | -0.46875600 |
| H | 8.07105000 | 2.79025600 | 0.89671000 |
| H | 1.90802400 | -2.56558700 | 3.74918800 |
| H | -1.96323400 | -3.77482000 | -2.46274700 |
| H | 0.16946200 | -4.20811600 | -1.88893000 |

Total Energy (CAM-RB3LYP) = -1619.520470 Hartree

**Table S12.** Atom coordinates and absolute energies for ***R*-3** Standard orientation

| AtomicType | Coordinates(Angstroms) | | |
|---|---|---|---|
| | X | Y | Z |
| C | 2.15138800 | 3.27294400 | -0.28831600 |
| C | 3.26129700 | 2.44162000 | -0.40558200 |
| C | 3.16632600 | 1.10741800 | 0.05707000 |
| C | 1.95632200 | 0.63450600 | 0.62736700 |
| C | 0.84429200 | 1.49006300 | 0.71781100 |
| C | 0.97145100 | 2.80745700 | 0.26566600 |
| C | 4.29752500 | 0.24718800 | -0.04348800 |
| C | 4.22912900 | -1.08492300 | 0.42657500 |
| C | 2.99746900 | -1.53331800 | 1.01455800 |
| C | 1.92444700 | -0.72243600 | 1.11204700 |
| C | 5.50403600 | 0.71211800 | -0.61229700 |
| C | 6.59839000 | -0.15137000 | -0.70385800 |
| C | 6.54314800 | -1.46380500 | -0.24888900 |
| C | 5.34609500 | -1.90717900 | 0.31424600 |
| C | 4.50035800 | 2.89225200 | -0.97657700 |
| C | 5.56486600 | 2.07006600 | -1.07725000 |
| C | 7.73591200 | -2.42263000 | -0.34266000 |
| C | 8.96222300 | -1.76519000 | -0.98380100 |
| C | 7.34578600 | -3.64158600 | -1.19554700 |
| C | 8.12767700 | -2.89134700 | 1.06883800 |
| O | -0.07318300 | 3.68639400 | 0.40331400 |
| C | -1.02704900 | 3.67480700 | -0.65987100 |
| C | -0.44177000 | 1.04692600 | 1.32989000 |
| C | -1.30560800 | 0.14862200 | 0.67856500 |
| C | -2.51221300 | -0.24226100 | 1.31619600 |
| C | -2.84504500 | 0.27014000 | 2.59334400 |
| C | -1.97243300 | 1.16876100 | 3.20335800 |
| C | -0.79878000 | 1.54919200 | 2.57807200 |
| C | -3.39953100 | -1.15271900 | 0.67154400 |
| C | -4.60253100 | -1.55187400 | 1.30312600 |
| C | -4.90611000 | -1.01664600 | 2.60298700 |
| C | -4.07193900 | -0.15138600 | 3.21317900 |

| | | | |
|---|---|---|---|
| C | -3.09360800 | -1.66781700 | -0.60638400 |
| C | -3.97693400 | -2.55905400 | -1.22055900 |
| C | -5.15953600 | -2.96103400 | -0.61100300 |
| C | -5.45007100 | -2.44269700 | 0.65161100 |
| C | -1.02676700 | -0.38853500 | -0.62787600 |
| C | -1.87335100 | -1.24671300 | -1.23438200 |
| C | -6.13933900 | -3.93875400 | -1.27038800 |
| C | -5.65948600 | -4.40222900 | -2.64949000 |
| C | -7.50555600 | -3.25413700 | -1.44435800 |
| C | -6.30049300 | -5.18069900 | -0.37754800 |
| H | 2.94625900 | -2.54981700 | 1.38881500 |
| H | 1.01255600 | -1.08546200 | 1.56672200 |
| H | 7.50729500 | 0.23564200 | -1.14513200 |
| H | 5.26612000 | -2.92336800 | 0.68324800 |
| H | 4.56018700 | 3.91627200 | -1.32755200 |
| H | 6.49299900 | 2.42420700 | -1.51199200 |
| H | 9.78130400 | -2.48623600 | -1.02764400 |
| H | 9.31017000 | -0.90551600 | -0.40682600 |
| H | 8.75854900 | -1.43495000 | -2.00482300 |
| H | 8.18720100 | -4.33541900 | -1.27135400 |
| H | 6.50340300 | -4.18512600 | -0.76469600 |
| H | 7.06448200 | -3.33560200 | -2.20564600 |
| H | 8.41042200 | -2.04295200 | 1.69595900 |
| H | 8.97839100 | -3.57602600 | 1.01765000 |
| H | 7.30850100 | -3.41447900 | 1.56476700 |
| H | -1.44407700 | 2.66840900 | -0.77344900 |
| H | -0.52609200 | 3.94336300 | -1.59862900 |
| H | -0.13460600 | 2.25288100 | 3.06395500 |
| H | -5.82758700 | -1.32506100 | 3.08412600 |
| H | -4.31272400 | 0.24705500 | 4.19245500 |
| H | -3.71152500 | -2.93430600 | -2.20000000 |
| H | -6.36414100 | -2.73314100 | 1.15686100 |
| H | -0.11334900 | -0.08785600 | -1.12422400 |
| H | -1.64067200 | -1.63605200 | -2.21939800 |
| H | -6.38873400 | -5.09461100 | -3.07551500 |

| | | | |
|---|---|---|---|
| H | -4.70249600 | -4.92546500 | -2.59166400 |
| H | -5.55328900 | -3.56629400 | -3.34441600 |
| H | -8.21521300 | -3.93995600 | -1.91458600 |
| H | -7.92790300 | -2.94204400 | -0.48784900 |
| H | -7.41876400 | -2.36763200 | -2.07650600 |
| H | -5.34271000 | -5.68714100 | -0.23905500 |
| H | -6.99705200 | -5.88798000 | -0.83542100 |
| H | -6.68778500 | -4.92343200 | 0.60963600 |
| C | -2.11600900 | 4.66980900 | -0.32436300 |
| H | -1.65706500 | 5.65173200 | -0.17377600 |
| H | -2.56470500 | 4.38074300 | 0.63042100 |
| C | -3.18856400 | 4.74973500 | -1.40587100 |
| H | -3.63175100 | 3.75847400 | -1.55615300 |
| H | -2.72768900 | 5.02643600 | -2.36166600 |
| C | -4.29603200 | 5.74752200 | -1.07871000 |
| H | -3.85426100 | 6.73824000 | -0.92735800 |
| H | -4.75733200 | 5.47101200 | -0.12481000 |
| C | -5.36705400 | 5.82497100 | -2.16105200 |
| H | -4.93781700 | 6.13053400 | -3.11903200 |
| H | -6.14650800 | 6.54462000 | -1.90218300 |
| H | -5.84721700 | 4.85417400 | -2.30962000 |
| H | -2.22006300 | 1.57112400 | 4.17921400 |
| H | 2.19680300 | 4.30518800 | -0.61495500 |

Total Energy (CAM-RB3LYP) = -1816.004626 Hartree

**Table S13.** Atom coordinates and absolute energies for **Py-TPE** Standard orientation

| AtomicType | Coordinates(Angstroms) | | |
|---|---|---|---|
| | X | Y | Z |
| C | 1.56574000 | 1.07453300 | -1.29121700 |
| C | 2.67148000 | 0.45451100 | -0.67716500 |
| C | 3.97889900 | 0.93314400 | -0.95713000 |
| C | 4.17204800 | 2.01226100 | -1.85412600 |
| C | 3.05875400 | 2.58446900 | -2.46358000 |
| C | 1.78606100 | 2.12062200 | -2.18473700 |
| C | 5.11164600 | 0.32705600 | -0.33862400 |
| C | 6.41809300 | 0.80693600 | -0.60204100 |
| C | 6.57493300 | 1.91023000 | -1.51093400 |
| C | 5.50815400 | 2.47900800 | -2.10627100 |
| C | 2.54454000 | -0.67690400 | 0.20374400 |
| C | 3.62120700 | -1.24951600 | 0.78169300 |
| C | 4.94930000 | -0.76262000 | 0.54322200 |
| C | 6.07187800 | -1.34011000 | 1.14224500 |
| C | 7.35792000 | -0.87461400 | 0.89757500 |
| C | 7.50489500 | 0.20037900 | 0.01977700 |
| C | 0.16422800 | 0.66065900 | -1.01807800 |
| C | -0.36862400 | 0.69536400 | 0.27045800 |
| C | -1.68978200 | 0.35330500 | 0.50522000 |
| C | -2.52706700 | -0.04003800 | -0.53836600 |
| C | -1.98797100 | -0.09455000 | -1.82365200 |
| C | -0.67130500 | 0.26317800 | -2.06136700 |
| C | -3.94375700 | -0.43839900 | -0.29563200 |
| C | -4.80387700 | 0.31820500 | 0.41801600 |
| C | -4.33439900 | -1.74201500 | -0.90863400 |
| C | -6.15317400 | -0.16626800 | 0.83211600 |
| C | -4.48356200 | 1.70821800 | 0.85638200 |
| C | -4.67571700 | 2.08853600 | 2.18544100 |
| C | -4.39154600 | 3.37921000 | 2.60313200 |
| C | -3.93226200 | 4.32163800 | 1.69330600 |
| C | -3.76056400 | 3.96250600 | 0.36417600 |
| C | -4.03239800 | 2.66746600 | -0.04975500 |

| | | | |
|---|---|---|---|
| C | -5.46359500 | -1.85114800 | -1.71959500 |
| C | -5.80308100 | -3.06149600 | -2.30456500 |
| C | -5.02078300 | -4.18650300 | -2.08690000 |
| C | -3.88836900 | -4.08898100 | -1.29001300 |
| C | -3.54294900 | -2.87539000 | -0.71650700 |
| C | -7.28236400 | 0.62348700 | 0.60994600 |
| C | -8.54006100 | 0.19061000 | 0.99959600 |
| C | -8.68946100 | -1.03121300 | 1.64124600 |
| C | -7.57161200 | -1.81473800 | 1.88935700 |
| C | -6.31571900 | -1.38671500 | 1.48712800 |
| C | 8.59993900 | -1.49445200 | 1.54853500 |
| C | 8.24905300 | -2.66069600 | 2.47798200 |
| C | 9.54225000 | -2.02217800 | 0.45357000 |
| C | 9.32945700 | -0.42437500 | 2.37816700 |
| H | 3.19528300 | 3.40941500 | -3.15375800 |
| H | 0.93021700 | 2.59431400 | -2.65076500 |
| H | 7.57647800 | 2.27476300 | -1.71023000 |
| H | 5.63823700 | 3.30810300 | -2.79287500 |
| H | 1.55793900 | -1.07814100 | 0.39021800 |
| H | 3.49669200 | -2.10463000 | 1.43692600 |
| H | 5.91020200 | -2.17421200 | 1.81206600 |
| H | 8.49347600 | 0.58846500 | -0.19752100 |
| H | 0.25708300 | 1.00984900 | 1.09727800 |
| H | -2.08253900 | 0.39928600 | 1.51309500 |
| H | -2.61008200 | -0.42259200 | -2.64806100 |
| H | -0.27578200 | 0.21641400 | -3.06935000 |
| H | -5.04931700 | 1.36137200 | 2.89679800 |
| H | -4.53464000 | 3.65180400 | 3.64211400 |
| H | -3.71603300 | 5.33255100 | 2.01754600 |
| H | -3.41338500 | 4.69419600 | -0.35570700 |
| H | -3.89283500 | 2.39173800 | -1.08750400 |
| H | -6.08048200 | -0.97770400 | -1.88947400 |
| H | -6.68292700 | -3.12492700 | -2.93382900 |
| H | -5.28779400 | -5.13300600 | -2.54155700 |
| H | -3.26765200 | -4.96060900 | -1.11896300 |

| | | | |
|---|---|---|---|
| H | -2.64927700 | -2.80167700 | -0.10797900 |
| H | -7.16987200 | 1.58557700 | 0.12409300 |
| H | -9.40624500 | 0.81259000 | 0.80681200 |
| H | -9.67130300 | -1.36725500 | 1.95263200 |
| H | -7.67638200 | -2.76491300 | 2.39958200 |
| H | -5.44782100 | -2.00462000 | 1.67993300 |
| H | 9.16381300 | -3.06569000 | 2.91607700 |
| H | 7.60182200 | -2.34476700 | 3.29911000 |
| H | 7.75291200 | -3.47233700 | 1.94127100 |
| H | 10.43280900 | -2.46858700 | 0.90383800 |
| H | 9.87115600 | -1.22663200 | -0.21694300 |
| H | 9.04718100 | -2.78533000 | -0.15100700 |
| H | 8.68086500 | -0.03343000 | 3.16525300 |
| H | 10.21820000 | -0.85204900 | 2.84971700 |
| H | 9.65116100 | 0.41700300 | 1.76231800 |

Total Energy (CAM-RB3LYP) = -1774.002627 Hartree

## References

(1) Crawford, A. G.; Liu, Z.; Mkhalid, I. A. I.; Thibault, M. H.; Schwarz, N.; Alcaraz, G.; Steffen, A.; Collings, J. C.; Batsanov, A. S.; Howard, J. A. K.; Marder, T. B. *Chem. Eur. J.* **2012**, *18*, 5022-5035

(2) Liu, Y.; Zhang, J.; Wang, X.; Xie, Z.; Zheng, H.; Zhang, S.; Cai, X.; Zhao, Y.; Redshaw, C.; Min, Y.; Feng, X. *J. Org. Chem.* **2024**, *89*, 3319-3330.

(3) Niu, X.; Ou, X.; Ren, S.; Wang, K.; Song, F.; Dong, X.; Guo, W. J.; Peng, H. Q.; Zhao, Z.; Lam, J. W. Y.; Zhao, Y. S.; Li, F.; Yu, S. Y.; Tang, B. Z. *Aggregate* **2025**, *6*, e70003.

(4) Hassan, K.; Yamashita, K.; Hirabayashi, K.; Shimizu, T.; Nakabayashi, K.; Imai, Y.; Matsumoto, T.; Yamano, A.; Sugiura, K. *Chem. Lett.* **2015**, *44*, 1607–1609.

(5) Yang, W.; Longhi, G.; Abbate, S.; Lucotti, A.; Tommasini, M.; Villani, C.; Catalano, V. J.; Lykhin, A. O.; Varganov, S. A.; Chalifoux, W. A. *J. Am. Chem. Soc.* **2017**, *139*, 13102-13109.

(6) Takaishi, K.; Iwachido, K.; Ema, T. *J. Am. Chem. Soc.* **2020**, *142*, 1774-1779.

(7) Bam, R.; Yang, W.; Longhi, G.; Abbate, S.; Lucotti, A.; Tommasini, M.; Franzini, R.; Villani, C.; Catalano, V. J.; Olmstead, M. M.; Chalifoux, W. A. *Angew. Chem. In. Ed.* **2024**, *63*, e202404849.

(8) Oh, J.; Lee, Y.; Noh, G.; Park, D.; Lee, H.; Lee, J.; Kim, C.; Seo, J. *Small Struct.* **2024**, *5*, 2300396.

(9) Swain, A. K.; Kolanji, K.; Stapper, C.; Ravat, P. *Org. Lett.* **2021**, *23*, 1339-1343.

(10) Wang, C.; Jiang, T.; Ma, X. *Chin. Chem. Lett.* **2020**, *31*, 2921-2924.

(11) Zhang, H.; Li, H.; Wang, J.; Sun, J.; Qin, A.; Tang, B. Z. *J. Mater. Chem. C.* **2015**, *3*, 5162-5166.

(12) Zhang, X.; Zhang, Y.; Zhang, H.; Quan, Y.; Li, Y.; Cheng, Y.; Ye, S. *Org. Lett.* **2019**, *21*, 439–443.

(13) Feng, H.; Zhu, L.; Gao, H.; Yang, W.; Feng, Y. S. *Dyes. Pigm.* **2025**, *240*, 112858.
